# Supplementary material for: Joint multi-ancestry and admixed GWAS reveals the complex genetics behind human cranial vault shape
Source: Nat Commun. 2023 Nov 16;14:7436. doi: 10.1038/s41467-023-43237-8 (PMC10654897; doi:10.1038/s41467-023-43237-8)
Supplement: Supplementary file 1 — Supplementary Information [file 41467_2023_43237_MOESM1_ESM.pdf]

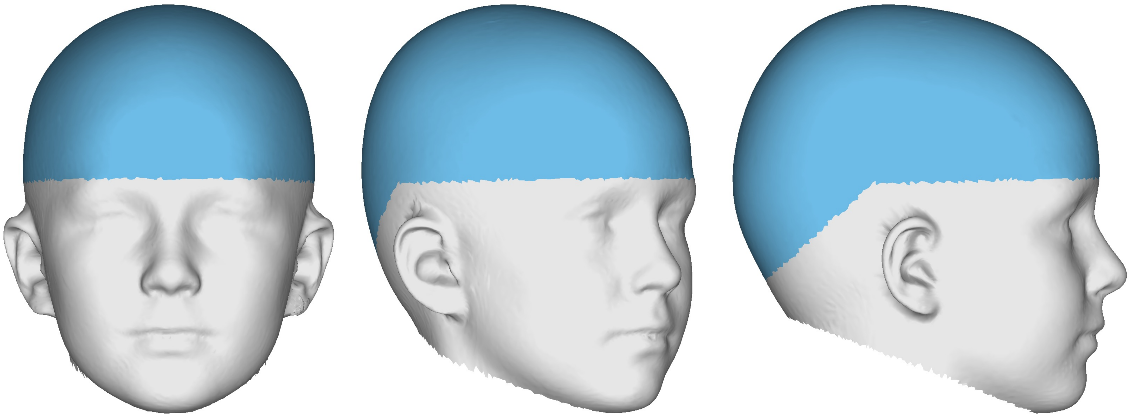

Supplementary Fig. 1 Cranial vault surface atlas. The cranial vault surface (cyan) as defined in this study, encompassing the supraorbital ridge and extending towards the occipital bone. Depicted on the full head mesh template.

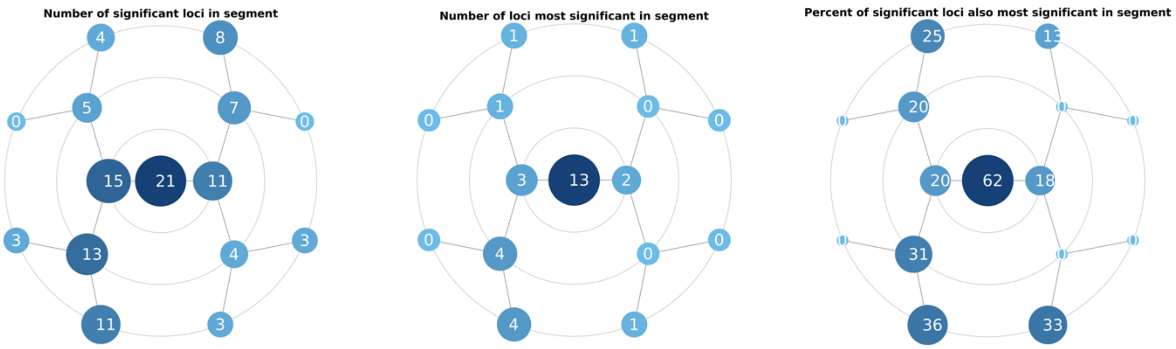

Supplementary Fig. 2 Localization of genome-wide significant cranial vault shape loci. Significance is declared at P < 5e-8. Left to right: the number of significant loci in each cranial vault segment; the number of loci reaching their lowest P value in each segment; the percentage of significant loci also being the most significant in each segment.

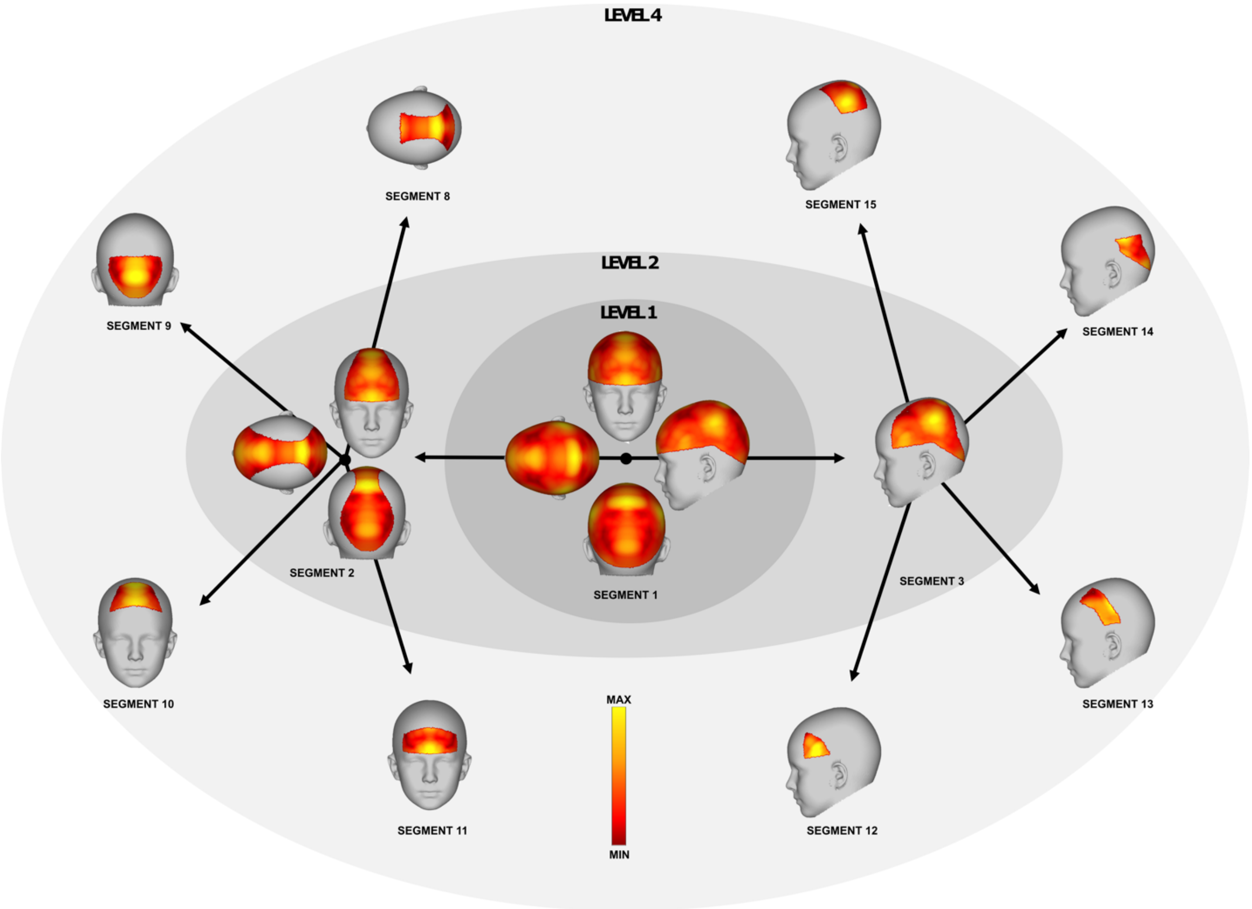

Supplementary Fig. 3 Deformation hotspots of the cranial vault are separated by global-to-local segmentation. Vertex-wise normal distances were calculated from the effects of each of 22 SNPs with genome-wide significant ($P < 5e\text{-}8$) effects on global cranial vault shape yielding a distribution of normal displacements associated with each vertex. The colormap shows the 95th percentile of this distribution for each vertex. Yellow spots represent deformation hotspots, i.e., regions where shape deformations were observed to be larger than surrounding regions. The global-to-local segmentation was then overlayed to illustrate how it relates to these deformation hotspots. The colormap applies to each segment individually, ranging between the minimum and maximum values. Hierarchical level 3 is omitted to declutter the figure.

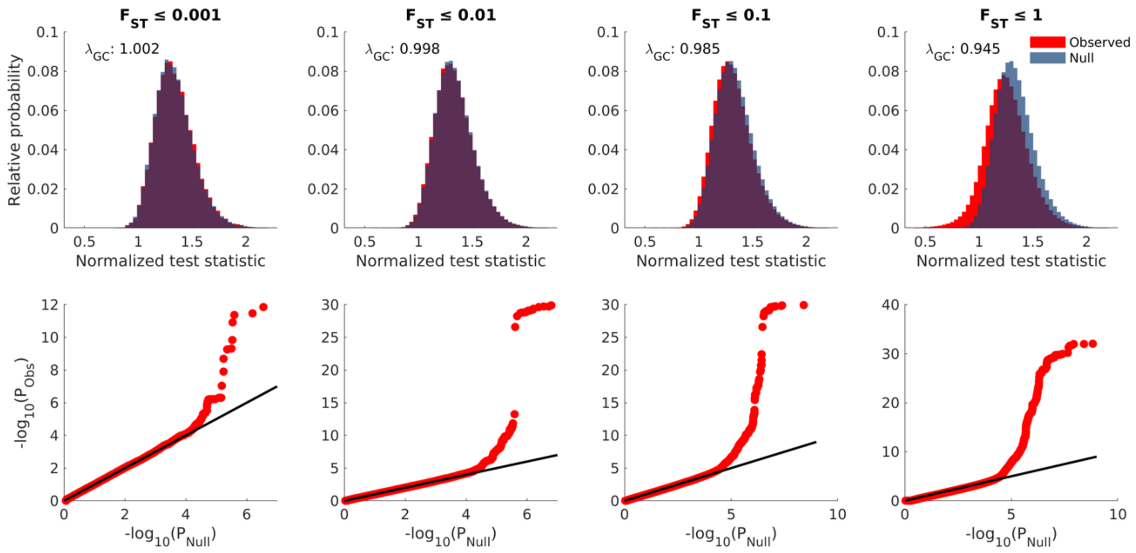

Supplementary Fig. 4 Observed versus null-distributed genotype-phenotype associations across the F_{ST} spectrum. Distributions of observed (red) and null-distributed (blue) normalized test statistics (top row) with corresponding λ_{GC} values and QQ-plots (bottom row) for subsets of SNPs statisfying indicated F_{ST} thresholds. For each SNP, both the observed and null-distributed P-values together with their test statistics represent the best genotype-phenotype association across the 15 cranial vault segments (Methods). Panels for $F_{ST} \leq 1$ represent the full GWAS.

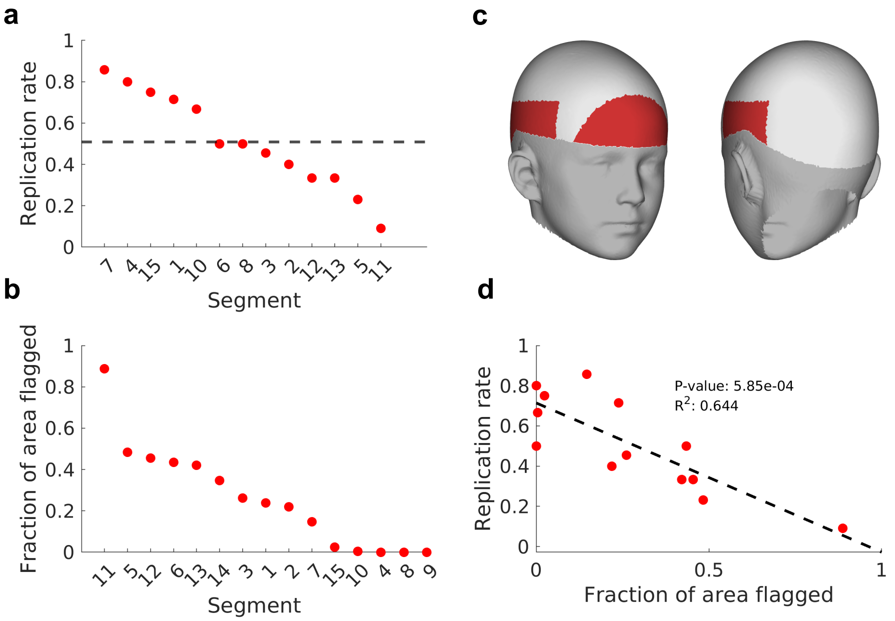

Supplementary Fig. 5 Segment-wise replication rate in the UK Biobank. a, Fraction of significant (P < 5e-8) loci per cranial vault segment that replicated at 5% FDR. Dashed line represents the overall replication rate (55/108 SNP-segment pairs). No significant loci were found to be associated with segments 9 and 14 during discovery. Segments 5 and its direct descendent, segment 11, both contain the forehead, which was severely damaged in the UK Biobank sample. **b**, Fraction of vertices within each cranial vault segment that were flagged, i.e., located within any of the damage-harboring regions. **c**, Damage harboring regions (red) of the cranial vault (light grey). **d**, The fraction of vertices located in damage-harboring regions was significantly associated with the replication rate (P-value = 5.85e-4, two-tailed t-test). Each point represents one cranial vault segment.

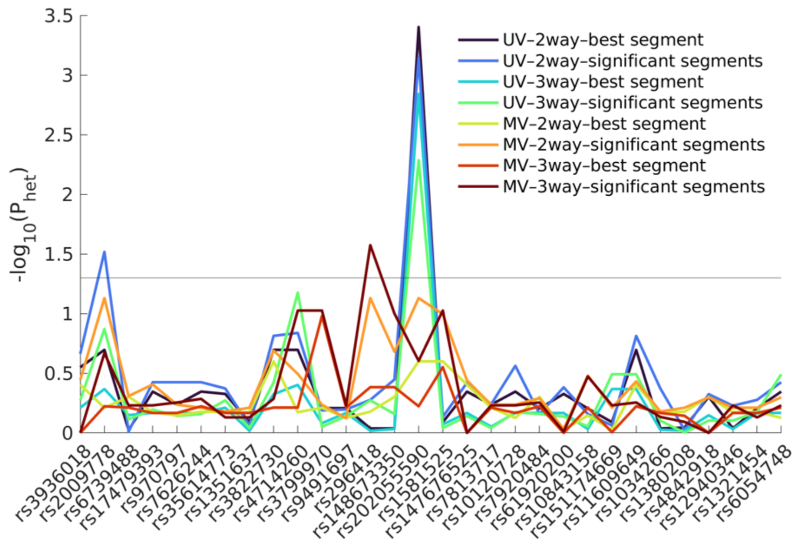

Supplementary Fig. 6 Effect size heterogeneity of genome-wide cranial vault shape loci. In total, we used 2 × 2 × 2 scenarios to test effect size heterogeneity. **UV/MV:** Under the 'univariate (UV) scenario', the latent associated shape trait from the main GWAS was tested. Under the 'multivariate (MV) scenario', the shape trait was free to vary. **2way/3way:** Under the '2-way scenario', only African-European effect size heterogeneity was tested. Under the '3-way scenario', African-European-Indigenous American effect size heterogeneity was tested. **Best segment/significant segments:** Under the 'best segment scenario', heterogeneity of effect size was only tested in the segment most significantly associated with the SNP in the GWAS. Under the 'significant segments scenario', heterogeneity of effect size was tested in all segments where that SNP was significant ($P < 5e\text{-}8$) in the main GWAS, and the lowest P-value was kept. P-values were adjusted for 5% FDR, and the horizontal line represents the $P < 0.05$ significance threshold. SNPs are in genomic order.

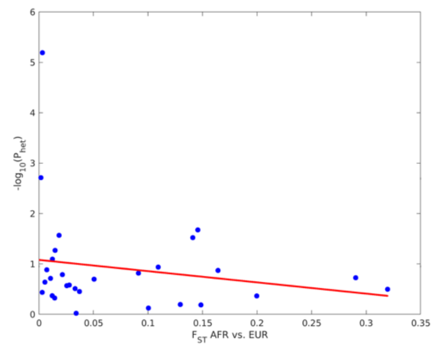

Supplementary Fig. 7 Relationship between F_{ST} and effect size heterogeneity. Heterogeneity of effect size between European and African ancestry based on the univariate latent phenotypes associated with each SNP in the main GWAS. All segment-SNP combinations with $P < 5e\text{-}8$ during GWAS discovery were considered, and the lowest P-value for each SNP was kept after adjustment for 5% FDR. ANOVA F-test was insignificant at 5% alpha (P-value: 0.297, R^2: 0.039).

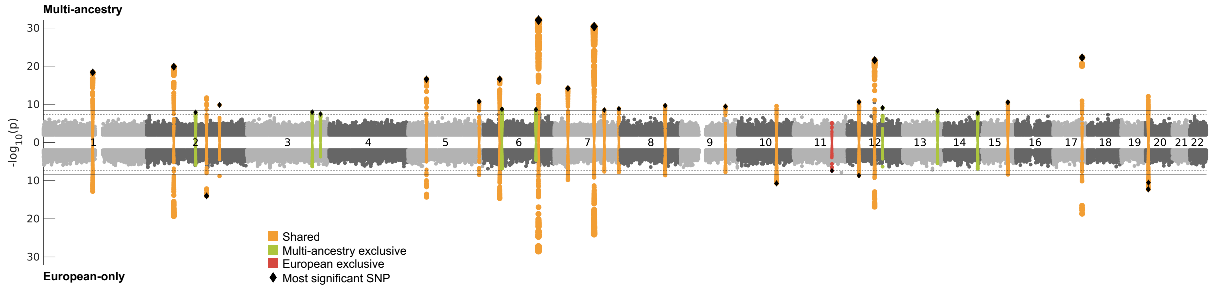

Supplementary Fig. 8 Genome-wide cranial vault shape loci from multi-ancestry and European-only GWAS. Miami plot shows the multi-ancestry (top; n = 6,772) and European-only GWAS (bottom; n = 4,198). Loci are colored based on whether they were shared (orange; lead SNPs within 250 kb); significant only in the multi-ancestry GWAS (green); or significant only in the European-only GWAS (red). Solid black diamonds indicate the most significantly association SNP at each locus and across both GWASs.

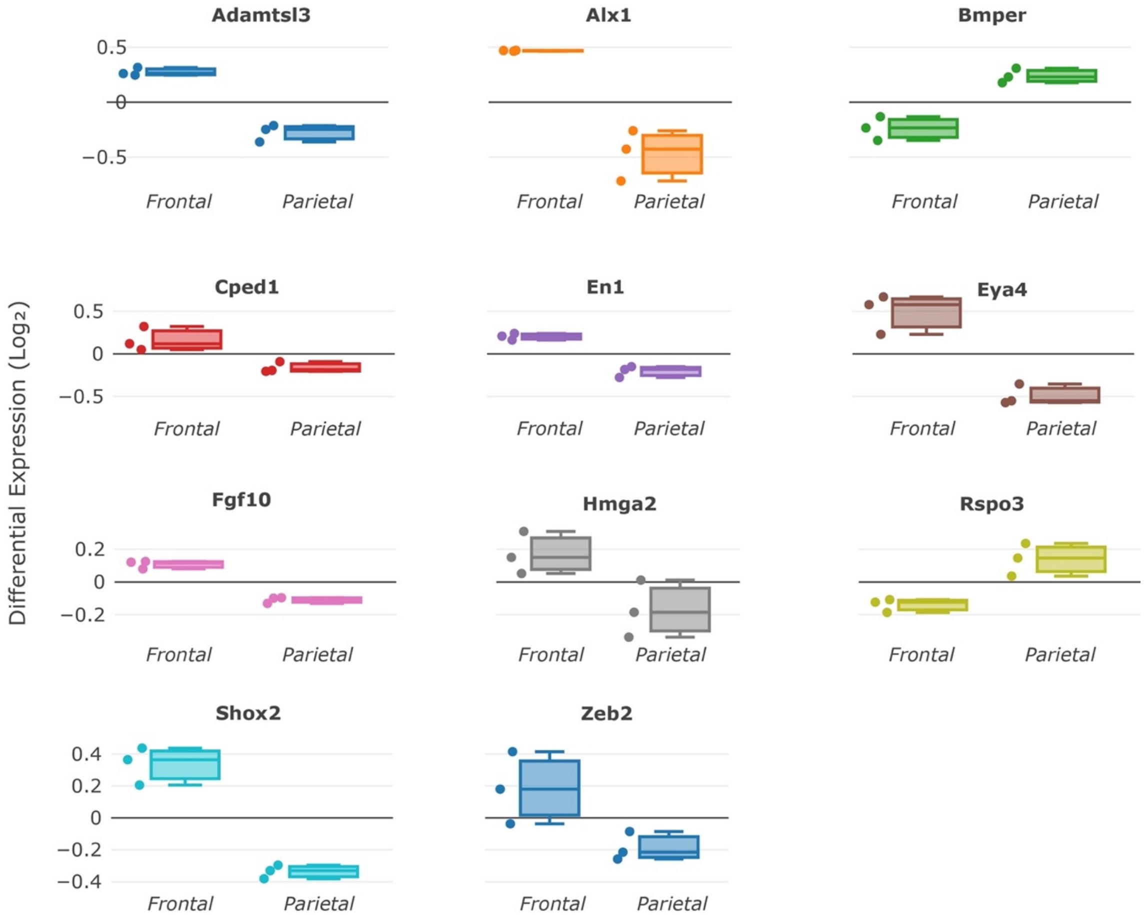

Supplementary Fig. 9 GWAS Candidate Differentially Expressed Genes (DEGs). DEGs raw counts were VST transformed, housekeeping gene normalized, and shown as the mean difference of DEG sample groups to the overall mean expression across all (parietal and frontal) sampling groups. Boxplots plot the first and third quartiles, with an internal horizontal line representing the median. Whiskers extend to the largest and smallest values.

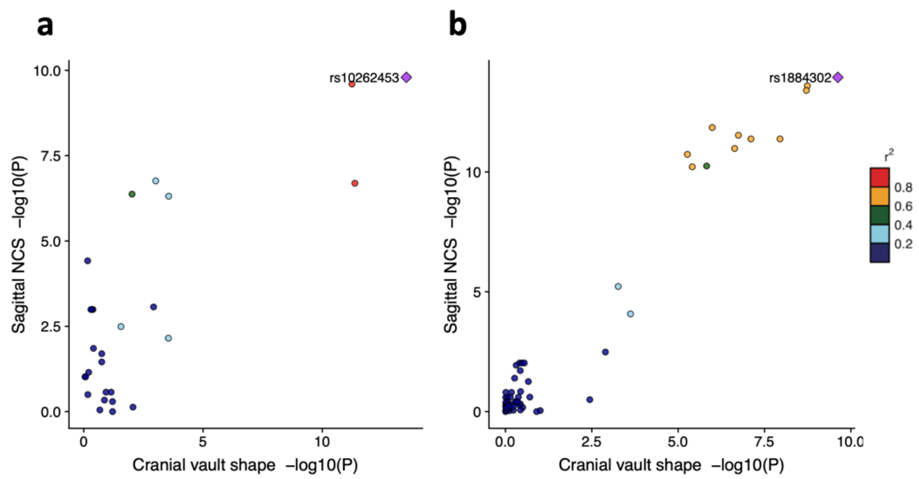

Supplementary Fig. 10 LocusCompare plots of cranial vault shape and sagittal non-syndromic craniosynostosis for SNPs in common with Justice et al. (2012). a, LocusCompare plot around rs10262453 near *BBS9*. **b**, LocusCompare plot around rs1884302 near *BMP2*. Color represents LD (r^2) with the lead SNP (purple diamond) from Justice et al. (2012).

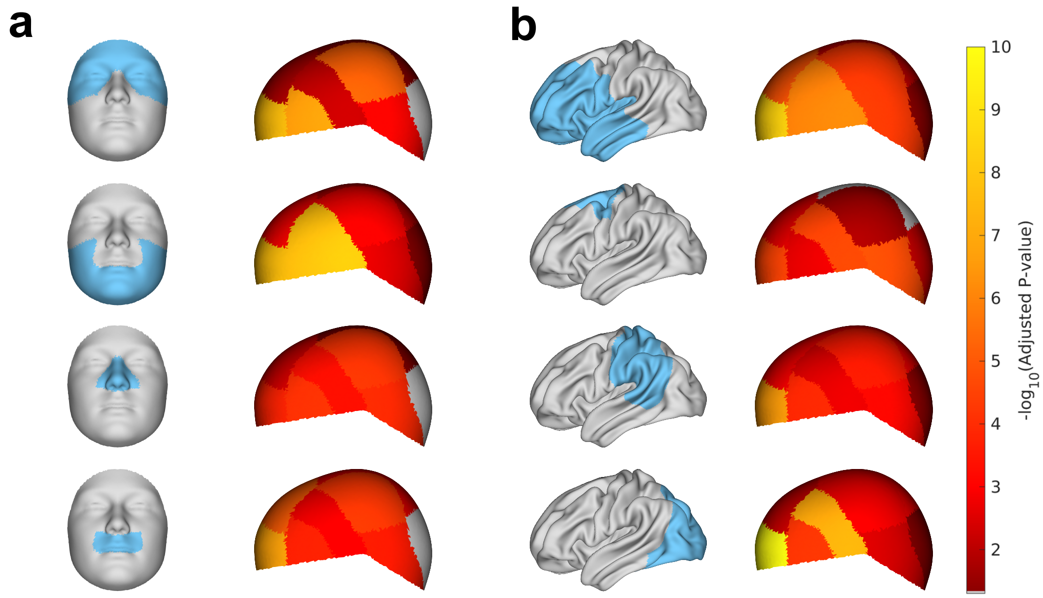

Supplementary Fig. 11 Genome-wide sharing of signals between the cranial vault, brain, and face. a, Genetic Spearman correlations with individual level-3 face (**a**) and brain (**b**) segments (cyan) across level-4 vault segments. Significance of Spearman correlations was determined based on standard errors obtained through bootstrapping. Empirical P-values (one-tailed) were adjusted for 5% FDR. Insignificant segments are indicated in grey.

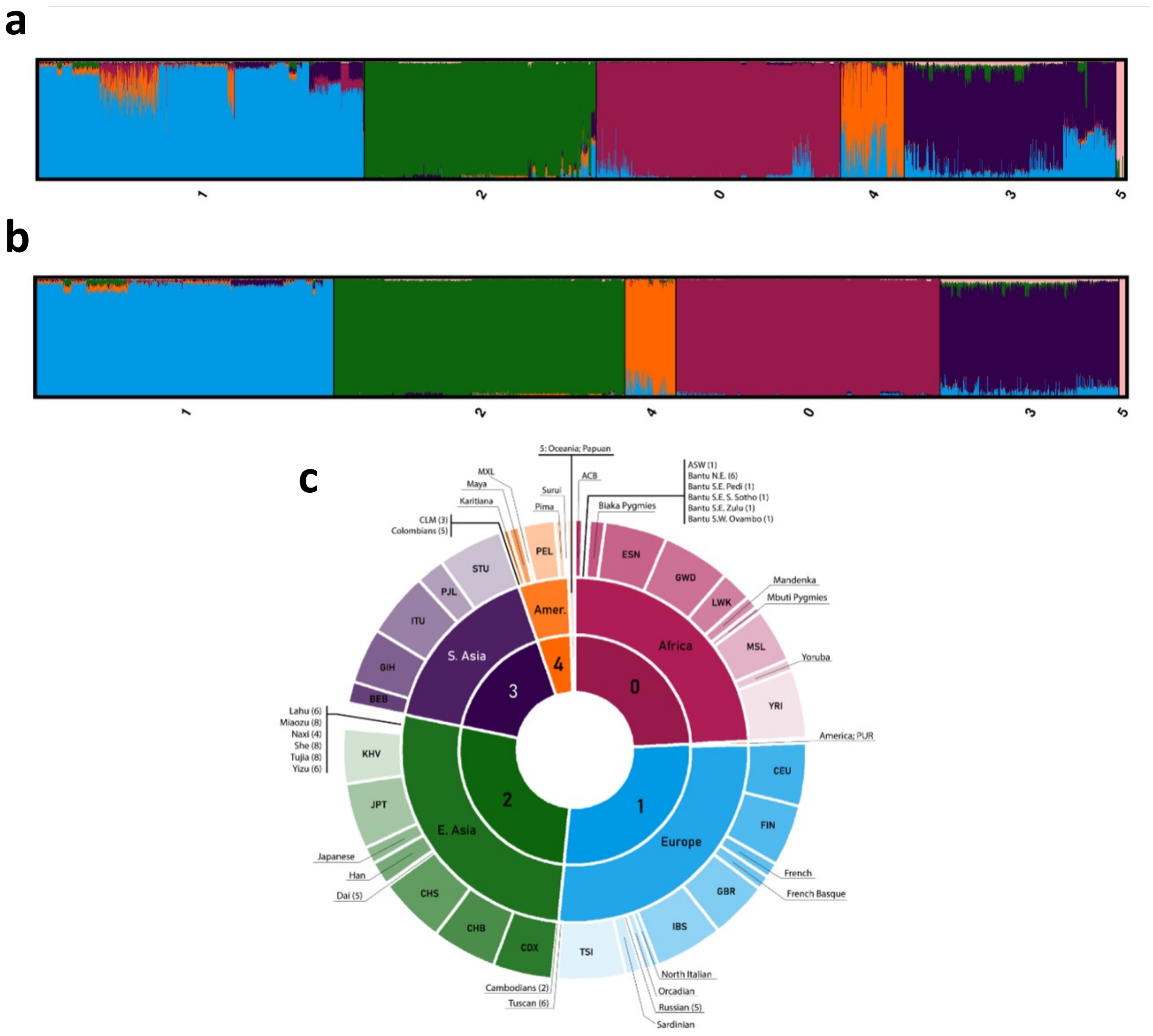

Supplementary Fig. 12 ADMIXTURE K:6 Model of 1000G and HGDP ancestry references. A, Clumpak plot of ADMIXTURE Q-file results of K:6 run for HGDP and 1000 genomes reference dataset with unsupervised K-means clustering labels applied. **B,** Clumpak plot of the same ADMIXTURE Q-file results seen in **a**, but for anchor reference data only. **C,** Population labels and the corresponding cluster number in sunburst chart to depict the population and sub-population membership for anchors in each cluster.

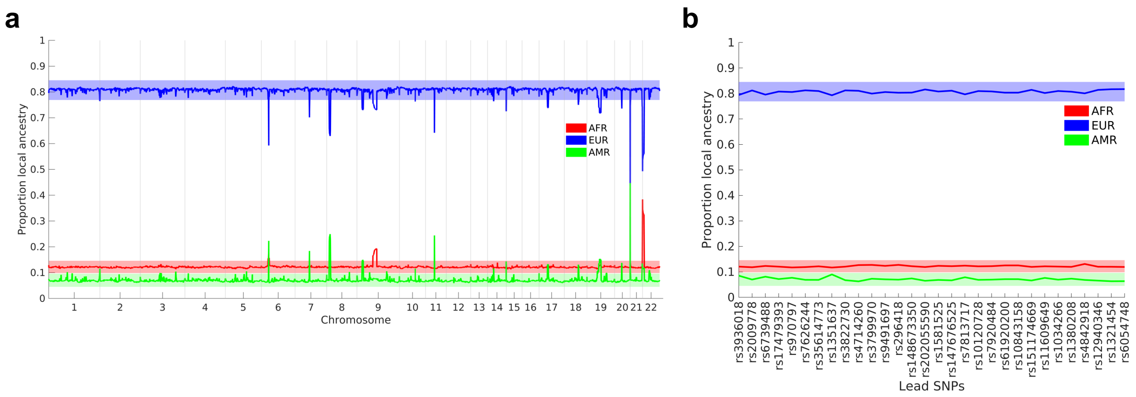

Supplementary Fig. 13 Genome-wide local ancestry proportions. a, Genome-wide fractions of local African (AFR; red), European (EUR; blue), and Indigenous American (AMR; green) ancestry inferred by RFMIX v2. **b,** local ancestry proportions at the 30 genome-wide significant lead SNPs. Bands indicate 95% confidence interval on the genome-wide local ancestry proportions.

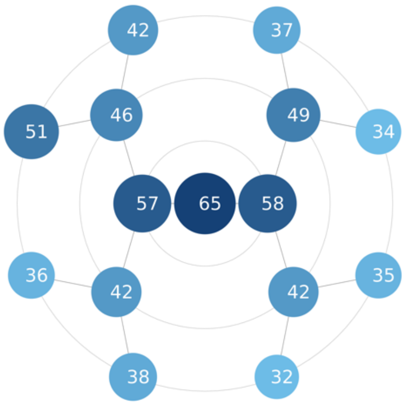

Supplementary Fig. 14 Number of phenotypic dimensions per cranial vault segment. The rosette indicates the number of PCs used to describe the major phenotypic variance in each cranial vault segment as determined by parallel analysis (Methods).

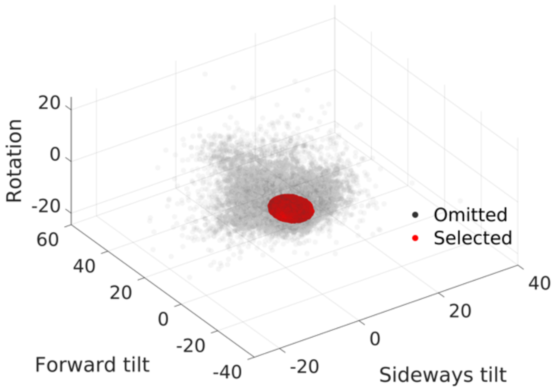

Supplementary Fig. 15 Distribution of rotations along all three dimensions. Grey dots represent omitted images while red dots, situated in the centroid, represent selected images with an aggregated score > 0.8.

Supplementary Table 1, Lead SNPs at 30 genome-wide significant GWAS loci.

P-values were obtained with CCA (upper-tail chi squared).

Support represents the number of 'suggestive' SNPs with P-value < 1e-6 that are clumped with the lead SNP.

Position is in GRCh38 coordinates.

Annotated protein coding genes include their source between brackets: Closest gene TSS (C); GREAT with default settings (GREAT); FUMA with default settings (FUMA);
eQTL co-localization based on GTEx8 and Bayesian colocalization with 'coloc' package and threshold of PP4 ≥ 0.7 (GTEX); and literature and within 500 kb (LIT).

rsID	Chromosome	Position	A1	A2	MAF	Support	Vault Segment	-log10(P discovery)	-log10(P replication)	Replication Significant	Annotated genes
rs3936018	1	119139747	T	C	0.214	236	11	18.39	0.12	FALSE	*WARS2* (C,GREAT,FUMA,GTEX), *TBX15* (FUMA,LIT), *HSD3B2* (GTEX), *HAO2* (GTEX)
rs2009778	2	65750729	C	T	0.339	69	1	19.87	12.74	TRUE	*MEIS1* (GREAT,LIT), *SPRED2* (C,GREAT)
rs6739488	2	118432725	G	A	0.472	34	1	7.92	2.65	TRUE	*INSIG2* (C,GREAT), *EN1* (GREAT,LIT)
rs17479393	2	144895720	A	T	0.210	41	1	11.73	17.96	TRUE	*ZEB2* (C,GREAT,LIT)
rs970797	2	176247091	T	G	0.426	3	5	9.85	3.21	TRUE	*MTX2* (C,GREAT), *HOXD1* (GREAT), *HOXD* cluster (LIT)
rs7626244	3	157756785	C	A	0.398	38	5	7.99	0.35	FALSE	*VEPH1* (C,GREAT), *SHOX2* (GREAT,LIT)
rs35614773	3	177593205	T	G	0.431	83	1	7.46	0.68	FALSE	*KCNMB2* (GREAT), *TBL1XR1* (C,GREAT,GTEX,LIT)
rs1351637	5	44441601	A	G	0.186	21	8	16.60	3.96	TRUE	*MRPS30* (GREAT), *FGF10* (C,GREAT,FUMA,LIT)
rs3822730	5	171747250	G	C	0.465	12	2	10.73	19.95	TRUE	*SMIM23* (C,GREAT), *FGF18* (GREAT,GTEX,LIT)
rs4714260	6	39568846	T	C	0.442	117	1	16.62	2.68	TRUE	*KCNK16* (GREAT), *KIF6* (C,GREAT,FUMA,GTEX,LIT), *DAAM2* (GTEX,LIT)
rs3799970	6	44842621	T	A	0.198	41	1	8.72	2.83	TRUE	*RUNX2* (GREAT,FUMA,LIT), *CDC5L* (C,GREAT), *SUPT3H* (FUMA)
rs9491697	6	127134977	A	G	0.418	12	3	8.64	0.11	FALSE	*RNF146* (GREAT), *RSPO3* (C,GREAT,FUMA,GTEX,LIT)
rs296418	6	133334925	A	G	0.325	137	11	32.04	6.13	TRUE	*TCF21* (GREAT), *EYA4* (C,GREAT,FUMA,GTEX,LIT)
rs148673350	7	33219926	C	T	0.294	47	1	14.16	31.99	TRUE	*BMPER* (GREAT,GTEX), *BBS9* (C,GREAT,GTEX,LIT), *RP9* (FUMA)
rs202055590	7	96585031	A	G	0.348	212	1	30.39	2.72	TRUE	*DLX6* (GREAT), *SLC25A13* (C,GREAT), *SEM1* (FUMA), *DLX5* (LIT)
rs1581525	7	121228465	A	G	0.394	68	12	8.52	0.52	FALSE	*WNT16* (C,GREAT,GTEX,LIT), *CPED1* (GREAT,FUMA)
rs147676525	7	156385384	A	G	0.019	25	15	8.84	6.04	TRUE	*SHH* (GREAT,LIT), *RNF32* (C,GREAT)
rs7813717	8	108534556	G	A	0.466	229	1	9.65	2.31	TRUE	*EMC2* (C,GREAT,FUMA,GTEX), *TMEM74* (GREAT), *EIF3E* (FUMA), *NUDCD1* (GTEX), *RSPO2* (LIT)
rs10120728	9	108615575	C	T	0.258	13	5	9.46	0.51	FALSE	*ACTL7B* (C,GREAT), *ELP1* (LIT)
rs7920484	10	93190051	A	C	0.392	54	1	9.57	1.20	FALSE	*CYP26A1* (C,GREAT), *MYOF* (GREAT), *CEP55* (LIT)
rs61920200	12	28085972	G	A	0.247	2	1	7.64	1.26	FALSE	*PTHLH* (GREAT,LIT), *CCDC91* (C,GREAT,FUMA)
rs10843158	12	28354259	C	T	0.228	563	1	10.62	11.59	TRUE	*FAR2* (GREAT), *CCDC91* (C,GREAT), *PTHLH* (LIT)
rs151174669	12	65980466	C	T	0.177	46	11	21.57	3.82	TRUE	*LLPH* (C,GREAT), *HMGA2* (GREAT,FUMA,LIT)
rs11609649	12	85182714	G	A	0.139	6	5	9.08	4.78	TRUE	*TSPAN19* (GREAT), *ALX1* (C,GREAT,FUMA,LIT), *LRRIQ1* (FUMA)
rs1034266	13	100322513	G	C	0.380	6	2	8.26	4.55	TRUE	*PCCA* (C,GREAT,FUMA), *GGACT* (GREAT), *ZIC2* (GTEX,LIT)
rs1380208	14	98408346	A	C	0.328	32	11	7.73	0.79	FALSE	*BCL11B* (C,GREAT)
rs4842918	15	83868247	T	C	0.250	43	2	10.53	0.44	FALSE	*GOLGA6L4* (GREAT,GTEX), *ADAMTSL3* (C,GREAT,FUMA,LIT), *WHAMM* (GTEX)
rs12940346	17	70079183	T	C	0.294	38	1	22.26	6.34	TRUE	*KCNJ16* (C,GREAT,FUMA,GTEX), *MAP2K6* (GREAT), *KCNJ2* (LIT)
rs1321454	20	6608452	T	C	0.390	149	3	12.07	1.88	TRUE	*FERMT1* (GREAT), *BMP2* (C,GREAT,LIT)
rs6054748	20	7123941	G	A	0.383	27	4	9.88	2.07	TRUE	*BMP2* (C,GREAT,LIT), *HAO1* (GREAT)

Supplementary Table 2, GREAT functional enrichment of genome-wide loci for mouse phenotypes

Terms were considered significant if both binomial P-values (one-tailed) and hypergemetric P-values (one-tailed) were significant at 5% FDR (BinomFdrQ, and HyperFdrQ). Only the top 100 terms are shown.

ID	Description	BinomP	BinomFdrQ	HyperP	HyperBonfP	HyperFdrQ	ObsGenes	TotalGenes	Genes
MP:0003935	abnormal craniofacial development	5.103249e-11	4.816446e-7	3.862053e-12	3.645006e-8	3.645006e-8	14	394	*ALX1,BCL11B,BMP2,BMPER,CYP26A1,DLX6,FGF10,FGF18,PTHLH,RUNX2,SHH,SHOX2,TCF21,ZEB2*
MP:0011504	abnormal limb long bone morphology	8.993431e-10	4.244000e-6	5.032908e-10	4.750059e-6	1.583353e-6	13	466	*ALX1,BMP2,BMPER,DLX6,EN1,FGF10,FGF18,PTHLH,RSPO3,RUNX2,SHH,SHOX2,SPRED2*
MP:0000550	abnormal forelimb morphology	1.716180e-9	5.399102e-6	3.850646e-10	3.634240e-6	1.817120e-6	10	210	*BMP2,BMPER,EN1,FGF10,FGF18,PTHLH,RSPO3,RUNX2,SHH,SHOX2*
MP:0002115	abnormal limb bone morphology	2.174117e-9	5.129829e-6	8.610132e-10	8.126243e-6	2.031561e-6	13	487	*ALX1,BMP2,BMPER,DLX6,EN1,FGF10,FGF18,PTHLH,RSPO3,RUNX2,SHH,SHOX2,SPRED2*
MP:0000556	abnormal hindlimb morphology	2.732589e-9	5.158035e-6	2.077179e-9	1.960442e-5	2.450552e-6	12	422	*ALX1,BMP2,CYP26A1,DLX6,FGF10,FGF18,PTHLH,RSPO3,RUNX2,SHH,SHOX2,SPRED2*
MP:0005296	abnormal humerus morphology	4.978875e-9	7.831770e-6	1.273141e-9	1.201590e-5	2.002651e-6	8	115	*BMP2,BMPER,FGF10,PTHLH,RSPO3,RUNX2,SHH,SHOX2*
MP:0000149	abnormal scapula morphology	5.281340e-9	7.120755e-6	1.022471e-9	9.650081e-6	1.930016e-6	7	69	*ALX1,BMP2,BMPER,FGF10,RSPO3,RUNX2,SHH*
MP:0003854	abnormal forelimb stylopod morphology	7.621308e-9	8.991238e-6	1.788959e-9	1.688420e-5	2.412028e-6	8	120	*BMP2,BMPER,FGF10,PTHLH,RSPO3,RUNX2,SHH,SHOX2*
MP:0002109	abnormal limb morphology	8.630634e-9	9.050658e-6	7.794442e-9	7.356394e-5	7.356394e-6	15	834	*ALX1,BMP2,BMPER,CYP26A1,DLX6,EN1,FGF10,FGF18,HMGA2,PTHLH,RSPO3,RUNX2,SHH,SHOX2,SPRED2*
MP:0003723	abnormal long bone morphology	1.773225e-8	1.673570e-5	1.101133e-8	1.039249e-4	8.660411e-6	13	602	*ALX1,BMP2,BMPER,DLX6,EN1,FGF10,FGF18,PTHLH,RSPO3,RUNX2,SHH,SHOX2,SPRED2*
MP:0004423	abnormal squamosal bone morphology	3.329321e-8	2.856557e-5	5.732391e-8	5.410231e-4	2.705115e-5	5	36	*ALX1,BMP2,BMPER,DLX6,SHH*
MP:0000077	abnormal interparietal bone morphology	3.876218e-8	3.048645e-5	9.870380e-9	9.315665e-5	8.468786e-6	6	54	*ALX1,BMP2,BMPER,DLX6,RUNX2,SHH*
MP:0001218	thin epidermis	5.433340e-8	3.944605e-5	6.126494e-9	5.782185e-5	6.424650e-6	6	50	*BCL11B,FERMT1,FGF10,PTHLH,RUNX2,SHH*
MP:0003857	abnormal hindlimb zeugopod morphology	6.568578e-8	4.428160e-5	5.734328e-8	5.412059e-4	2.577171e-5	9	264	*ALX1,BMP2,FGF18,PTHLH,RSPO3,RUNX2,SHH,SHOX2,SPRED2*
MP:0004354	absent deltoid tuberosity	1.416544e-7	8.912895e-5	2.292820e-7	2.163964e-3	7.728441e-5	4	19	*BMP2,BMPER,RUNX2,SHH*
MP:0009250	abnormal appendicular skeleton morphology	2.224653e-7	1.312267e-4	1.380748e-7	1.303150e-3	5.429792e-5	13	747	*ALX1,BMP2,BMPER,DLX6,EN1,FGF10,FGF18,PTHLH,RSPO3,RUNX2,SHH,SHOX2,SPRED2*
MP:0004508	abnormal pectoral girdle bone morphology	2.648398e-7	1.470328e-4	7.402730e-8	6.986697e-4	3.175771e-5	9	272	*ALX1,BMP2,BMPER,EN1,FGF10,PTHLH,RSPO3,RUNX2,SHH*
MP:0000074	abnormal neurocranium morphology	3.945067e-7	2.068530e-4	8.918145e-8	8.416945e-4	3.659541e-5	9	278	*ALX1,BMP2,BMPER,DLX6,FGF18,HOXD1,PTHLH,RUNX2,SHH*
MP:0000164	abnormal cartilage development	5.526388e-7	2.745161e-4	1.778782e-7	1.678814e-3	6.456979e-5	8	216	*BMP2,BMPER,FGF18,PTHLH,RUNX2,SHH,SHOX2,SPRED2*
MP:0002932	abnormal joint morphology	7.286214e-7	3.438364e-4	5.132499e-7	4.844053e-3	1.513766e-4	9	342	*ALX1,BMP2,BMPER,EN1,FGF18,HOXD1,RUNX2,SHH,SHOX2*
MP:0003743	abnormal facial morphology	7.769004e-7	3.491612e-4	2.163370e-8	2.041789e-4	1.458420e-5	14	764	*ALX1,BCL11B,BMP2,CYP26A1,DLX6,EYA4,FGF10,FGF18,PTHLH,RUNX2,SHH,SHOX2,TCF21,ZEB2*
MP:0000163	abnormal cartilage morphology	8.256614e-7	3.542087e-4	1.784690e-7	1.684390e-3	6.238483e-5	10	400	*ALX1,BMP2,BMPER,DLX6,FGF18,PTHLH,RUNX2,SHH,SHOX2,SPRED2*
MP:0000432	abnormal head morphology	8.866592e-7	3.638387e-4	3.429503e-8	3.236765e-4	2.022978e-5	15	932	*ALX1,BCL11B,BMP2,CYP26A1,DLX6,EYA4,FGF10,FGF18,HMGA2,PTHLH,RUNX2,SHH,SHOX2,TCF21,ZEB2*
MP:0010976	small lung lobe	1.095477e-6	4.307963e-4	1.985672e-8	1.874077e-4	1.441598e-5	4	11	*FGF10,FGF18,SHH,TCF21*
MP:0004353	abnormal deltoid tuberosity morphology	1.622243e-6	6.124292e-4	2.349447e-6	2.217408e-2	5.279543e-4	4	33	*BMP2,BMPER,RUNX2,SHH*
MP:0000455	abnormal maxilla morphology	1.768282e-6	6.418864e-4	4.457087e-7	4.206599e-3	1.402200e-4	7	165	*ALX1,BCL11B,BMP2,DLX6,FGF18,PTHLH,SHH*
MP:0000452	abnormal mouth morphology	2.037929e-6	7.123694e-4	5.107818e-8	4.820759e-4	2.678199e-5	12	563	*ALX1,BCL11B,BMP2,DLX6,EYA4,FGF10,FGF18,PTHLH,RUNX2,SHH,SHOX2,TCF21*
MP:0006402	small molars	2.208256e-6	7.443400e-4	4.283981e-8	4.043221e-4	2.378365e-5	4	13	*BCL11B,FGF10,RUNX2,SHH*
MP:0005272	abnormal temporal bone morphology	2.710534e-6	8.821386e-4	1.090828e-6	1.029523e-2	2.859787e-4	5	64	*ALX1,BMP2,BMPER,DLX6,SHH*
MP:0000572	abnormal autopod morphology	2.723942e-6	8.569522e-4	1.805355e-6	1.703894e-2	4.155839e-4	9	398	*ALX1,BMP2,DLX6,EN1,FGF10,HMGA2,RSPO3,RUNX2,SHH*
MP:0003855	abnormal forelimb zeugopod morphology	3.230920e-6	9.836588e-4	1.267997e-6	1.196736e-2	3.068553e-4	6	121	*BMP2,FGF10,FGF18,PTHLH,RUNX2,SHH*
MP:0003755	abnormal palate morphology	3.301515e-6	9.737406e-4	3.132150e-8	2.956123e-4	1.970749e-5	10	332	*ALX1,BMP2,DLX6,EYA4,FGF10,FGF18,RUNX2,SHH,SHOX2,TCF21*
MP:0000914	exencephaly	3.323831e-6	9.506157e-4	1.010556e-5	9.537628e-2	1.467327e-3	7	264	*ALX1,BMP2,BMPER,CYP26A1,DLX6,SHH,ZEB2*
MP:0005306	abnormal phalanx morphology	4.122960e-6	1.144485e-3	5.624185e-6	5.308106e-2	1.020790e-3	5	89	*BMP2,DLX6,EN1,RUNX2,SHH*
MP:0000558	abnormal tibia morphology	4.291680e-6	1.157282e-3	4.645675e-7	4.384588e-3	1.414383e-4	8	245	*ALX1,FGF18,PTHLH,RSPO3,RUNX2,SHH,SHOX2,SPRED2*
MP:0002110	abnormal digit morphology	5.893533e-6	1.545088e-3	4.914390e-6	4.638201e-2	9.465717e-4	8	336	*ALX1,BMP2,DLX6,EN1,FGF10,RSPO3,RUNX2,SHH*
MP:0004686	decreased length of long bones	6.667657e-6	1.700793e-3	5.717252e-6	5.395942e-2	9.992486e-4	8	343	*ALX1,BMP2,FGF18,PTHLH,RSPO3,RUNX2,SHH,SPRED2*
MP:0008395	abnormal osteoblast differentiation	1.087818e-5	2.701796e-3	4.093558e-5	3.863500e-1	4.711585e-3	4	67	*BMP2,FGF18,PTHLH,RUNX2*
MP:0000111	cleft palate	1.152304e-5	2.788576e-3	1.703660e-7	1.607914e-3	6.431657e-5	9	300	*ALX1,BMP2,EYA4,FGF10,FGF18,RUNX2,SHH,SHOX2,TCF21*
MP:0002689	abnormal molar morphology	1.191476e-5	2.811288e-3	3.457175e-7	3.262882e-3	1.125132e-4	5	51	*BCL11B,FGF10,PTHLH,RUNX2,SHH*
MP:0000757	herniated abdominal wall	1.218923e-5	2.805901e-3	6.262606e-5	5.910648e-1	6.287923e-3	3	27	*ALX1,BMP2,CYP26A1*
MP:0011711	impaired osteoblast differentiation	1.485919e-5	3.339072e-3	5.259747e-4	1.000000e+0	2.954851e-2	2	12	*BMP2,RUNX2*
MP:0006279	abnormal limb development	1.663221e-5	3.650577e-3	9.713404e-6	9.167511e-2	1.432424e-3	6	172	*BMP2,CYP26A1,EN1,FGF10,RUNX2,SHH*
MP:0000454	abnormal jaw morphology	1.759326e-5	3.773754e-3	5.979228e-6	5.643195e-2	1.007713e-3	9	461	*ALX1,BCL11B,BMP2,DLX6,FGF10,FGF18,PTHLH,RUNX2,SHH*
MP:0030088	microdontia	1.854969e-5	3.890488e-3	1.188174e-6	1.121399e-2	2.951049e-4	4	28	*BCL11B,FGF10,RUNX2,SHH*
MP:0011087	neonatal lethality, complete penetrance	1.886106e-5	3.869797e-3	5.401320e-8	5.097766e-4	2.683035e-5	13	689	*ALX1,BCL11B,BMPER,DLX6,EN1,EYA4,FGF10,FGF18,PTHLH,RSPO3,RUNX2,SHH,TCF21*
MP:0000438	abnormal cranium morphology	2.047592e-5	4.111739e-3	5.683199e-6	5.363803e-2	1.012038e-3	11	727	*ALX1,BCL11B,BMP2,BMPER,DLX6,FGF10,FGF18,HOXD1,PTHLH,RUNX2,SHH*
MP:0004384	small interparietal bone	2.184758e-5	4.295780e-3	4.360050e-5	4.115015e-1	4.898828e-3	3	24	*BMP2,BMPER,RUNX2*
MP:0000104	abnormal sphenoid bone morphology	2.311927e-5	4.453054e-3	9.482794e-6	8.949861e-2	1.420613e-3	5	99	*ALX1,BMP2,BMPER,DLX6,RUNX2*
MP:0002116	abnormal craniofacial bone morphology	2.463034e-5	4.649223e-3	6.899283e-6	6.511543e-2	1.122680e-3	11	742	*ALX1,BCL11B,BMP2,BMPER,DLX6,FGF10,FGF18,HOXD1,PTHLH,RUNX2,SHH*
MP:0000150	abnormal rib morphology	2.668000e-5	4.937369e-3	1.074575e-5	1.014184e-1	1.513707e-3	8	374	*ALX1,BMPER,CYP26A1,EN1,FGF18,PTHLH,RUNX2,SHH*
MP:0003235	abnormal alisphenoid bone morphology	2.811527e-5	5.102922e-3	1.153991e-4	1.000000e+0	9.992080e-3	3	33	*ALX1,BMP2,DLX6*
MP:0010825	abnormal lung saccule morphology	3.573915e-5	6.364266e-3	5.169562e-6	4.879033e-2	9.758065e-4	4	40	*BMPER,FGF18,PTHLH,SHH*
MP:0003072	abnormal metatarsal bone morphology	3.582258e-5	6.260991e-3	5.169562e-6	4.879033e-2	9.758065e-4	4	40	*DLX6,FGF18,RUNX2,SHH*
MP:0009655	abnormal secondary palate development	3.592077e-5	6.164004e-3	1.149900e-6	1.085276e-2	2.933177e-4	6	119	*FGF10,FGF18,RUNX2,SHH,SHOX2,TCF21*
MP:0000564	syndactyly	3.624873e-5	6.109206e-3	3.053462e-5	2.881857e-1	3.742672e-3	5	126	*BMP2,DLX6,EN1,FGF10,SHH*
MP:0000168	abnormal bone marrow development	3.657764e-5	6.056487e-3	8.321611e-4	1.000000e+0	4.090592e-2	2	15	*BMP2,PTHLH*
MP:0004506	abnormal pubis morphology	3.669374e-5	5.970957e-3	6.219105e-7	5.869591e-3	1.778664e-4	4	24	*ALX1,BMPER,RUNX2,SHH*
MP:0004692	small pubis	3.729513e-5	5.965957e-3	7.805296e-7	7.366638e-3	2.166658e-4	3	7	*ALX1,BMPER,RUNX2*
MP:0000102	abnormal nasal bone morphology	3.784421e-5	5.952894e-3	6.722135e-5	6.344351e-1	6.608699e-3	4	76	*BMP2,DLX6,RUNX2,SHH*
MP:0009653	abnormal palate development	4.361988e-5	6.748925e-3	1.395833e-6	1.317387e-2	3.293468e-4	6	123	*FGF10,FGF18,RUNX2,SHH,SHOX2,TCF21*
MP:0005298	abnormal clavicle morphology	4.417022e-5	6.723847e-3	4.662524e-6	4.400490e-2	9.778867e-4	4	39	*ALX1,PTHLH,RUNX2,SHH*
MP:0000085	large anterior fontanelle	4.630863e-5	6.937474e-3	2.884879e-4	1.000000e+0	2.047180e-2	2	9	*BMP2,RUNX2*
MP:0005274	abnormal viscerocranium morphology	4.641000e-5	6.844025e-3	1.665874e-5	1.572252e-1	2.214439e-3	9	524	*ALX1,BCL11B,BMP2,DLX6,FGF10,FGF18,PTHLH,RUNX2,SHH*
MP:0005269	abnormal occipital bone morphology	4.642900e-5	6.741491e-3	6.981006e-6	6.588673e-2	1.116724e-3	5	93	*ALX1,BMPER,HOXD1,RUNX2,SHH*
MP:0008272	abnormal endochondral bone ossification	4.712333e-5	6.738636e-3	4.560339e-5	4.304048e-1	4.947182e-3	5	137	*BMP2,EN1,FGF18,PTHLH,RUNX2*
MP:0004989	decreased osteoblast cell number	5.838923e-5	8.225038e-3	3.772168e-4	1.000000e+0	2.326910e-2	3	49	*BMP2,PTHLH,RUNX2*
MP:0004468	small zygomatic bone	5.931901e-5	8.233130e-3	3.599450e-4	1.000000e+0	2.249775e-2	2	10	*ALX1,BMP2*
MP:0012270	cardiac edema	6.002846e-5	8.210849e-3	7.225373e-4	1.000000e+0	3.788504e-2	2	14	*BMP2,SHH*
MP:0005270	abnormal zygomatic bone morphology	6.108285e-5	8.235713e-3	1.909541e-4	1.000000e+0	1.465223e-2	3	39	*ALX1,BMP2,DLX6*
MP:0004509	abnormal pelvic girdle bone morphology	6.280805e-5	8.349048e-3	2.929749e-6	2.765097e-2	6.430458e-4	5	78	*ALX1,BMPER,FGF10,RUNX2,SHH*
MP:0000929	open neural tube	6.479435e-5	8.493459e-3	8.474496e-5	7.998229e-1	7.841401e-3	6	253	*ALX1,BMP2,CYP26A1,DLX6,SHH,ZEB2*
MP:0002764	short tibia	7.111284e-5	9.194013e-3	1.072097e-5	1.011845e-1	1.533099e-3	6	175	*ALX1,FGF18,PTHLH,RSPO3,SHH,SPRED2*
MP:0010975	abnormal lung lobe morphology	7.173039e-5	9.148533e-3	4.662524e-6	4.400490e-2	9.778867e-4	4	39	*FGF10,FGF18,SHH,TCF21*
MP:0001302	eyelids open at birth	8.298017e-5	1.044222e-2	5.942086e-6	5.608141e-2	1.019662e-3	5	90	*ALX1,BCL11B,FGF10,RUNX2,SHH*
MP:0010029	abnormal basicranium morphology	1.148670e-4	1.426467e-2	7.357185e-6	6.943711e-2	1.157285e-3	5	94	*ALX1,BCL11B,BMPER,DLX6,RUNX2*
MP:0011143	thick lung-associated mesenchyme	1.227745e-4	1.504865e-2	4.360050e-5	4.115015e-1	4.898828e-3	3	24	*BMPER,FGF18,PTHLH*
MP:0011028	impaired branching involved in bronchus morphogenesis	1.247955e-4	1.510026e-2	4.856438e-6	4.583506e-2	9.548971e-4	3	12	*FGF10,SHH,TCF21*
MP:0001176	abnormal lung development	1.261733e-4	1.507372e-2	7.928049e-6	7.482493e-2	1.226638e-3	6	166	*BMPER,FGF10,FGF18,PTHLH,SHH,TCF21*
MP:0002818	abnormal dentin morphology	1.314201e-4	1.550429e-2	4.360050e-5	4.115015e-1	4.898828e-3	3	24	*PTHLH,RUNX2,SHH*
MP:0002950	abnormal neural crest cell migration	1.359183e-4	1.583700e-2	1.380863e-5	1.303258e-1	1.916557e-3	4	51	*CYP26A1,FGF10,SHH,ZEB2*
MP:0012089	decreased midbrain size	1.416124e-4	1.629924e-2	4.944527e-5	4.666645e-1	5.017897e-3	3	25	*CYP26A1,EN1,SHH*
MP:0000552	abnormal radius morphology	1.431815e-4	1.628129e-2	8.157072e-6	7.698645e-2	1.241717e-3	5	96	*FGF10,FGF18,PTHLH,RUNX2,SHH*
MP:0003856	abnormal hindlimb stylopod morphology	1.467523e-4	1.648867e-2	3.093910e-4	1.000000e+0	2.041981e-2	5	206	*BMP2,PTHLH,RSPO3,RUNX2,SHOX2*
MP:0014139	abnormal ectoderm morphology	1.533814e-4	1.703075e-2	2.928989e-4	1.000000e+0	1.946746e-2	3	45	*BMP2,EN1,FGF10*
MP:0002113	abnormal skeleton development	1.536255e-4	1.685951e-2	1.026368e-4	9.686861e-1	9.053141e-3	8	514	*BMP2,BMPER,FGF18,PTHLH,RUNX2,SHH,SHOX2,SPRED2*
MP:0011024	abnormal branching involved in lung morphogenesis	1.554211e-4	1.686051e-2	4.662524e-6	4.400490e-2	9.778867e-4	4	39	*BMPER,FGF10,SHH,TCF21*
MP:0004596	abnormal mandibular angle morphology	1.578581e-4	1.693028e-2	6.204609e-4	1.000000e+0	3.346234e-2	2	13	*BMP2,DLX6*
MP:0002884	abnormal pharyngeal arch morphology	1.668432e-4	1.769288e-2	1.338484e-4	1.000000e+0	1.108124e-2	5	172	*BMP2,CYP26A1,DLX6,FGF10,ZEB2*
MP:0005358	abnormal incisor morphology	1.735454e-4	1.819913e-2	1.385179e-5	1.307332e-1	1.894684e-3	5	107	*BCL11B,FGF10,PTHLH,RUNX2,SHH*
MP:0000030	abnormal tympanic ring morphology	1.742930e-4	1.807667e-2	4.004805e-4	1.000000e+0	2.422907e-2	3	50	*BMP2,BMPER,DLX6*
MP:0004358	bowed tibia	2.102485e-4	2.156875e-2	2.475840e-5	2.336698e-1	3.157700e-3	3	20	*FGF18,SHH,SHOX2*
MP:0002111	abnormal tail morphology	2.104262e-4	2.135487e-2	2.710991e-4	1.000000e+0	1.983437e-2	7	445	*BMP2,BMPER,CYP26A1,FGF10,FGF18,HMGA2,SHH*
MP:0000260	abnormal angiogenesis	2.196716e-4	2.205596e-2	1.770626e-4	1.000000e+0	1.369768e-2	7	415	*BMP2,FGF10,MEIS1,PTHLH,RSPO3,RUNX2,SHH*
MP:0004352	absent humerus	2.223096e-4	2.208587e-2	4.852734e-5	4.580010e-1	5.204557e-3	2	4	*FGF10,SHOX2*
MP:0009546	absent gastric milk in neonates	2.240007e-4	2.202207e-2	2.136206e-5	2.016151e-1	2.800210e-3	5	117	*ALX1,BCL11B,EN1,PCCA,RSPO3*
MP:0002450	abnormal lymph organ development	2.318359e-4	2.255739e-2	2.924919e-3	1.000000e+0	1.026223e-1	2	28	*BMP2,PTHLH*
MP:0009890	cleft secondary palate	2.352368e-4	2.265474e-2	6.191590e-6	5.843623e-2	1.025197e-3	6	159	*FGF10,FGF18,RUNX2,SHH,SHOX2,TCF21*
MP:0003641	small lung	2.586417e-4	2.465718e-2	2.614298e-5	2.467374e-1	3.289833e-3	5	122	*FGF10,FGF18,PTHLH,SHH,TCF21*
MP:0003419	delayed endochondral bone ossification	2.658525e-4	2.509116e-2	7.541740e-4	1.000000e+0	3.889560e-2	3	62	*BMP2,FGF18,RUNX2*

Supplementary Table 3, GREAT functional enrichment of genome-wide loci for GO biological processes

Terms were considered significant if both binomial P-values (one-tailed) and hypergemetric P-values (one-tailed) were significant at 5% FDR (BinomFdrQ, and HyperFdrQ). Only the top 100 terms are shown.

ID	Description	BinomP	BinomFdrQ	HyperP	HyperFdrQ	ObsGenes	TotalGenes	Genes
GO:0001501	skeletal system development	9.799348e-9	1.265782e-4	4.651235e-10	6.008000e-6	13	463	*ALX1,BMP2,DLX6,EN1,FGF18,HMGA2,HOXD1,INSIG2,MEIS1,PTHLH,RUNX2,SHH,SHOX2*
GO:0048762	mesenchymal cell differentiation	3.330145e-8	2.150774e-4	3.383185e-9	2.185030e-5	8	130	*ALX1,BMP2,FGF10,HMGA2,SHH,TCF21,WNT16,ZEB2*
GO:0009790	embryo development	2.502524e-7	1.077503e-3	2.322772e-8	7.500811e-5	15	905	*ALX1,BMP2,DLX6,EN1,FGF10,HMGA2,HOXD1,INSIG2,RSPO3,RUNX2,SHH,SHOX2,TCF21,WNT16,ZEB2*
GO:0060485	mesenchyme development	4.088109e-7	1.320153e-3	6.362227e-8	1.369681e-4	8	189	*ALX1,BMP2,FGF10,HMGA2,SHH,TCF21,WNT16,ZEB2*
GO:0048598	embryonic morphogenesis	9.313046e-7	2.405932e-3	3.743503e-9	1.611828e-5	13	550	*ALX1,DLX6,EN1,FGF10,HMGA2,INSIG2,RSPO3,RUNX2,SHH,SHOX2,TCF21,WNT16,ZEB2*
GO:0042475	odontogenesis of dentin-containing tooth	1.202830e-6	2.589493e-3	2.575072e-6	1.750642e-3	5	76	*BCL11B,BMP2,FGF10,RUNX2,SHH*
GO:0061448	connective tissue development	1.869446e-6	3.449662e-3	1.418501e-6	1.221518e-3	7	196	*ALX1,BMP2,FGF18,HMGA2,RUNX2,SHOX2,TBL1XR1*
GO:0048562	embryonic organ morphogenesis	1.961521e-6	3.167121e-3	9.772534e-8	1.803312e-4	9	281	*ALX1,DLX6,FGF10,INSIG2,RUNX2,SHH,SHOX2,TCF21,WNT16*
GO:0061035	regulation of cartilage development	2.061750e-6	2.959069e-3	9.298337e-7	9.238971e-4	5	62	*BMP2,FGF18,PTHLH,RUNX2,SHOX2*
GO:0002062	chondrocyte differentiation	2.091540e-6	2.701642e-3	9.298337e-7	9.238971e-4	5	62	*BMP2,FGF18,HMGA2,RUNX2,SHOX2*
GO:0009887	animal organ morphogenesis	2.626751e-6	3.084522e-3	6.718819e-7	7.232249e-4	13	858	*ALX1,BCL11B,BMP2,DLX6,FGF10,FGF18,INSIG2,MEIS1,RUNX2,SHH,SHOX2,TCF21,WNT16*
GO:0048646	anatomical structure formation involved in morphogenesis	3.602997e-6	3.878326e-3	4.076686e-7	5.265855e-4	13	821	*ALX1,BMPER,DLX6,FGF10,FGF18,HMGA2,MEIS1,MYOF,RSPO3,SHH,TCF21,WNT16,ZEB2*
GO:0048568	embryonic organ development	3.769832e-6	3.745763e-3	2.363122e-8	6.104889e-5	11	418	*ALX1,DLX6,EN1,FGF10,INSIG2,RSPO3,RUNX2,SHH,SHOX2,TCF21,WNT16*
GO:0051216	cartilage development	4.400594e-6	4.060177e-3	4.426127e-6	2.485751e-3	6	150	*ALX1,BMP2,FGF18,HMGA2,RUNX2,SHOX2*
GO:0048729	tissue morphogenesis	4.497534e-6	3.872976e-3	1.637107e-6	1.321657e-3	10	510	*ALX1,BMP2,FGF10,HMGA2,RSPO3,SHH,SHOX2,TCF21,WNT16,ZEB2*
GO:0001649	osteoblast differentiation	5.027256e-6	4.058567e-3	2.512608e-5	7.727466e-3	5	121	*BMP2,PTHLH,RUNX2,SHH,SHOX2*
GO:0060348	bone development	6.398060e-6	4.861397e-3	7.928049e-6	3.531262e-3	6	166	*BMP2,FGF18,INSIG2,MEIS1,RUNX2,SHOX2*
GO:0042476	odontogenesis	8.029757e-6	5.762243e-3	1.449267e-5	5.200051e-3	5	108	*BCL11B,BMP2,FGF10,RUNX2,SHH*
GO:0042481	regulation of odontogenesis	9.759835e-6	6.635147e-3	5.578101e-5	1.334302e-2	3	26	*BMP2,RUNX2,SHH*
GO:0035295	tube development	1.098422e-5	7.094158e-3	3.012946e-6	1.853249e-3	10	546	*ALX1,BMP2,BMPER,EN1,FGF10,FGF18,SHH,SHOX2,TCF21,ZEB2*
GO:0001823	mesonephros development	1.312862e-5	8.075352e-3	5.942086e-6	2.842738e-3	5	90	*BMP2,BMPER,FGF10,SHH,TCF21*
GO:0048557	embryonic digestive tract morphogenesis	1.479888e-5	8.688961e-3	2.292820e-7	3.290706e-4	4	19	*FGF10,SHH,SHOX2,TCF21*
GO:0030177	positive regulation of Wnt signaling pathway	1.570510e-5	8.820121e-3	5.549050e-6	2.756811e-3	6	156	*BMP2,FGF10,RNF146,RSPO3,SHH,ZEB2*
GO:0035108	limb morphogenesis	1.681807e-5	9.051625e-3	2.032810e-7	3.282226e-4	7	147	*ALX1,DLX6,EN1,FGF10,RUNX2,SHH,SHOX2*
GO:0043009	chordate embryonic development	2.098877e-5	1.084448e-2	2.621356e-5	7.524457e-3	9	555	*ALX1,BMP2,EN1,HOXD1,RSPO3,RUNX2,SHH,SHOX2,ZEB2*
GO:0009792	embryo development ending in birth or egg hatching	2.203482e-5	1.094707e-2	2.812437e-5	7.897445e-3	9	560	*ALX1,BMP2,EN1,HOXD1,RSPO3,RUNX2,SHH,SHOX2,ZEB2*
GO:0048839	inner ear development	2.412045e-5	1.153940e-2	1.219446e-5	4.773207e-3	6	179	*BMP2,BMPER,DLX6,FGF10,INSIG2,SHH*
GO:0060173	limb development	2.675300e-5	1.234173e-2	5.901900e-7	6.930440e-4	7	172	*ALX1,DLX6,EN1,FGF10,RUNX2,SHH,SHOX2*
GO:0001503	ossification	2.954978e-5	1.316188e-2	7.762052e-5	1.591467e-2	6	249	*BMP2,FGF18,PTHLH,RUNX2,SHH,SHOX2*
GO:0035115	embryonic forelimb morphogenesis	3.062717e-5	1.318704e-2	2.069069e-6	1.484787e-3	4	32	*EN1,RUNX2,SHH,SHOX2*
GO:0043616	keratinocyte proliferation	3.163076e-5	1.317982e-2	4.856438e-6	2.613775e-3	3	12	*FERMT1,FGF10,WNT16*
GO:0072359	circulatory system development	3.299543e-5	1.331881e-2	1.275254e-5	4.844840e-3	11	792	*BMP2,BMPER,FGF10,FGF18,MEIS1,RSPO3,SHH,SHOX2,TCF21,WARS2,WNT16*
GO:0007423	sensory organ development	3.358539e-5	1.314614e-2	1.407723e-5	5.195302e-3	9	513	*BCL11B,BMP2,BMPER,DLX6,FGF10,INSIG2,MEIS1,SHH,WNT16*
GO:0002009	morphogenesis of an epithelium	3.773384e-5	1.433553e-2	1.943343e-5	6.436452e-3	8	406	*ALX1,BMP2,FGF10,RSPO3,SHH,TCF21,WNT16,ZEB2*
GO:0030326	embryonic limb morphogenesis	4.278237e-5	1.578914e-2	1.683187e-6	1.278925e-3	6	127	*ALX1,DLX6,EN1,RUNX2,SHH,SHOX2*
GO:0061036	positive regulation of cartilage development	4.413986e-5	1.583763e-2	7.791668e-5	1.572578e-2	3	29	*BMP2,FGF18,RUNX2*
GO:0050673	epithelial cell proliferation	4.603547e-5	1.607136e-2	3.985753e-6	2.340181e-3	5	83	*BMPER,FERMT1,FGF10,SHH,WNT16*
GO:0042487	regulation of odontogenesis of dentin-containing tooth	4.874129e-5	1.656819e-2	7.225373e-4	8.186855e-2	2	14	*BMP2,RUNX2*
GO:0043583	ear development	5.471523e-5	1.812196e-2	2.555976e-5	7.678033e-3	6	204	*BMP2,BMPER,DLX6,FGF10,INSIG2,SHH*
GO:0035136	forelimb morphogenesis	6.706292e-5	2.165629e-2	5.169562e-6	2.671009e-3	4	40	*EN1,RUNX2,SHH,SHOX2*
GO:0072111	cell proliferation involved in kidney development	7.562083e-5	2.382425e-2	3.599450e-4	5.406290e-2	2	10	*BMP2,SHH*
GO:1901862	negative regulation of muscle tissue development	8.489088e-5	2.610799e-2	2.928989e-4	4.670833e-2	3	45	*BMP2,MEIS1,SHH*
GO:0009952	anterior/posterior pattern specification	8.805062e-5	2.645000e-2	2.459117e-4	4.179528e-2	5	196	*ALX1,BMP2,EN1,SHH,ZEB2*
GO:0021978	telencephalon regionalization	9.423318e-5	2.766386e-2	6.204609e-4	7.706244e-2	2	13	*BMP2,SHH*
GO:0035239	tube morphogenesis	9.666666e-5	2.774763e-2	3.844390e-5	1.013428e-2	7	325	*ALX1,BMP2,FGF10,SHH,SHOX2,TCF21,ZEB2*
GO:0048566	embryonic digestive tract development	9.810631e-5	2.754868e-2	2.993501e-6	1.933353e-3	4	35	*FGF10,SHH,SHOX2,TCF21*
GO:0001525	angiogenesis	1.075675e-4	2.956275e-2	1.939250e-5	6.591919e-3	7	292	*BMPER,FGF10,FGF18,MEIS1,RSPO3,SHH,TCF21*
GO:0002076	osteoblast development	1.078027e-4	2.901016e-2	1.485867e-5	5.187282e-3	3	17	*PTHLH,RUNX2,SHH*
GO:0060021	palate development	1.086550e-4	2.864279e-2	6.620061e-6	3.053976e-3	5	92	*ALX1,DLX6,INSIG2,SHH,TCF21*
GO:0021536	diencephalon development	1.102575e-4	2.848392e-2	7.075414e-5	1.523219e-2	4	77	*BMP2,FGF10,SHH,ZEB2*

Supplementary Table 4, FUMA functional enrichment of genome-wide loci for phenotypes in GWAS Catalog.
Enrichment was performed with default settings on the set of genes annotated with GREAT for consistency with the other gene set enrichment analyses.
P-values were obtained from a hypergeometric test (one-tailed). Only significant (FDR < 0.05) terms are shown.

Description	HyperP	HyperFdrQ	ObsGenes	TotalGenes	Genes
Monobrow	3.51E-13	6.36E-10	8	77	WARS2,INSIG2,EN1,HOXD1,MTX2,SHOX2,CDC5L,RSPO3
Ossification of the posterior longitudinal ligament of the spine	3.25E-12	2.95E-09	5	11	PTHLH,CCDC91,HAO1,CDC5L,EMC2
Lung function (FVC)	1.05E-11	6.38E-09	9	184	PTHLH,CCDC91,ALX1,EN1,BMP2,FGF10,CDC5L,RUNX2,RSPO3
Heel bone mineral density	2.18E-11	8.15E-09	14	834	CYP26A1,CCDC91,FAR2,HMGA2,MEIS1,INSIG2,EN1,BMP2,HAO1,RSPO3,EYA4,CPED1,WNT16,ACTL7B
Chin dimples	2.25E-11	8.15E-09	7	74	HMGA2,LLPH,ZEB2,MTX2,TBL1XR1,KCNMB2,CDC5L
Male-pattern baldness	1.87E-10	5.66E-08	9	254	WARS2,PTHLH,FAR2,HOXD1,MTX2,CDC5L,RSPO3,EMC2,TMEM74
Waist circumference adjusted for BMI in non-smokers	1.20E-09	3.12E-07	6	70	WARS2,CCDC91,HMGA2,ADAMTSL3,BMP2,RSPO3
Waist circumference adjusted for BMI (joint analysis main effects and smoking interaction)	2.72E-09	6.18E-07	6	80	WARS2,CCDC91,HMGA2,ADAMTSL3,BMP2,RSPO3
Waist circumference adjusted for BMI (adjusted for smoking behaviour)	3.16E-09	6.38E-07	6	82	WARS2,CCDC91,HMGA2,ADAMTSL3,BMP2,RSPO3
Bone ultrasound measurement (velocity of sound)	3.52E-09	6.39E-07	4	13	CCDC91,EN1,RSPO3,WNT16
Bone mineral density (paediatric, total body less head)	6.71E-09	1.11E-06	4	15	PTHLH,RSPO3,CPED1,WNT16
Bone mineral density (paediatric, skull)	1.50E-08	2.27E-06	4	18	RSPO3,EYA4,CPED1,WNT16
Waist-to-hip ratio adjusted for BMI (adjusted for smoking behaviour)	7.95E-08	1.11E-05	5	70	WARS2,MEIS1,BMP2,RSPO3,EYA4
Bone mineral density (paediatric, upper limb)	1.91E-07	2.48E-05	3	8	RSPO3,CPED1,WNT16
Bone mineral density (paediatric, lower limb)	2.86E-07	3.46E-05	3	9	PTHLH,CPED1,WNT16
Height	9.77E-07	0.000110805	10	898	CCDC91,HMGA2,ADAMTSL3,MEIS1,BMP2,SHOX2,FGF18,CDC5L,RUNX2,SLC25A13
Hip circumference adjusted for BMI	1.07E-06	0.000114428	6	218	CCDC91,HMGA2,ADAMTSL3,BMP2,RSPO3,EYA4
Waist circumference adjusted for body mass index	1.46E-06	0.000142068	6	230	WARS2,CCDC91,HMGA2,ADAMTSL3,BMP2,RSPO3
Lumbar spine bone mineral density	1.49E-06	0.000142068	4	54	INSIG2,EN1,CPED1,WNT16
Waist-to-hip ratio adjusted for BMI (joint analysis for main effect and physical activity interaction)	1.72E-06	0.00015639	4	56	WARS2,MEIS1,BMP2,RSPO3
Femoral neck bone mineral density	4.23E-06	0.000365944	4	70	EN1,RSPO3,SLC25A13,WNT16
FEV1	7.51E-06	0.000592391	5	175	CCDC91,BMP2,FGF10,FGF18,CDC5L
Spherical equivalent or myopia (age of diagnosis)	7.51E-06	0.000592391	5	175	CYP26A1,MYOF,PCCA,ZEB2,BMP2
Waist circumference adjusted for BMI in active individuals	8.36E-06	0.000632323	4	83	CCDC91,ADAMTSL3,BMP2,RSPO3
Waist circumference adjusted for BMI (joint analysis main effects and physical activity interaction)	9.19E-06	0.000667338	4	85	CCDC91,ADAMTSL3,BMP2,RSPO3
Pulse pressure	9.96E-06	0.00069495	8	690	HMGA2,LLPH,ADAMTSL3,BMP2,SHOX2,TBL1XR1,RSPO3,TCF21
Fractures	1.09E-05	0.000735032	3	28	RSPO3,CPED1,WNT16
Refractive error	1.22E-05	0.000789704	3	29	CYP26A1,PCCA,BMP2
Waist-to-hip ratio adjusted for BMI in non-smokers	1.81E-05	0.00113355	3	33	WARS2,RSPO3,EYA4
Smooth-surface caries	1.98E-05	0.0012005	3	34	FERMT1,KIF6,BBS9
Pediatric bone mineral content (radius)	2.31E-05	0.001339972	2	5	CPED1,WNT16
Bone mineral density	2.36E-05	0.001339972	3	36	RSPO3,CPED1,WNT16
Waist-to-hip ratio adjusted for body mass index	2.93E-05	0.001612107	4	114	WARS2,MEIS1,BMP2,RSPO3
Waist-hip ratio	3.03E-05	0.001619237	4	115	WARS2,TSPAN19,MAP2K6,RSPO3
Intraocular pressure	3.36E-05	0.001744461	6	399	CYP26A1,MYOF,SPRED2,MEIS1,RUNX2,BBS9
TB-LM or TBLH-BMD (pleiotropy)	3.46E-05	0.001745841	2	6	CPED1,WNT16
Intracranial volume	4.84E-05	0.002375774	2	7	RSPO3,RNF146
Waist-to-hip ratio adjusted for BMI in active individuals	5.30E-05	0.002532139	3	47	WARS2,MEIS1,RSPO3
Blood urea nitrogen levels	7.69E-05	0.003580615	4	146	CYP26A1,MTX2,RSPO3,TCF21
Hip minimal joint space width	0.000103476	0.004695207	2	10	HAO1,RUNX2
Corneal astigmatism	0.000110312	0.004883336	3	60	ZEB2,BMPER,SHH
Bone properties (heel)	0.000126346	0.005459933	2	11	RSPO3,WNT16
Eye morphology	0.000151465	0.006393242	2	12	HOXD1,MTX2
Facial morphology traits (63 three-dimensional facial segments)	0.000178828	0.007055929	2	13	EYA4,DLX6
Upper eyelid morphology	0.000178828	0.007055929	2	13	HOXD1,MTX2
Food addiction	0.000178828	0.007055929	2	13	RSPO3,RNF146
LDL cholesterol levels in current drinkers	0.000197686	0.007634059	3	73	CYP26A1,MYOF,INSIG2
Subcortical brain region volumes	0.000240256	0.008908121	2	15	RSPO3,RNF146
Lung function (FEV1)	0.000240495	0.008908121	3	78	PTHLH,CCDC91,ALX1
Glaucoma (primary open-angle)	0.000249717	0.009064721	3	79	CYP26A1,MYOF,MEIS1
Glomerular filtration rate (creatinine)	0.000288887	0.010280973	3	83	CYP26A1,EYA4,RNF32
Triglyceride levels in current drinkers	0.000299262	0.01044538	3	84	CYP26A1,MYOF,RSPO3
Lumbar spine bone mineral density (trabecular)	0.000310576	0.010635747	2	17	PTHLH,CCDC91
Breast cancer	0.000333157	0.011197767	4	214	PTHLH,FGF10,MRPS30,RNF146
Triglyceride levels x alcohol consumption (drinkers vs non-drinkers) interaction (2df)	0.000378666	0.01202841	3	91	CYP26A1,MYOF,RSPO3
Waist-to-hip ratio adjusted for BMI	0.000385666	0.01202841	5	403	WARS2,KCNJ16,MEIS1,RSPO3,RNF146
Primary tooth development (time to first tooth eruption)	0.000389734	0.01202841	2	19	HMGA2,KCNJ16
Primary tooth development (number of teeth)	0.000389734	0.01202841	2	19	HMGA2,KCNJ16
Triglyceride levels x alcohol consumption (regular vs non-regular drinkers) interaction (2df)	0.000391006	0.01202841	3	92	CYP26A1,MYOF,RSPO3
LDL cholesterol levels x alcohol consumption (regular vs non-regular drinkers) interaction (2df)	0.00042957	0.012871965	3	95	CYP26A1,MYOF,INSIG2
Bone ultrasound measurement (broadband ultrasound attenuation)	0.000432611	0.012871965	2	20	RSPO3,WNT16
LDL cholesterol levels x alcohol consumption (drinkers vs non-drinkers) interaction (2df)	0.000484674	0.014188428	3	99	CYP26A1,MYOF,INSIG2
Mosquito bite size	0.000513844	0.014803609	3	101	INSIG2,EN1,RUNX2
Nose morphology	0.000524929	0.014886663	2	22	HAO1,CDC5L
Alzheimer disease and age of onset	0.000625954	0.017478556	2	24	FGF10,CDC5L
QT interval	0.000731071	0.019927968	3	114	FAR2,HAO1,RSPO3
Osteoarthritis (hip)	0.000735633	0.019927968	2	26	PTHLH,RUNX2
Peak expiratory flow	0.000749841	0.020014145	3	115	CCDC91,BMP2,FGF18
HDL cholesterol levels in current drinkers	0.000788296	0.020735612	3	117	CYP26A1,MYOF,RSPO3
HDL cholesterol levels x alcohol consumption (regular vs non-regular drinkers) interaction (2df)	0.001045465	0.027107427	3	129	CYP26A1,MYOF,RSPO3
Waist-to-hip ratio adjusted for BMI (joint analysis main effects and smoking interaction)	0.001116078	0.028530723	2	32	WARS2,RSPO3
Bone mineral density (spine)	0.001186914	0.029510269	2	33	EN1,RSPO3
Cerebral amyloid deposition in APOEe4 non-carriers (PET imaging)	0.001186914	0.029510269	2	33	MAP2K6,KCNJ16
HDL cholesterol levels x alcohol consumption (drinkers vs non-drinkers) interaction (2df)	0.001296052	0.031788302	3	139	CYP26A1,MYOF,RSPO3
Estimated glomerular filtration rate	0.001362229	0.032965936	5	534	CYP26A1,FGF10,MRPS30,EYA4,SHH
Chronic obstructive pulmonary disease	0.001434322	0.03425387	3	144	CCDC91,ADAMTSL3,FGF18
Triglyceride levels	0.001463042	0.034485982	3	145	CYP26A1,MYOF,RSPO3
Hip circumference	0.00158153	0.036800989	3	149	HMGA2,ADAMTSL3,EYA4
Chronotype	0.001626308	0.037363903	5	556	MEIS1,SHOX2,TBL1XR1,BBS9,DLX6
HDL cholesterol levels	0.001737885	0.039019481	3	154	CYP26A1,MYOF,RSPO3
Corneal structure	0.001741365	0.039019481	2	40	TBL1XR1,KCNMB2
Polycystic ovary syndrome	0.001828867	0.040480411	2	41	FAR2,HMGA2
Dentures	0.002199358	0.047521845	2	45	HAO1,FGF10
Number of decayed, missing and filled tooth surfaces or use of dentures	0.002199358	0.047521845	2	45	HAO1,FGF10

Supplementary Table 5: Cell type and tissue metadata

For each cell type and tissue used in the H3K27ac-based analyses, we have listed the sample class, type, ID, and URL from either the GEO accession browser or the Roadmap Epigenomics Project.

Sample Name	Sample Class	Sample type	ID	URL
CS17-12341	PrimaryTissue	CRANIOFACIAL	GSM2576968	https://www.ncbi.nlm.nih.gov/geo/query/acc.cgi?acc=GSM2576968
CNCC	hESC/iPSC derived	Cranial neural crest cells	GSM1817151	https://www.ncbi.nlm.nih.gov/geo/query/acc.cgi?acc=GSM1817151
CS17-12331	PrimaryTissue	CRANIOFACIAL	GSM2576967	https://www.ncbi.nlm.nih.gov/geo/query/acc.cgi?acc=GSM2576967
CS15-13019	PrimaryTissue	CRANIOFACIAL	GSM2576965	https://www.ncbi.nlm.nih.gov/geo/query/acc.cgi?acc=GSM2576965
CS17-12611	PrimaryTissue	CRANIOFACIAL	GSM2576969	https://www.ncbi.nlm.nih.gov/geo/query/acc.cgi?acc=GSM2576969
CNCC	hESC/iPSC derived	Cranial neural crest cells	GSM1817152	https://www.ncbi.nlm.nih.gov/geo/query/acc.cgi?acc=GSM1817152
CS20-12104	PrimaryTissue	CRANIOFACIAL	GSM2576954	https://www.ncbi.nlm.nih.gov/geo/query/acc.cgi?acc=GSM2576954
CNCC	hESC/iPSC derived	Cranial neural crest cells	GSM1817153	https://www.ncbi.nlm.nih.gov/geo/query/acc.cgi?acc=GSM1817153
CNCC	hESC/iPSC derived	Cranial neural crest cells	GSM1817154	https://www.ncbi.nlm.nih.gov/geo/query/acc.cgi?acc=GSM1817154
CS17-12191	PrimaryTissue	CRANIOFACIAL	GSM2576950	https://www.ncbi.nlm.nih.gov/geo/query/acc.cgi?acc=GSM2576950
CNCC	hESC/iPSC derived	Cranial neural crest cells	GSM1817155	https://www.ncbi.nlm.nih.gov/geo/query/acc.cgi?acc=GSM1817155
CNCC	hESC/iPSC derived	Cranial neural crest cells	GSM1817156	https://www.ncbi.nlm.nih.gov/geo/query/acc.cgi?acc=GSM1817156
CNCC	hESC/iPSC derived	Cranial neural crest cells	GSM1817157	https://www.ncbi.nlm.nih.gov/geo/query/acc.cgi?acc=GSM1817157
CS14-12913	PrimaryTissue	CRANIOFACIAL	GSM2576963	https://www.ncbi.nlm.nih.gov/geo/query/acc.cgi?acc=GSM2576963
CS15-13128	PrimaryTissue	CRANIOFACIAL	GSM2576966	https://www.ncbi.nlm.nih.gov/geo/query/acc.cgi?acc=GSM2576966
CNCC	hESC/iPSC derived	Cranial neural crest cells	GSM1817158	https://www.ncbi.nlm.nih.gov/geo/query/acc.cgi?acc=GSM1817158
CNCC	hESC/iPSC derived	Cranial neural crest cells	GSM1817159	https://www.ncbi.nlm.nih.gov/geo/query/acc.cgi?acc=GSM1817159
CS13-12690	PrimaryTissue	CRANIOFACIAL	GSM2576957	https://www.ncbi.nlm.nih.gov/geo/query/acc.cgi?acc=GSM2576957
CS13-12877	PrimaryTissue	CRANIOFACIAL	GSM2576960	https://www.ncbi.nlm.nih.gov/geo/query/acc.cgi?acc=GSM2576960
CS13-12830	PrimaryTissue	CRANIOFACIAL	GSM2576959	https://www.ncbi.nlm.nih.gov/geo/query/acc.cgi?acc=GSM2576959
CNCC	hESC/iPSC derived	Cranial neural crest cells	GSM1817160	https://www.ncbi.nlm.nih.gov/geo/query/acc.cgi?acc=GSM1817160
Duodenum_Smooth_Muscle	PrimaryTissue	GI_DUODENUM	E078	https://egg2.wustl.edu/roadmap/data/byFileType/alignments/consolidated/E078-H3K27ac.tagAlign.gz
CS13-12829	PrimaryTissue	CRANIOFACIAL	GSM2576958	https://www.ncbi.nlm.nih.gov/geo/query/acc.cgi?acc=GSM2576958
CNCC	hESC/iPSC derived	Cranial neural crest cells	GSM1817161	https://www.ncbi.nlm.nih.gov/geo/query/acc.cgi?acc=GSM1817161
NHDF-Ad_Adult_Dermal_Fibroblasts	PrimaryCulture	SKIN	E126	https://egg2.wustl.edu/roadmap/data/byFileType/alignments/consolidated/E126-H3K27ac.tagAlign.gz
CS14-12408	PrimaryTissue	CRANIOFACIAL	GSM2576961	https://www.ncbi.nlm.nih.gov/geo/query/acc.cgi?acc=GSM2576961
Colon_Smooth_Muscle	PrimaryTissue	GI_COLON	E076	https://egg2.wustl.edu/roadmap/data/byFileType/alignments/consolidated/E076-H3K27ac.tagAlign.gz
Thymus	PrimaryTissue	THYMUS	E112	https://egg2.wustl.edu/roadmap/data/byFileType/alignments/consolidated/E112-H3K27ac.tagAlign.gz
Skeletal_Muscle_Female	PrimaryTissue	MUSCLE	E108	https://egg2.wustl.edu/roadmap/data/byFileType/alignments/consolidated/E108-H3K27ac.tagAlign.gz
CS15-13000	PrimaryTissue	CRANIOFACIAL	GSM2576964	https://www.ncbi.nlm.nih.gov/geo/query/acc.cgi?acc=GSM2576964
Stomach_Smooth_Muscle	PrimaryTissue	GI_STOMACH	E111	https://egg2.wustl.edu/roadmap/data/byFileType/alignments/consolidated/E111-H3K27ac.tagAlign.gz
Fetal_Placenta	PrimaryTissue	PLACENTA	E091	https://egg2.wustl.edu/roadmap/data/byFileType/alignments/consolidated/E091-H3K27ac.tagAlign.gz
CS14-12709	PrimaryTissue	CRANIOFACIAL	GSM2576962	https://www.ncbi.nlm.nih.gov/geo/query/acc.cgi?acc=GSM2576962
CD56	PrimaryCell	BLOOD	E046	https://egg2.wustl.edu/roadmap/data/byFileType/alignments/consolidated/E046-H3K27ac.tagAlign.gz
Gastric	PrimaryTissue	GI_STOMACH	E094	https://egg2.wustl.edu/roadmap/data/byFileType/alignments/consolidated/E094-H3K27ac.tagAlign.gz
Pancreas	PrimaryTissue	PANCREAS	E098	https://egg2.wustl.edu/roadmap/data/byFileType/alignments/consolidated/E098-H3K27ac.tagAlign.gz
Placenta_Amnion	PrimaryTissue	PLACENTA	E099	https://egg2.wustl.edu/roadmap/data/byFileType/alignments/consolidated/E099-H3K27ac.tagAlign.gz
Fetal_Muscle_Trunk	PrimaryTissue	MUSCLE	E089	https://egg2.wustl.edu/roadmap/data/byFileType/alignments/consolidated/E089-H3K27ac.tagAlign.gz
Fibroblast_1	PrimaryCulture	SKIN	E055	https://egg2.wustl.edu/roadmap/data/byFileType/alignments/consolidated/E055-H3K27ac.tagAlign.gz
Fetal_osteoplast_diff_1		BONE	GSM2188465	https://www.ncbi.nlm.nih.gov/geo/query/acc.cgi?acc=GSM2188465
Osteoblasts	PrimaryCulture	BONE	E129	https://egg2.wustl.edu/roadmap/data/byFileType/alignments/consolidated/E129-H3K27ac.tagAlign.gz
CS13-12383	PrimaryTissue	CRANIOFACIAL	GSM2576956	https://www.ncbi.nlm.nih.gov/geo/query/acc.cgi?acc=GSM2576956
hESC_Derived_CD56+_Ectoderm	ESCDerived	ESC_DERIVED	E012	https://egg2.wustl.edu/roadmap/data/byFileType/alignments/consolidated/E012-H3K27ac.tagAlign.gz
MSC-derived_chondrocytes	PrimaryCulture	STROMAL_CONNECTIVE	E049	https://egg2.wustl.edu/roadmap/data/byFileType/alignments/consolidated/E049-H3K27ac.tagAlign.gz
Esophagus	PrimaryTissue	GI_ESOPHAGUS	E079	https://egg2.wustl.edu/roadmap/data/byFileType/alignments/consolidated/E079-H3K27ac.tagAlign.gz
F2-N2108	PrimaryTissue	CRANIOFACIAL	GSM2576955	https://www.ncbi.nlm.nih.gov/geo/query/acc.cgi?acc=GSM2576955
Skeletal_Muscle_Myoblasts	PrimaryCulture	MUSCLE	E120	https://egg2.wustl.edu/roadmap/data/byFileType/alignments/consolidated/E120-H3K27ac.tagAlign.gz
NH-A_Astrocytes	PrimaryCulture	BRAIN	E125	https://egg2.wustl.edu/roadmap/data/byFileType/alignments/consolidated/E125-H3K27ac.tagAlign.gz
Psoas_Muscle	PrimaryTissue	MUSCLE	E100	https://egg2.wustl.edu/roadmap/data/byFileType/alignments/consolidated/E100-H3K27ac.tagAlign.gz
NHLF_Lung_Fibroblasts	PrimaryCulture	LUNG	E128	https://egg2.wustl.edu/roadmap/data/byFileType/alignments/consolidated/E128-H3K27ac.tagAlign.gz
Fetal_Stomach	PrimaryTissue	GI_STOMACH	E092	https://egg2.wustl.edu/roadmap/data/byFileType/alignments/consolidated/E092-H3K27ac.tagAlign.gz
Fetal_osteoblast_undiff_2		BONE	GSM2188471	https://www.ncbi.nlm.nih.gov/geo/query/acc.cgi?acc=GSM2188471
iPS-18	PrimaryCulture	IPSC	E019	https://egg2.wustl.edu/roadmap/data/byFileType/alignments/consolidated/E019-H3K27ac.tagAlign.gz
Peripheral_Blood_Mononuclear	PrimaryCell	BLOOD	E062	https://egg2.wustl.edu/roadmap/data/byFileType/alignments/consolidated/E062-H3K27ac.tagAlign.gz
Fetal_osteoblast_undiff_1		BONE	GSM2188470	https://www.ncbi.nlm.nih.gov/geo/query/acc.cgi?acc=GSM2188470
Skeletal_Muscle_Myotubes	PrimaryCulture	MUSCLE	E121	https://egg2.wustl.edu/roadmap/data/byFileType/alignments/consolidated/E121-H3K27ac.tagAlign.gz
Fetal_Muscle_Leg	PrimaryTissue	MUSCLE_LEG	E090	https://egg2.wustl.edu/roadmap/data/byFileType/alignments/consolidated/E090-H3K27ac.tagAlign.gz
Adult_Liver	PrimaryTissue	LIVER	E066	https://egg2.wustl.edu/roadmap/data/byFileType/alignments/consolidated/E066-H3K27ac.tagAlign.gz
Fetal_osteoblast_diff_2		BONE	GSM2188466	https://www.ncbi.nlm.nih.gov/geo/query/acc.cgi?acc=GSM2188466
Surface ectoderm	ESCDerived	ESC_DERIVED	GSM3455884	https://www.ncbi.nlm.nih.gov/geo/query/acc.cgi?acc=GSE119997
Ovary	PrimaryTissue	OVARY	E097	https://egg2.wustl.edu/roadmap/data/byFileType/alignments/consolidated/E097-H3K27ac.tagAlign.gz
HUES64	PrimaryCulture	ESC	E016	https://egg2.wustl.edu/roadmap/data/byFileType/alignments/consolidated/E016-H3K27ac.tagAlign.gz
MSC-derived-osteoblast		BONE	GSM2360000	https://www.ncbi.nlm.nih.gov/geo/query/acc.cgi?acc=GSM2360000
iPS_DF_6.9	PrimaryCulture	IPSC	E021	https://egg2.wustl.edu/roadmap/data/byFileType/alignments/consolidated/E021-H3K27ac.tagAlign.gz
Fibroblast_2	PrimaryCulture	SKIN	E056	https://egg2.wustl.edu/roadmap/data/byFileType/alignments/consolidated/E056-H3K27ac.tagAlign.gz
iPS-20b	PrimaryCulture	IPSC	E020	https://egg2.wustl.edu/roadmap/data/byFileType/alignments/consolidated/E020-H3K27ac.tagAlign.gz
H1_Derived_MSCs	ESCDerived	ESC_DERIVED	E006	https://egg2.wustl.edu/roadmap/data/byFileType/alignments/consolidated/E006-H3K27ac.tagAlign.gz
Surface ectoderm	ESCDerived	ESC_DERIVED	GSM3455885	https://www.ncbi.nlm.nih.gov/geo/query/acc.cgi?acc=GSE119997
NHEK-Epidermal_Keratinocytes	PrimaryCulture	SKIN	E127	https://egg2.wustl.edu/roadmap/data/byFileType/alignments/consolidated/E127-H3K27ac.tagAlign.gz
HUES48	PrimaryCulture	ESC	E014	https://egg2.wustl.edu/roadmap/data/byFileType/alignments/consolidated/E014-H3K27ac.tagAlign.gz
Spleen	PrimaryTissue	SPLEEN	E113	https://egg2.wustl.edu/roadmap/data/byFileType/alignments/consolidated/E113-H3K27ac.tagAlign.gz
Fetal_Thymus	PrimaryTissue	THYMUS	E093	https://egg2.wustl.edu/roadmap/data/byFileType/alignments/consolidated/E093-H3K27ac.tagAlign.gz
HMEC_Mammary_Epithelial	PrimaryCulture	BREAST	E119	https://egg2.wustl.edu/roadmap/data/byFileType/alignments/consolidated/E119-H3K27ac.tagAlign.gz
HUES6	PrimaryCulture	ESC	E015	https://egg2.wustl.edu/roadmap/data/byFileType/alignments/consolidated/E015-H3K27ac.tagAlign.gz
H1	PrimaryCulture	ESC	E003	https://egg2.wustl.edu/roadmap/data/byFileType/alignments/consolidated/E003-H3K27ac.tagAlign.gz
HUVEC	PrimaryCulture	VASCULAR	E122	https://egg2.wustl.edu/roadmap/data/byFileType/alignments/consolidated/E122-H3K27ac.tagAlign.gz
A549_Lung_Carcinoma	CellLine	LUNG	E114	https://egg2.wustl.edu/roadmap/data/byFileType/alignments/consolidated/E114-H3K27ac.tagAlign.gz
Rectal_Smooth_Muscle	PrimaryTissue	GI_RECTUM	E103	https://egg2.wustl.edu/roadmap/data/byFileType/alignments/consolidated/E103-H3K27ac.tagAlign.gz
HeLa-S3_Cervical_Carcinoma	CellLine	CERVIX	E117	https://egg2.wustl.edu/roadmap/data/byFileType/alignments/consolidated/E117-H3K27ac.tagAlign.gz
Fetal_Intestine_Large	PrimaryTissue	GI_INTESTINE	E084	https://egg2.wustl.edu/roadmap/data/byFileType/alignments/consolidated/E084-H3K27ac.tagAlign.gz
H1_BMP4_Derived_Mesendoderm	ESCDerived	ESC_DERIVED	E004	https://egg2.wustl.edu/roadmap/data/byFileType/alignments/consolidated/E004-H3K27ac.tagAlign.gz
hESC_Derived_CD184+_Endoderm	ESCDerived	ESC_DERIVED	E011	https://egg2.wustl.edu/roadmap/data/byFileType/alignments/consolidated/E011-H3K27ac.tagAlign.gz
Fetal_Intestine_Small	PrimaryTissue	GI_INTESTINE	E085	https://egg2.wustl.edu/roadmap/data/byFileType/alignments/consolidated/E085-H3K27ac.tagAlign.gz
Fetal_Adrenal_Gland	PrimaryTissue	ADRENAL	E080	https://egg2.wustl.edu/roadmap/data/byFileType/alignments/consolidated/E080-H3K27ac.tagAlign.gz
Right_Ventricle	PrimaryTissue	HEART	E105	https://egg2.wustl.edu/roadmap/data/byFileType/alignments/consolidated/E105-H3K27ac.tagAlign.gz
Sigmoid_Colon	PrimaryTissue	GI_COLON	E106	https://egg2.wustl.edu/roadmap/data/byFileType/alignments/consolidated/E106-H3K27ac.tagAlign.gz
Left_Ventricle	PrimaryTissue	HEART	E095	https://egg2.wustl.edu/roadmap/data/byFileType/alignments/consolidated/E095-H3K27ac.tagAlign.gz
LHX2+_astrocytes		BRAIN		https://genome.ucsc.edu/s/nottalexi/glassLab_BrainCellTypes_hg19
Rectal_Mucosa.Donor_29	PrimaryTissue	GI_RECTUM	E101	https://egg2.wustl.edu/roadmap/data/byFileType/alignments/consolidated/E101-H3K27ac.tagAlign.gz
Colonic_Mucosa	PrimaryTissue	GI_COLON	E075	https://egg2.wustl.edu/roadmap/data/byFileType/alignments/consolidated/E075-H3K27ac.tagAlign.gz
Lung	PrimaryTissue	LUNG	E096	https://egg2.wustl.edu/roadmap/data/byFileType/alignments/consolidated/E096-H3K27ac.tagAlign.gz
H1_BMP4_Derived_Trophoblast	ESCDerived	ESC_DERIVED	E005	https://egg2.wustl.edu/roadmap/data/byFileType/alignments/consolidated/E005-H3K27ac.tagAlign.gz
CD8_Memory	PrimaryCell	BLOOD	E048	https://egg2.wustl.edu/roadmap/data/byFileType/alignments/consolidated/E048-H3K27ac.tagAlign.gz
HepG2_Hepatocellular_Carcinoma	CellLine	LIVER	E118	https://egg2.wustl.edu/roadmap/data/byFileType/alignments/consolidated/E118-H3K27ac.tagAlign.gz
hESC_Derived_CD56+_Mesoderm	ESCDerived	ESC_DERIVED	E013	https://egg2.wustl.edu/roadmap/data/byFileType/alignments/consolidated/E013-H3K27ac.tagAlign.gz
Rectal_Mucosa.Donor_31	PrimaryTissue	GI_RECTUM	E102	https://egg2.wustl.edu/roadmap/data/byFileType/alignments/consolidated/E102-H3K27ac.tagAlign.gz
IMR90	CellLine	LUNG	E017	https://egg2.wustl.edu/roadmap/data/byFileType/alignments/consolidated/E017-H3K27ac.tagAlign.gz
MSC-derived-osteoblast		BONE	GSM2360000	https://www.ncbi.nlm.nih.gov/geo/query/acc.cgi?acc=GSM2360000
Pancreatic_Islets	PrimaryTissue	PANCREAS	E087	https://egg2.wustl.edu/roadmap/data/byFileType/alignments/consolidated/E087-H3K27ac.tagAlign.gz
K562_Leukemia	PrimaryCulture	BLOOD	E123	https://egg2.wustl.edu/roadmap/data/byFileType/alignments/consolidated/E123-H3K27ac.tagAlign.gz
H1_Derived_Neuronal_Progenitor	ESCDerived	ESC_DERIVED	E007	https://egg2.wustl.edu/roadmap/data/byFileType/alignments/consolidated/E007-H3K27ac.tagAlign.gz
Monocytes-CD14+_RO01746	PrimaryCell	BLOOD	E124	https://egg2.wustl.edu/roadmap/data/byFileType/alignments/consolidated/E124-H3K27ac.tagAlign.gz
Small_Intestine	PrimaryTissue	GI_INTESTINE	E109	https://egg2.wustl.edu/roadmap/data/byFileType/alignments/consolidated/E109-H3K27ac.tagAlign.gz
Right_Atrium	PrimaryTissue	HEART	E104	https://egg2.wustl.edu/roadmap/data/byFileType/alignments/consolidated/E104-H3K27ac.tagAlign.gz
Keratinocyte_3	PrimaryCulture	SKIN	E058	https://egg2.wustl.edu/roadmap/data/byFileType/alignments/consolidated/E058-H3K27ac.tagAlign.gz
Brain_Hippocampus_Middle	PrimaryTissue	BRAIN	E071	https://egg2.wustl.edu/roadmap/data/byFileType/alignments/consolidated/E071-H3K27ac.tagAlign.gz
CD4+_CD25-_Th	PrimaryCell	BLOOD	E043	https://egg2.wustl.edu/roadmap/data/byFileType/alignments/consolidated/E043-H3K27ac.tagAlign.gz
Melanocyte_3	PrimaryCulture	SKIN	E061	https://egg2.wustl.edu/roadmap/data/byFileType/alignments/consolidated/E061-H3K27ac.tagAlign.gz
CD4+_CD25-_IL17-_PMA-stimulated__Th17	PrimaryCell	BLOOD	E041	https://egg2.wustl.edu/roadmap/data/byFileType/alignments/consolidated/E041-H3K27ac.tagAlign.gz
Melanocyte_1	PrimaryCulture	SKIN	E059	https://egg2.wustl.edu/roadmap/data/byFileType/alignments/consolidated/E059-H3K27ac.tagAlign.gz
iPS_DF_19.11	PrimaryCulture	IPSC	E022	https://egg2.wustl.edu/roadmap/data/byFileType/alignments/consolidated/E022-H3K27ac.tagAlign.gz
Adipose_Nuclei	PrimaryTissue	FAT	E063	https://egg2.wustl.edu/roadmap/data/byFileType/alignments/consolidated/E063-H3K27ac.tagAlign.gz
CD4_Naive	PrimaryCell	BLOOD	E038	https://egg2.wustl.edu/roadmap/data/byFileType/alignments/consolidated/E038-H3K27ac.tagAlign.gz
Ex-vivo_microglia		BRAIN		https://genome.ucsc.edu/s/nottalexi/glassLab_BrainCellTypes_hg19
CD4_Memory	PrimaryCell	BLOOD	E037	https://egg2.wustl.edu/roadmap/data/byFileType/alignments/consolidated/E037-H3K27ac.tagAlign.gz
Brain_Inferior_Temporal_Lobe	PrimaryTissue	BRAIN	E072	https://egg2.wustl.edu/roadmap/data/byFileType/alignments/consolidated/E072-H3K27ac.tagAlign.gz
Aorta	PrimaryTissue	VASCULAR	E065	https://egg2.wustl.edu/roadmap/data/byFileType/alignments/consolidated/E065-H3K27ac.tagAlign.gz
CD4+_CD25-_CD45RO+_Memory	PrimaryCell	BLOOD	E040	https://egg2.wustl.edu/roadmap/data/byFileType/alignments/consolidated/E040-H3K27ac.tagAlign.gz
CD4+_CD25int_CD127+_Tmem	PrimaryCell	BLOOD	E045	https://egg2.wustl.edu/roadmap/data/byFileType/alignments/consolidated/E045-H3K27ac.tagAlign.gz
CD19_Peripheral_UW	PrimaryCell	BLOOD	E032	https://egg2.wustl.edu/roadmap/data/byFileType/alignments/consolidated/E032-H3K27ac.tagAlign.gz
PU1+_microglia		BRAIN		https://genome.ucsc.edu/s/nottalexi/glassLab_BrainCellTypes_hg19
CD14	PrimaryCell	BLOOD	E029	https://egg2.wustl.edu/roadmap/data/byFileType/alignments/consolidated/E029-H3K27ac.tagAlign.gz
CD3_Peripheral_UW	PrimaryCell	BLOOD	E034	https://egg2.wustl.edu/roadmap/data/byFileType/alignments/consolidated/E034-H3K27ac.tagAlign.gz
GM12878_Lymphoblastoid	PrimaryCulture	BLOOD	E116	https://egg2.wustl.edu/roadmap/data/byFileType/alignments/consolidated/E116-H3K27ac.tagAlign.gz
H9	PrimaryCulture	ESC	E008	https://egg2.wustl.edu/roadmap/data/byFileType/alignments/consolidated/E008-H3K27ac.tagAlign.gz
OLIG2+_oligodendrocytes		BRAIN		https://genome.ucsc.edu/s/nottalexi/glassLab_BrainCellTypes_hg19
Dnd41_TCell_Leukemia	CellLine	BLOOD	E115	https://egg2.wustl.edu/roadmap/data/byFileType/alignments/consolidated/E115-H3K27ac.tagAlign.gz
Bone_Marrow_Derived_MSC	PrimaryCulture	STROMAL_CONNECTIVE	E026	https://egg2.wustl.edu/roadmap/data/byFileType/alignments/consolidated/E026-H3K27ac.tagAlign.gz
MSC-derived-osteoblast		BONE	GSM2360000	https://www.ncbi.nlm.nih.gov/geo/query/acc.cgi?acc=GSM2360000
Brain_Anterior_Caudate	PrimaryTissue	BRAIN	E068	https://egg2.wustl.edu/roadmap/data/byFileType/alignments/consolidated/E068-H3K27ac.tagAlign.gz
Brain_Mid_Frontal_Lobe	PrimaryTissue	BRAIN	E073	https://egg2.wustl.edu/roadmap/data/byFileType/alignments/consolidated/E073-H3K27ac.tagAlign.gz
Brain_Cingulate_Gyrus	PrimaryTissue	BRAIN	E069	https://egg2.wustl.edu/roadmap/data/byFileType/alignments/consolidated/E069-H3K27ac.tagAlign.gz
CD4+_CD25-_IL17+_PMA-_stimulated_Th17	PrimaryCell	BLOOD	E042	https://egg2.wustl.edu/roadmap/data/byFileType/alignments/consolidated/E042-H3K27ac.tagAlign.gz
CD8_Naive	PrimaryCell	BLOOD	E047	https://egg2.wustl.edu/roadmap/data/byFileType/alignments/consolidated/E047-H3K27ac.tagAlign.gz
peripheral_PU1+_microglia		BRAIN		https://genome.ucsc.edu/s/nottalexi/glassLab_BrainCellTypes_hg19
Mobilized_CD34_Female	PrimaryCell	BLOOD	E050	https://egg2.wustl.edu/roadmap/data/byFileType/alignments/consolidated/E050-H3K27ac.tagAlign.gz
Brain_Substantia_Nigra	PrimaryTissue	BRAIN	E074	https://egg2.wustl.edu/roadmap/data/byFileType/alignments/consolidated/E074-H3K27ac.tagAlign.gz
CD4+_CD25+_CD127-_Treg	PrimaryCell	BLOOD	E044	https://egg2.wustl.edu/roadmap/data/byFileType/alignments/consolidated/E044-H3K27ac.tagAlign.gz
Brain_Angular_Gyrus	PrimaryTissue	BRAIN	E067	https://egg2.wustl.edu/roadmap/data/byFileType/alignments/consolidated/E067-H3K27ac.tagAlign.gz
NEUN+_neurons		BRAIN		https://genome.ucsc.edu/s/nottalexi/glassLab_BrainCellTypes_hg19
CD4+_CD25-_CD45RA+_Naive	PrimaryCell	BLOOD	E039	https://egg2.wustl.edu/roadmap/data/byFileType/alignments/consolidated/E039-H3K27ac.tagAlign.gz

Supplementary Table 6, Differential Gene Expression Analysis and Annotation

Only results for candidate genes at GWAS loci are shown. P-values (pvalue) were obtained by the Wald test (one-tailed) and adjusted for 5% FDR (padj).

HGNC.symbol	MGI.symbol	ENSEMBL_ID	baseMean	log2FoldChange	lfcSE	pvalue	padj	Mouse.entrez	GWAS_Gene_Location	Significant (p<0.05)
SHOX2	*Shox2*	ENSMUSG00000027833	186.6281678	-1.961985802	0.217809	1.06E-20	2.25E-17	20429	3:157756785 (+349375)	Significant
EYA4	*Eya4*	ENSMUSG00000010461	521.5121264	-1.898093655	0.211458	1.35E-20	2.57E-17	14051	6:133334925 (+93339)	Significant
ALX1	*Alx1*	ENSMUSG00000036602	1003.908768	-1.369263892	0.178083	6.98E-16	9.52E-13	216285	12:85182714 (-97393)	Significant
ADAMTSL3	*Adamtsl3*	ENSMUSG00000070469	484.7326861	-1.009361225	0.134229	2.66E-15	3.18E-12	269959	15:83868247 (+214161)	Significant
EN1	*En1*	ENSMUSG00000058665	809.55995	-0.610419208	0.110052	1.44E-09	4.52E-07	13798	2:118432725 (+414953)	Significant
BMPER	*Bmper*	ENSMUSG00000031963	941.7123524	0.680440065	0.124813	2.36E-09	6.85E-07	73230	7:33219926 (-684985)	Significant
RSPO3	*Rspo3*	ENSMUSG00000019880	511.4837596	0.438604285	0.145589	0.000115	0.006857	72780	6:127134977 (+16373)	Significant
CPED1	*Cped1*	ENSMUSG00000062980	2679.052509	-0.338341744	0.114856	0.000166	0.009085	214642	7:121228465 (+239788)	Significant
FGF10	*Fgf10*	ENSMUSG00000021732	161.757254	-0.519263903	0.199506	0.000342	0.016076	14165	5:44441601 (-52804)	Significant
ZEB2	*Zeb2*	ENSMUSG00000026872	4512.375199	-0.343260927	0.14353	0.000736	0.028076	24136	2:144895720 (-375325)	Significant
HMGA2	*Hmga2*	ENSMUSG00000056758	497.1701489	-0.480088773	0.224964	0.001066	0.036253	15364	12:65980466 (+156006)	Significant
ZIC2	*Zic2*	ENSMUSG00000061524	1342.315039	0.492638855	0.261648	0.001734	0.050856	22772	NA	Not_Significant
INSIG2	*Insig2*	ENSMUSG00000003721	1749.842925	-0.229730521	0.139788	0.005115	0.110176	72999	2:118432725 (+344251)	Not_Significant
SLC25A13	*Slc25a13*	ENSMUSG00000015112	600.8825128	-0.198635436	0.148408	0.009434	0.166574	50799	7:96585031 (-262884)	Not_Significant
MYOF	*Myof*	ENSMUSG00000048612	2687.661632	0.158505903	0.106058	0.012323	0.201572	226101	10:93190051 (+292143)	Not_Significant
HOXD1	*Hoxd1*	ENSMUSG00000042448	20.30214666	0.023541293	0.078989	0.013348	0.212527	15429	2:176247091 (+58512)	Not_Significant
RSPO2	*Rspo2*	ENSMUSG00000051920	208.0692113	0.037560319	0.086684	0.024396	0.314185	239405	NA	Not_Significant
MAP2K6	*Map2k6*	ENSMUSG00000020623	427.5181705	-0.131148908	0.141051	0.026719	0.33084	26399	17:70079183 (+664485)	Not_Significant
BBS9	*Bbs9*	ENSMUSG00000035919	383.8409333	0.10393438	0.13797	0.03826	0.40231	319845	7:33219926 (+90394)	Not_Significant
WNT16	*Wnt16*	ENSMUSG00000029671	60.05923877	-0.024086451	0.078496	0.074695	0.575449	93735	7:121228465 (-100538)	Not_Significant
BMP2	*Bmp2*	ENSMUSG00000027358	1230.964433	-0.073054882	0.103488	0.07539	0.575934	12156	20:6608452 (-159212), 20:71	Not_Significant
RUNX2	*Runx2*	ENSMUSG00000039153	7460.67182	-0.081568483	0.096179	0.075789	0.577597	12393	6:44842621 (-485748)	Not_Significant
PTHLH	*Pthlh*	ENSMUSG00000048776	75.89475878	0.028168746	0.0791	0.139993	0.759646	19227	12:28085972 (-113267)	Not_Significant
FGF18	*Fgf18*	ENSMUSG00000057967	263.7036433	-0.043569852	0.084454	0.146319	0.771518	14172	5:171747250 (+327594)	Not_Significant
CCDC91	*Ccdc91*	ENSMUSG00000030301	588.4092486	0.055474583	0.080832	0.162017	0.797798	67015	12:28085972 (-104474), 12:2	Not_Significant
EMC2	*Emc2*	ENSMUSG00000022337	896.3081206	0.052547126	0.071888	0.20034	0.86085	66736	8:108534556 (+90932)	Not_Significant
ELP1	*Elp1*	ENSMUSG00000028431	2411.053868	-0.035629939	0.059826	0.359931	0.975024	230233	NA	Not_Significant
DAAM2	*Daam2*	ENSMUSG00000040260	4072.807593	0.036787016	0.065074	0.382336	0.983601	76441	NA	Not_Significant
CEP55	*Cep55*	ENSMUSG00000024989	524.3172432	0.027423907	0.069892	0.403912	0.988949	74107	NA	Not_Significant
CDC5L	*Gm9048*	ENSMUSG00000112495	49.83599739	-0.012904988	0.073397	0.446665	0.997988	668213	6:44842621 (+455096)	Not_Significant
CDC5L	*Gm9046*	ENSMUSG00000112781	17.90490257	-0.007819706	0.073734	0.450278	0.998131	668209	6:44842621 (+455096)	Not_Significant
BCL11B	*Bcl11b*	ENSMUSG00000048251	1209.984933	0.013545338	0.064972	0.682398	0.999804	58208	14:98408346 (+862882)	Not_Significant
CDC5L	*Cdc5l*	ENSMUSG00000023932	2237.356163	-0.021734001	0.058165	0.560948	0.999804	71702	6:44842621 (+455096)	Not_Significant
CDC5L	*Gm32717*	ENSMUSG00000112216	57.70725934	-0.006740901	0.072837	0.638351	0.999804	102635357	6:44842621 (+455096)	Not_Significant
CDC5L	*Gm32802*	ENSMUSG00000112252	57.86751898	-0.007015272	0.07288	0.624294	0.999804	102635473	6:44842621 (+455096)	Not_Significant
CDC5L	*Gm9040*	ENSMUSG00000112814	23.96527627	-0.002062252	0.072978	0.85667	0.999804	668198	6:44842621 (+455096)	Not_Significant
CDC5L	*Gm9044*	ENSMUSG00000112419	23.61523503	-0.003165299	0.073039	0.783546	0.999804	668203	6:44842621 (+455096)	Not_Significant
CDC5L	*Gm9045*	ENSMUSG00000112027	75.16987698	-0.000361084	0.070971	0.984361	0.999804	668205	6:44842621 (+455096)	Not_Significant
CDC5L	*Gm9049*	ENSMUSG00000112919	75.51991822	0.000662514	0.070978	0.974284	0.999804	668216	6:44842621 (+455096)	Not_Significant
DLX6	*Dlx6*	ENSMUSG00000029754	797.0151222	0.002215813	0.065566	0.942113	0.999804	13396	7:96585031 (-420517)	Not_Significant
FAR2	*Far2*	ENSMUSG00000030303	86.78406396	-0.005608741	0.070696	0.791145	0.999804	330450	12:28354259 (-794941)	Not_Significant
GGACT	*Ggact*	ENSMUSG00000041625	129.9349817	-0.006032021	0.07138	0.75575	0.999804	223267	13:100322513 (+261484)	Not_Significant
KIF6	*Kif6*	ENSMUSG00000023999	20.82067144	-0.003901	0.073185	0.720197	0.999804	319991	6:39568846 (+156559)	Not_Significant
LLPH	*Llph*	ENSMUSG00000020224	887.9858722	0.023804039	0.061032	0.518815	0.999804	66225	12:65980466 (+150302)	Not_Significant
MEIS1	*Meis1*	ENSMUSG00000020160	377.3524187	0.008156528	0.068657	0.760873	0.999804	17268	2:65750729 (-684671)	Not_Significant
MRPS30	*Mrps30*	ENSMUSG00000021731	522.199464	-0.010272245	0.064289	0.793365	0.999804	59054	5:44441601 (-367324)	Not_Significant
MTX2	*Mtx2*	ENSMUSG00000027099	1014.511223	0.020592201	0.05928	0.579725	0.999804	53375	2:176247091 (-22328)	Not_Significant
PCCA	*Pcca*	ENSMUSG00000041650	707.07619	0.001601378	0.064105	0.96073	0.999804	110821	13:100322513 (+233430)	Not_Significant
RNF146	*Rnf146*	ENSMUSG00000038876	312.8025997	-0.004421824	0.066245	0.902207	0.999804	68031	6:127134977 (-131633)	Not_Significant
RNF32	*Rnf32*	ENSMUSG00000029130	46.08447159	0.003806578	0.07218	0.812885	0.999804	56874	7:156385384 (-255400)	Not_Significant
SPRED2	*Spred2*	ENSMUSG00000045671	1162.568566	-0.019583022	0.064551	0.5651	0.999804	114716	2:65750729 (-318552)	Not_Significant
TBL1XR1	*Tbl1xr1*	ENSMUSG00000027630	2056.552828	0.021819079	0.060755	0.55062	0.999804	81004	3:177593205 (-396727)	Not_Significant
VEPH1	*Veph1*	ENSMUSG00000027831	374.9499256	0.003741856	0.07341	0.69461	0.999804	72789	3:157756785 (-253358)	Not_Significant
WARS2	*Wars2*	ENSMUSG00000004233	733.0412549	0.009131068	0.064206	0.779076	0.999804	70560	1:119139747 (+924)	Not_Significant
DLX5	*Dlx5*	ENSMUSG00000029755	1527.414073	-0.024525564	0.063471	0.500126	0.999804	13395	NA	Not_Significant
NUDCD1	*Nudcd1*	ENSMUSG00000038736	747.9382989	0.000911016	0.063252	0.969318	0.999804	67429	NA	Not_Significant
CYP26A1	*Cyp26a1*	ENSMUSG00000024987	2.967923358	0.003138584	0.073893	0.362401	NA	13082	10:93190051 (+116161)	NA
FERMT1	*Fermt1*	ENSMUSG00000027356	0.321896993	-3.34E-05	0.073859	0.998392	NA	241639	20:6608452 (-484908)	NA
HAO1	*Hao1*	ENSMUSG00000027261	0.327200715	-0.00086939	0.073873	0.651806	NA	15112	20:7123941 (+816533)	NA
KCNJ16	*Kcnj16*	ENSMUSG00000051497	0.486321104	0.0005003	0.073854	0.784697	NA	16517	17:70079183 (-25671)	NA
KCNMB2	*Kcnmb2*	ENSMUSG00000037610	3.880344161	0.002002321	0.073733	0.698606	NA	72413	3:177593205 (-965495)	NA
TMEM74	*Tmem74*	ENSMUSG00000054409	3.349285989	0.002479892	0.073778	0.60483	NA	239408	8:108534556 (+253059)	NA
HAO2	*Hao2*	ENSMUSG00000027870	1.024267482	9.54E-05	0.073838	0.964818	NA	56185	NA	NA
HSD3B1	*Hsd3b1*	ENSMUSG00000027871	0.697066767	0.000781543	0.073861	0.631823	NA	15492	NA	NA

Supplementary Table 7, Testing of GWAS lead SNPs in craniosynostosis cohorts.

The cohorts (sagittal, coronal, metopic, lambdiodal synostosis) comprise patient-parent trios. P-values were obtained using the TDT test (upper-tailed chi squared).

					Sagittal (n = 189)					Coronal (n = 276)					Metopic (n = 186)					Lambdoidal (n = 51)				
CHR	SNP	BP	A1	A2	T	U	OR	CHISQ	P	T	U	OR	CHISQ	P	T	U	OR	CHISQ	P	T	U	OR	CHISQ	P
1	rs3936018	119139747	C	T	22	23	0.9565	0.02222	0.8815	Not tested	Not tested	Not tested	Not tested	Not tested	Not tested	Not tested	Not tested	Not tested	Not tested	Not tested	Not tested	Not tested	Not tested	Not tested
2	rs2009778	65750729	C	T	29	29	1	0	1	49	40	1.225	0.9101	0.3401	24	36	0.6667	2.4	0.1213	6	9	0.6667	0.6	0.4386
2	rs6739488	118432725	A	G	38	34	1.118	0.2222	0.6374	50	36	1.389	2.279	0.1311	38	21	1.81	4.898	0.02688	4	9	0.4444	1.923	0.1655
2	rs17479393	144895720	T	A	22	21	1.048	0.02326	0.8788	24	40	0.6	4	0.0455	23	20	1.15	0.2093	0.6473	7	4	1.75	0.8182	0.3657
2	rs970797	176247091	T	G	29	34	0.8529	0.3968	0.5287	48	43	1.116	0.2747	0.6002	32	35	0.9143	0.1343	0.714	4	6	0.6667	0.4	0.5271
3	rs7626244	157756785	C	A	33	32	1.031	0.01538	0.9013	37	52	0.7115	2.528	0.1118	29	24	1.208	0.4717	0.4922	11	8	1.375	0.4737	0.4913
3	rs35614773	177593205	G	T	28	29	0.9655	0.01754	0.8946	39	46	0.8478	0.5765	0.4477	26	19	1.368	1.089	0.2967	6	8	0.75	0.2857	0.593
5	rs1351637	44441601	G	A	24	16	1.5	1.6	0.2059	31	25	1.24	0.6429	0.4227	20	14	1.429	1.059	0.3035	8	5	1.6	0.6923	0.4054
5	rs3822730	171747250	G	C	32	29	1.103	0.1475	0.7009	60	34	1.765	7.191	0.007325	33	31	1.065	0.0625	0.8026	9	11	0.8182	0.2	0.6547
6	rs4714260	39568846	C	T	34	27	1.259	0.8033	0.3701	50	39	1.282	1.36	0.2436	25	31	0.8065	0.6429	0.4227	6	11	0.5455	1.471	0.2253
6	rs3799970	44842621	A	T	25	20	1.25	0.5556	0.4561	26	29	0.8966	0.1636	0.6858	27	16	1.688	2.814	0.09345	5	4	1.25	0.1111	0.7389
6	rs9491697	127134977	G	A	31	37	0.8378	0.5294	0.4669	45	42	1.071	0.1034	0.7477	30	26	1.154	0.2857	0.593	9	6	1.5	0.6	0.4386
6	rs296418	133334925	G	A	21	30	0.7	1.588	0.2076	32	45	0.7111	2.195	0.1385	23	24	0.9583	0.02128	0.884	7	12	0.5833	1.316	0.2513
7	rs148673350	33219926	T	C	**8**	**33**	**0.2424**	**15.24**	**9.45E-05**	43	36	1.194	0.6203	0.431	34	19	1.789	4.245	0.03936	10	10	1	0	1
7	rs1581525	121228465	A	G	25	35	0.7143	1.667	0.1967	31	39	0.7949	0.9143	0.339	28	31	0.9032	0.1525	0.6961	6	8	0.75	0.2857	0.593
7	rs147676525	156385384	G	A	1	4	0.25	1.8	0.1797	5	3	1.667	0.5	0.4795	10	1	10	7.364	0.006656	0	0	NA	NA	NA
8	rs7813717	108534556	A	G	27	39	0.6923	2.182	0.1396	48	49	0.9796	0.01031	0.9191	35	32	1.094	0.1343	0.714	9	6	1.5	0.6	0.4386
9	rs10120728	108615575	C	T	25	34	0.7353	1.373	0.2413	37	40	0.925	0.1169	0.7324	27	24	1.125	0.1765	0.6744	7	9	0.7778	0.25	0.6171
10	rs7920484	93190051	C	A	33	27	1.222	0.6	0.4386	47	47	1	0	1	23	39	0.5897	4.129	0.04215	7	12	0.5833	1.316	0.2513
12	rs61920200	28085972	A	G	30	27	1.111	0.1579	0.6911	36	52	0.6923	2.909	0.08808	30	23	1.304	0.9245	0.3363	6	10	0.6	1	0.3173
12	rs10843158	28354259	T	C	29	24	1.208	0.4717	0.4922	39	46	0.8478	0.5765	0.4477	26	21	1.238	0.5319	0.4658	9	7	1.286	0.25	0.6171
12	rs151174669	65980466	T	C	21	27	0.7778	0.75	0.3865	40	28	1.429	2.118	0.1456	10	26	0.3846	7.111	0.007661	5	10	0.5	1.667	0.1967
12	rs11609649	85182714	A	G	25	21	1.19	0.3478	0.5553	26	24	1.083	0.08	0.7773	22	14	1.571	1.778	0.1824	1	6	0.1667	3.571	0.05878
13	rs1034266	100322513	G	C	**41**	**16**	**2.562**	**10.96**	**0.0009285**	48	34	1.412	2.39	0.1221	29	30	0.9667	0.01695	0.8964	6	10	0.6	1	0.3173
15	rs4842918	83868247	C	T	23	18	1.278	0.6098	0.4349	35	46	0.7609	1.494	0.2216	20	28	0.7143	1.333	0.2482	3	7	0.4286	1.6	0.2059
17	rs12940346	70079183	C	T	25	32	0.7812	0.8596	0.3538	43	35	1.229	0.8205	0.365	31	19	1.632	2.88	0.08969	11	6	1.833	1.471	0.2253
20	rs1321454	6608452	C	T	22	24	0.9167	0.08696	0.7681	42	38	1.105	0.2	0.6547	31	37	0.8378	0.5294	0.4669	10	6	1.667	1	0.3173
20	rs6054748	7123941	A	G	**61**	**11**	**5.545**	**34.72**	**3.80E-09**	58	36	1.611	5.149	0.02326	26	26	1	0	1	16	4	4	7.2	0.00729

Supplementary Table 8, Human annotations of abnormal neurocranium and brain morphology for GWAS candidate genes.

Lead SNP from GWAS	Probable Candidate Gene	Relevant Neurocranium Phenotypes from GWAS	Ref DOI	Relevant Syndromes and Disorders with Neurocranium Phnenotype	Association with Neurocranium Dysmorphology
rs3936018	*TBX15*	White et al. (2021): forehead morphology. (PMID:33288918)	https://doi.org/10.1038/s41588-020-00741-7	Cousin Syndrome (OMIM:260660).	Macrocephaly; Abnormality of the skull base; Frontal bossing.<br>https://hpo.jax.org/app/browse/gene/6913
rs2009778	*MEIS1*	White et al. (2021): lateral forehead and supraorbital ridge morphology. (PMID:33288918)	https://doi.org/10.1038/s41588-020-00741-7	Cleft Palate, Cardiac Defects, And Mental Retardation (OMIM:600987).<br>15q14 Microdeletion Syndrome (ORPHA:261190).	Narrow forehead; Biparietal narrowing; Microcephaly; Large forehead.<br>https://hpo.jax.org/app/browse/gene/4212
rs6739488	*EN1*	White et al. (2021): forehead morphology. (PMID:33288918)	https://doi.org/10.1038/s41588-020-00741-7	Endove Syndrome, Limb-brain Type (OMIM:619218).	Microcephaly.<br>https://hpo.jax.org/app/browse/gene/2019
rs17479393	*ZEB2*	White et al. (2021): upper face and forehead morphology. (PMID:33288918)	https://doi.org/10.1038/s41588-020-00741-7	Mowat-Wilson Syndrome (OMIM:235730).	Microcephaly; Abnormal posterior cranial fossa morphology; Frontal bossing; Right unicoronal synostosis.<br>https://hpo.jax.org/app/browse/gene/9839
rs970797	*HOXD10/13*	White et al. (2021): global face morphology, including forehead region. (PMID:33288918)	https://doi.org/10.1038/s41588-020-00741-7	Brachydactyly Type E (ORPHA:93387).	Frontal bossing; Macrocephaly.<br>https://hpo.jax.org/app/browse/gene/3239
rs7626244	*SHOX2*	White et al. (2021): forehead morphology. (PMID:33288918)	https://doi.org/10.1038/s41588-020-00741-7		
rs35614773	*TBL1XR1*			Pierpont Syndrome (ORPHA:487825).	Microcephaly; High forehead; Brachycephaly.<br>https://hpo.jax.org/app/browse/gene/79718
rs1351637	*FGF10*	White et al. (2021): lateral forehead and temple region morphology. (PMID:33288918)	https://doi.org/10.1038/s41588-020-00741-7	Lacrimoauriculodentodigital Syndrome (OMIM:149730).	Broad forehead.<br>https://hpo.jax.org/app/browse/gene/2255
rs3822730	*FGF18*	White et al. (2021): central forehead and supraorbital ridge morphology. (PMID:33288918)<br>Shadrin et al. (2021): brain morphology. (PMID:34560273)	https://doi.org/10.1038/s41588-020-00741-7<br>https://doi.org/10.1016/j.neuroimage.2021.118603		
rs4714260	*KIF6*	White et al. (2021): forehead morphology. (PMID:33288918)<br>Shadrin et al. (2021): brain morphology. (PMID:34560273)<br>van der Meer et al. (2020): brain morphology. (PMID:32665545)	https://doi.org/10.1038/s41588-020-00741-7<br>https://doi.org/10.1016/j.neuroimage.2021.118603<br>https://doi.org/10.1038/s41467-020-17368-1	Isolated case report (Konjikusic et al., 2018 (PMID:30475797; https://doi.org/10.1371/journal.pgen.1007817).	Macrocephaly.
rs3799970	*RUNX2*	van der Meer et al. (2020): brain morphology. (PMID:32665545)	https://doi.org/10.1038/s41467-020-17368-1	Cleidocranial Dysplasia (OMIM:119600).	Thickened calvaria; Broad forehead; Macrocephaly; Sloping forehead; Parietal bossing; Persistent open anterior fontanelle; Frontal bossing; Wormian bones; Large fontanelles; Brachycephaly.<br>https://hpo.jax.org/app/browse/gene/860
rs9491697	*RSPO3*	Grasby et al. (2020): brain morphology. (PMID:32193296)	https://doi.org/10.1126/science.aay6690		
rs296418	*EYA4*	White et al. (2021): forehead morphology. (PMID:33288918)<br>Shadrin et al. (2021): brain morphology. (PMID:34560273)	https://doi.org/10.1038/s41588-020-00741-7<br>https://doi.org/10.1016/j.neuroimage.2021.118603		
rs148673350	*BBS9*	Justice et al. (2012): sagittal synostosis; metopic synostosis. (PMID:23160099)<br>van der Meer et al. (2020): brain morphology. (PMID:32665545)	https://doi.org/10.1038/ng.2463<br>https://doi.org/10.1038/s41467-020-17368-1	Bardet-Biedl Syndrome (ORPHA:110).	
rs202055590	*DLX5*			Split-hand/foot Malformation 1 With Sensorineural Hearing Loss (OMIM:220600).	Frontal bossing.<br>https://www.omim.org/entry/220600
rs1581525	*WNT16*	White et al. (2021): forehead morphology. (PMID:33288918)<br>Zhao et al. (2019): brain morphology. (PMID:31676860)	https://doi.org/10.1038/s41588-020-00741-7<br>https://doi.org/10.1038/s41588-019-0516-6		
rs147676525	*SHH*	Shadrin et al. (2021): brain morphology. (PMID:34560273)	https://doi.org/10.1016/j.neuroimage.2021.118603	Holoprosencephaly (ORPHA:220386; ORPHA:93925; ORPHA:93924; ORPHA:280200).	Megalencephaly; Macrocephaly; Microcephaly.<br>https://hpo.jax.org/app/browse/gene/6469
rs7813717<br>rs10120728	*RSPO2*<br>*ELP1*	van der Meer et al. (2020): brain morphology. (PMID:32665545)	https://doi.org/10.1038/s41467-020-17368-1	Humerofemoral Hypoplasia with Radiotibial Ray Deficiency (OMIM:618022).	Prominent glabella.<br>https://hpo.jax.org/app/browse/gene/340419
rs7920484	*CEP55*				
rs61920200<br>rs10843158	*PTHLH*	White et al. (2021): forehead morphology. (PMID:33288918)<br>Shadrin et al. (2021): brain morphology. (PMID:34560273)	https://doi.org/10.1038/s41588-020-00741-7<br>https://doi.org/10.1016/j.neuroimage.2021.118603	Brachydactyly Type E (ORPHA:93387).	Frontal bossing; Macrocephaly.<br>https://hpo.jax.org/app/browse/gene/5744
rs151174669	*HMGA2*	White et al. (2021): forehead morphology. (PMID:33288918)<br>Taal et al. (2012): head circumference. (PMID:22504419)<br>Bonfonte et al. (2021): supraorbital ridge morphology. (PMID:33547071)<br>Yang et al. (2019): head circumference. (PMID:31681408)<br>Shadrin et al. (2021): brain morphology. (PMID:34560273)<br>Grasby et al. (2020): brain morphology. (PMID:32193296)	https://doi.org/10.1038/s41588-020-00741-7<br>https://doi.org/10.1038/ng.2238<br>https://doi.org/10.1126/sciadv.abc6160<br>https://doi.org/10.3389/fgene.2019.00947<br>https://doi.org/10.1016/j.neuroimage.2021.118603<br>https://doi.org/10.1126/science.aay6690	Silver-Russell Syndrome 5 (OMIM:618908).<br>12q14 Microdeletion Syndrome (ORPHA:94063).	Microcephaly; Prominent forehead; Frontal bossing.<br>https://hpo.jax.org/app/browse/gene/8091
rs11609649	*ALX1*	White et al. (2021): global face morphology, including forehead region. (PMID:33288918)<br>Shadrin et al. (2021): brain morphology. (PMID:34560273)	https://doi.org/10.1038/s41588-020-00741-7<br>https://doi.org/10.1016/j.neuroimage.2021.118603	Frontonasal Dysplasia 3 (OMIM:613456).	Hypoplasia of the frontal bone; Brachycephaly; Prominent glabella.<br>https://hpo.jax.org/app/browse/gene/8092
rs1034266	*ZIC2*	White et al. (2021): forehead morphology. (PMID:33288918)	https://doi.org/10.1038/s41588-020-00741-7	Holoprosencephaly (ORPHA:220386; ORPHA:93925; ORPHA:93924; ORPHA:280200).	Megalencephaly; Macrocephaly; Microcephaly; Broad forehead; Sloping forehead; High forehead; Narrow forehead.<br>https://hpo.jax.org/app/browse/gene/7546
rs1380208	*Gene Desert*				
rs4842918	*ADAMTSL3*	Shadrin et al. (2021): brain morphology. (PMID:34560273)	https://doi.org/10.1016/j.neuroimage.2021.118603		
rs12940346	*KCNJ2*			Andersen Cardiodysrhythmic Periodic Paralysis (OMIM:170390).<br>Andersen-Tawil Syndrome (ORPHA:37553).	Prominent frontal sinuses; Broad forehead; Microcephaly; Scaphocephaly.<br>https://hpo.jax.org/app/browse/gene/3759
rs1321454<br>rs6054748	*BMP2*	Justice et al. (2012): sagittal synostosis. (PMID:23160099)<br>Shadrin et al. (2021): brain morphology. (PMID:34560273)	https://doi.org/10.1038/ng.2463<br>https://doi.org/10.1016/j.neuroimage.2021.118603	20p12.3 Microdeletion Syndrome (ORPHA:261295).<br>Short Stature, Facial Dysmorphism, and Skeletal Anomalies with or without Cardiac Anomalies (OMIM:617877).	Broad forehead; Narrow forehead; Macrocephaly.<br>https://hpo.jax.org/app/browse/gene/650

Supplementary Table 9, Mouse annotations of abnormal neurocranium morphology for GWAS candidate genes.

Lead SNP from GWAS	Probable Candidate Gene	Neurocranium and Brain Phenotypes in Mouse Mutants	Neurocranium and Brain Expression in Mouse	Notes	MGI URL
rs3936018	*TBX15*	Kuijper et al. (2005): abnormal basioccipital bone morphology; decreased cranium height; decreased cranium width. (PMID:15728667)<br>Curry (1959): shortened head. (PMID:13654621)<br>Singh et al. (2005): abnormal neurocranium morphology: abnormal occipital bone morphology; abnormal temporal bone morphology; small basioccipital bone. (PMID: 15652702)	From MGI: Brain (forebrain, hindbrain, midbrain); Cranial Base (basioccipital cartilage condensation).	Another potentially relevant gene at this locus is *WARS2* .<br>TBX15 also associated shows neurocranium expression in Zebrafish. (Begemann et al., 2002). (PMID:12175500)	Link
rs2009778	*MEIS1*	Hisa et al. (2004): abnormal brain morphology. (PMID:14713950)	From MGI: Brain (forebrain, hindbrain, midbrain, cerebral cortex gray matter).	*MEIS1* also associated with craniofacial morphology in Zebrafish (Melvin et al., 2013). (PMID:23559552)<br>Another potentially relevant gene at this locus is *SPRED2* .	Link
rs6739488	*EN1*	Broccoli et al. (1999): abnormal brain morphology; abnormal cerebellum morphology. (PMID:10490025)<br>Panhuysen et al. (2004): abnormal cerebellum morphology; abnormal hindbrain morphology. (PMID:15121182)<br>Loomis et al. (1996): abnormal brain morphology; abnormal cerebellum morphology. (PMID:8684466)<br>Cygan et al. (1997): abnormal brain morphology. (PMID:9362463)<br>Sgaier et al. (2007): abnormal cerebellum morphology. (PMID:17537797)<br>Hanks et al. (1998): abnormal brain morphology; abnormal cerebellum morphology. (PMID:9778510)<br>Deckelbaum et al. (2006): wide cranial sutures; hypoplastic and undermineralized cranial vault bones). (PMID:16319118)	From MGI: Brain (forebrain, hindbrain, midbrain).<br>Augustine et al. (1995): Brain (developing neural tube). (PMID:7482351)<br>Tran et al. (2010): Cranial vault (frontal bone, parietal bone). (PMID:20980404)<br>Deckelbaum et al. (2006): Cranial Vault (frontal bone, parietal bone, interparietal bone, all cranial sutures). (PMID:16319118)		Link
rs17479393	*ZEB2*	Van de Putte et al. (2003): abnormal cranial neural crest cell migration; abnormal neural crest cell delamination; open neural tube. (PMID:12522767)<br>Miyoshi et al. (2006): open neural tube; abnormal neural plate morphology. (PMID:16598713)	From MGI: Brain (forebrain, hindbrain, midbrain, cerebral cortex gray matter).<br>Bassez et al. (2004): Brain (developing neural tube). (PMID:15006694)		Link
rs970797	*HOXD10*		From MGI: Brain (forebrain, hindbrain).	Other potentially relevant genes at this locus include *EVX2* , *HOXD11* , *HOXD12* , *HOXD13* , and *MTX2* .	Link
rs7626244	*SHOX2*		From MGI: Brain (forebrain, hindbrain, midbrain, cerebral cortex gray matter).	Other potentially relevant genes at this locus include *VEPH1* , *PTX3* and *RSRC1* .	Link
rs35614773	*TBL1XR1*		From MGI: Brain (forebrain, hindbrain, midbrain, cerebral cortex gray matter).		Link
rs1351637	*FGF10*		From MGI: Brain (forebrain, hindbrain, midbrain, cerebral cortex grey matter, cerebellum gray matter); Cranial vault (coronal suture).<br>Veistinen et al. (2009): Cranial vault (developing frontal bone). (PMID:19205045)		
rs3822730	*FGF18*	Liu et al. (2002): abnormal cranial suture morphology; abnormal craniofacial bone morphology; small neurocranium. (PMID:11937493)<br>Ohbayashi et al. (2002): delayed cranial suture closure. (PMID:11937494)<br>Hung et al. (2016): altered size and shape of the neurocranium. (PMID:26794256)	From MGI: Brain (forebrain, hindbrain, midbrain, cerebral cortex gray matter); Cranial base; Cranial Vault (frontal bone, parietal bone, coronal suture, sagittal suture, metopic suture).		Link
rs4714260	*KIF6*	Konjikusic et al. (2018): domed cranium; increased brain size. (PMID:30475797)	From MGI: Brain (forebrain, hindbrain, midbrain, cerebral cortex gray matter).	Another potentially relevant gene at this locus is *DAAM2* .	Link
rs3799970	*RUNX2*	Lou et al. (2009): abnormal neurocranium morphology; abnormal cranial suture morphology; small basisphenoid bone: wide cranial sutures (PMID:19028669)<br>Okura et al. (2014): abnormal neurocranium morphology; absent occipital bone; delayed sagittal suture closure. (PMID:25244033)<br>Komori et al. (1997): abnormal neurocranium morphology; abnormal interparietal bone morphology; abnormal parietal bone morphology: abnormal supraoccipital bone morphology. (PMID:9182763)<br>Kugimiya et al. (2007): abnormal neurocranium morphology; delayed fontanelle closure. (PMID:17786208)<br>Xiao et al. (2004): abnormal cranium morphology; absent occipital bone. (PMID:15007057)<br>Otto et al. (1997): abnormal cranium morphology; abnormal fontanelle morphology; interparietal bone hypoplasia: wide cranial sutures. (PMID:9182764)<br>Hesse et al. (2010): abnormal fontanelle morphology. (PMID:21173110)	From MGI: Brain (forebrain, hindbrain, midbrain, cerebral cortex gray matter); Chondrocranium (basioccipital bone, basisphenoid bone, exoccipital bone); Cranial vault (frontal bone, parietal bone; interparietal bone, cranial sutures, fontanelle).		Link
rs9491697	*RSPO3*		From MGI: Brain (forebrain, hindbrain, midbrain, cerebral cortex gray matter).	*RSPO3* also involved in craniofacial development in zebrafish (Alhazmi et al., 2021). *(PMID:33712657)*	Link
rs296418	*EYA4*		From MGI: Brain (forebrain, hindbrain, midbrain, cerebral cortex gray matter); Chondrocranium<br>Borsani et al. (1999): Chondrocranium (basisphenoid). (PMID:9887327)		Link
rs148673350	*BBS9*		From MGI: Brain (forebrain, hindbrain, midbrain, cerebral cortex gray matter).<br>Barda et al. (2018): Cranial vault (interfrontal suture). (PMID:29674126)	*BBS9* also involved in brain development in zebrafish (Veleri et al., 2012). *(PMID:22479622)*	Link
rs202055590	*DLX5*	Beverdam et al. (2002): abnormal alisphenoid bone morphology; abnormal basicranium morphology; abnormal basisphenoid bone morphology; abnormal frontal bone morphology; abnormal interparietal bone morphology; abnormal temporal squamous morphology: exencephaly. (PMID:12434331)<br>Depew et al. (1999): abnormal alisphenoid bone morphology; abnormal cranium morphology; abnormal neurocranium morphology; abnormal occipital bone morphology; abnormal temporal squamous morphology; exencephaly: small interparietal bone. (PMID:10433912)<br>Acampora et al. (1999): abnormal alisphenoid bone morphology; abnormal basioccipital bone morphology; abnormal cranium morphology; delayed cranial suture closure; ectopic cranial bone; exencephaly; wide cranial sutures; Wormian bones. (PMID:10433909)	From MGI: Brain (forebrain, hindbrain, midbrain, cerebral cortex gray matter); Chondrocranium; Cranial vault (frontal bone).	Another relevant gene at this locus is *DLX6* .	Link
rs1581525	*WNT16*		From MGI: Brain (forebrain, hindbrain, midbrain, cerebral cortex gray matter).<br>Jiang et al. (2014): Cranial vault (bones prior to mineralization). (PMID:24037946)	Other potentially relevant genes at this locus include *CPED1* and *FAM3C* .	Link
rs147676525	*SHH*	Niedermaier et al. (2005): abnormal cranium morphology; abnormal frontal bone morphology; abnormal neurocranium morphology: thin interparietal bone. (PMID:15841179)<br>Selby et al. (1993): microcephaly. (PMID:8270769)<br>Chan et al. (2009): cerebellum hypoplasia. (PMID:19287388)<br>Dassule et al. (2000): abnormal cranium morphology. (PMID:11044393)<br>Dakubo et al. (2003): microcephaly. (PMID:12756179)<br>Corrales et al. (2004): abnormal cerebellum morphology. (PMID:15496441)<br>Chiang et al. (1996): abnormal forebrain morphology; decreased brain size. (PMID:8837770)<br>Wu et al. (2005): decreased brain size; microcephaly. (PMID:20159447)<br>Lewis et al. (2004): abnormal cerebellum morphology; small cerebellum. (PMID:15183722)<br>Huang et al. (2007): abnormal neurocranium morphology; abnormal telencephalon morphology; increased forebrain size. (PMID:17468181)	From MGI: Brain (forebrain, hindbrain, midbrain, cerebral cortex gray matter).<br>Kim et al. (1998): Cranial vault (metopic suture; sagittal suture). (PMID:9477322)<br>Lenton et al. (2011): Cranial vault (parietal bone). (PMID:21557453)<br>Nie et al. (2005): Chondrocranium (basioccipital, basisphenoid, sphenooccipital synchondrosis, intersphenoid synchondrosis). (PMID:16145660)		Link
rs7813717	*RSPO2*	Yamada et al. (2009): abnormal temporal squamous morphology. (PMID:19233133)	From MGI: Brain (forebrain, hindbrain, midbrain, cerebral cortex gray matter).	*RSPO2* also involved in craniofacial development in zebrafish (Alhazmi et al., 2021). *(PMID:33712657)*	Link
rs10120728	*ELP1*	Chen et al. (2009): abnormal brain development. (PMID:9015235)<br>Dietrich et al. (2011): abnormal brain development; abnormal forebrain development; abnormal telencephalon development. (PMID:22046433)	From MGI: Brain (forebrain, hindbrain, midbrain, cerebral cortex gray matter).	Other potentially relevant genes at this locus include *ACTL7B* , *ACTL7A* , *ABITRAM* , *CTNNAL1* , and *TMEM245* .	Link
rs7920484	*CEP55*	Tedeschi et al. (2020): abnormal cerebral cortex morphology; abnormal cranium morphology; decreased brain size: microcephaly: thin cerebral cortex. (PMID:22046433)	From MGI: Brain (forebrain, hindbrain, midbrain, cerebral cortex gray matter).	Other potentially relevant genes at this locus include *HHEX* and *CYP26A1* .	Link
rs61920200<br>rs10843158	*PTHLH*	He et al. (2001): domed cranium. (PMID:11316774)<br>Amizuka et al. (2004: domed cranium. (PMID:14751559)<br>Suda et al. (2001): domed cranium. (PMID:11760831)<br>Karaplis et al. (1994): domed cranium. (PMID:8314082)<br>Schipani et al. (1997): domed cranium. (PMID:9391087)<br>Miao et al. (2008): decreased brain size. (PMID:19091948)<br>Ishii-Suzuki et al. (1999): abnormal cranial base synchondroses. (PMID:10409817)	From MGI: Brain (forebrain, hindbrain, midbrain, cerebral cortex gray matter); Chondrocranium (synchondroses).	*PTHLH* also shows evidence of craniofacial expression in zebrafish (Yan et al., 2012; Hoyle et al., 2022) and chicks (Abzhanov et al. 2007). *(PMID:17670790 ,22761277,34919126)*	Link
rs151174669	*HMGA2*	Benson and Chada (1994): abnormally large brain size. (PMID:7958830)<br>Zhou et al. (1995): shortened head (brachycephaly). (PMID:7651535)<br>Lee et al. (2022): altered cranial vault shape. (PMID:34878116)	From MGI: Brain (forebrain, hindbrain, midbrain).	Another potentially relevant gene at this locus is *GRIP1* .<br>*HMGA2* also associated with craniofacial dysmorphology in rabbits (Carneiro et al., 2017). *(PMID:27986804)*	Link
rs11609649	*ALX1*	Zhao et al. (1996): abnormal forebrain morphology; absent interparietal bone; absent neurocranium; absent presphenoid bone; decreased cranium length; meroanencephaly; small frontal bone; small parietal bone; small supraoccipital bone: small temporal squamous: shortened head. (PMID:8673125)	From MGI: Brain (forebrain).		Link
rs1034266	*ZIC2*	Dykes et al. (2018): exencephaly. (PMID:29992973)<br>Warr et al. (2008): abnormal forebrain development; exencephaly. (PMID:18617531)<br>Elms et al. (2003): decreased neural crest cell number. (PMID:14651926)<br>Hatayama et al. (2011): abnormal cerebral hemisphere morphology; decreased brain size; thin cerebral cortex. (PMID:22355535)<br>Nagai et al. (2000): abnormal cerebral cortex morphology; abnormal forebrain development; abnormal telencephalon development: exencephaly: microcephaly. (PMID:10677508)	From MGI: Brain (forebrain, hindbrain, midbrain, cerebral cortex gray matter).<br>Bellchamber et al. (2021): Brain (borders of the neural plate during crest cell formation). (PMID:34638777)	Another potentially relevant gene at this locus is *ZIC5* .<br>*ZIC2* also involved in craniofacial development in zebrafish (Teslaa et al., 2013). *(PMID:23665173)*	Link
rs1380208				This region is a gene desert	
rs4842918	*ADAMTSL3*		From MGI: Brain (forebrain, hindbrain, midbrain, cerebral cortex gray matter).	Other potentially relevant genes at this locus include *SH3GL3* and *GOLGA6L4* .<br>None of the genes at this locus are strong candidates.	Link
rs12940346	*KCNJ2*	Belus et al. (2018): wide sagittal sutures; enlarged fontanelles; small squamosal bone; small basisphenoid bone. (PMID:29571612)	From MGI: Brain (forebrain, hindbrain, midbrain, cerebral cortex gray matter).<br>Adams et al. (2016): Brain (neural folds). (PMID:26864374)	There is also evidence that this region harbors regulatory elements for *SOX9* (Castori et al., 2016). (PMID:26663529)	Link
rs1321454<br>rs6054748	*BMP2*		From MGI: Brain (forebrain, hindbrain, midbrain, cerebral cortex gray matter); Chondrocranium (sphenoid bone, basioccipital bone/cartilage, synchondroses).<br>Kim et al. (1998) Cranial vault (osteogenic fonts of sagittal suture, parietal bone). (PMID:9477322)	Multipeak locus	Link

Supplementary Table 10, Table of reference data anchors.
Anchor reference samples were derived from joint 1000G and HDGP datasets based on a K:6 ADMIXTURE model (Methods), followed by K-means clustering, and filtering for samples with higher-than-average main ancestry component per cluster.

Anchor Cluster	Population	Number of Samples
0	Africa	545
1	Europe	613
2	East-Asia	601
3	South-Asia	369
4	America	105
5	Oceania	13

Supplementary Table 11, List of GTEx8 tissues used for eQTL colocalization analysis

Tissue label	
Brain - Amygdala	Brain
Brain - Caudate (basal ganglia)	
Brain - Nucleus accumbens (basal ganglia)	
Brain - Putamen (basal ganglia)	
Brain - Cerebellum	
Brain - Cerebellar Hemisphere	
Brain - Anterior cingulate cortex (BA24)	
Brain - Cortex	
Brain - Frontal Cortex (BA9)	
Brain - Hippocampus	
Brain - Hypothalamus	
Brain - Spinal cord (cervical c-1)	
Brain - Substantia nigra	
Cells - Cultured fibroblasts	Skin
Skin - Not Sun Exposed (Suprapubic)	
Skin - Sun Exposed (Lower leg)	
Pituitary	Glands
Thyroid	
Testis	
Muscle - Skeletal	Other
Adipose - Subcutaneous	
Adipose - Visceral (Omentum)	

Supplementary Table 12, Colocalization of GWAS loci and eQTL from 22 tissues in GTEx8.
All tissue-gene combinations with posterior probability for overlap (PP4) ≥ 0.5 are listed per locus.

Lead SNP	Tissue	Gene	PP4
rs3936018	Brain - Substantia nigra	*WARS2*	0.931
rs3936018	Skin - Sun Exposed (Lower leg)	*WARS2*	0.848
rs3936018	Skin - Sun Exposed (Lower leg)	*HSD3B2*	0.857
rs3936018	Thyroid	*WARS2*	0.948
rs3936018	Thyroid	*HAO2*	0.824
rs3936018	Adipose - Visceral (Omentum)	*WARS2*	0.733
rs3936018	Adipose - Visceral (Omentum)	*HSD3B2*	0.837
rs35614773	Cells - Cultured fibroblasts	*TBL1XR1*	0.754
rs3822730	Skin - Sun Exposed (Lower leg)	*FGF18*	0.801
rs4714260	Skin - Not Sun Exposed (Suprapubic)	*DAAM2*	0.978
rs4714260	Skin - Not Sun Exposed (Suprapubic)	*KIF6*	0.811
rs9491697	Cells - Cultured fibroblasts	*RSPO3*	0.929
rs9491697	Adipose - Subcutaneous	*RSPO3*	0.887
rs296418	Testis	*EYA4*	0.929
rs296418	Adipose - Visceral (Omentum)	*EYA4*	0.955
rs148673350	Brain - Caudate (basal ganglia)	*BBS9*	0.549
rs148673350	Cells - Cultured fibroblasts	*BMPER*	0.746
rs148673350	Pituitary	*BBS9*	0.914
rs1581525	Brain - Putamen (basal ganglia)	*WNT16*	0.705
rs7813717	Brain - Nucleus accumbens (basal ganglia)	*EMC2*	0.599
rs7813717	Brain - Putamen (basal ganglia)	*EIF3E*	0.516
rs7813717	Brain - Hippocampus	*EMC2*	0.923
rs7813717	Pituitary	*EIF3E*	0.507
rs7813717	Thyroid	*EIF3E*	0.636
rs7813717	Testis	*NUDCD1*	0.786
rs7920484	Brain - Cerebellar Hemisphere	*CPEB3*	0.536
rs7920484	Skin - Sun Exposed (Lower leg)	*KIF11*	0.611
rs61920200	Skin - Not Sun Exposed (Suprapubic)	*CCDC91*	0.699
rs10843158	Skin - Not Sun Exposed (Suprapubic)	*CCDC91*	0.699
rs1034266	Skin - Not Sun Exposed (Suprapubic)	*ZIC2*	0.943
rs1034266	Skin - Sun Exposed (Lower leg)	*ZIC2*	0.567
rs1034266	Skin - Sun Exposed (Lower leg)	*PCCA*	0.620
rs4842918	Brain - Hypothalamus	*GOLGA6L4*	0.820
rs4842918	Adipose - Visceral (Omentum)	*WHAMM*	0.801
rs12940346	Brain - Cerebellum	*KCNJ16*	0.994
rs12940346	Brain - Cerebellar Hemisphere	*KCNJ16*	0.991
rs12940346	Brain - Hippocampus	*ABCA9*	0.548
rs1321454	Cells - Cultured fibroblasts	*BMP2*	0.692
rs1321454	Adipose - Visceral (Omentum)	*CASC20*	0.510

Supplementary Note: Further details on methods

Estimating the study-wide significance threshold

To combine our GWAS results across multiple segments, we took for each SNP the minimal P-value across the 15 segments (minP). Since correlation between the segments is expected (due to e.g., the hierarchical nature of segments), a Bonferroni correction based on 15 independent tests would be overly stringent. For a more accurate adjustment, we estimated the number of effective independent tests per SNP following Kanai[1] and ran 10,000 genotype-phenotype associations under the null by permutation testing in a way that preserved the correlational structure of our phenotypes. This was repeated for 500 random SNPs and yielding an estimate of 11.44 (± 0.55) independent traits.

To illustrate the robustness of this estimate, we additionally estimated the number of effective traits using several other approaches. First, we repeated the permutation testing for our 30 lead SNPs, yielding an estimate for the number of effective traits of 11.28 (± 0.50). Second, we applied several methods to estimate the number of effective traits from a trait-correlation matrix. We used three methods: 1) Li & Ji[2], 2) Galwey[3], and 3) Li[4] on three different correlation matrices: 1) based on the P-values from the GWAS, 2) based on the Chi-squared statistics from the GWAS, and 3) based on the Spearman genetic correlation matrix (methods). All three correlation matrices yielded highly concordant results across methods (Table 1). While these estimates align, the most conservative estimate of 11.44 was obtained based on 10,000 permutations of 500 random SNPs. We therefore adjusted the genome-wide significance threshold (instead of the P-values themselves) using Bonferroni correction based on 11.44 effective traits, resulting in a study-wide threshold of 4.37e-9 (i.e., 5e-8 / 11.44).

Table 1 The number of effective traits estimated through different methods.

Method	P-values	Chi-squares	Genetic correlation
Li & Ji	11.00	10.00	10.00
Galwey	11.27	10.84	10.98
Li	8.87	8.52	8.57

In short, we tried several approaches to estimate the number of effective traits, thereby taking into account the correlational structure between the phenotypes. We showed robustness of our estimates and used the most conservative estimate to adjust the significance threshold.

References

1. Kanai, M., Tanaka, T. & Okada, Y. Empirical estimation of genome-wide significance thresholds based on the 1000 Genomes Project data set. *J. Hum. Genet.* **61**, 861–866 (2016).
2. Li, J. & Ji, L. Adjusting multiple testing in multilocus analyses using the eigenvalues of a correlation matrix. *Heredity* **95**, 221–227 (2005).
3. Galwey, N. W. A new measure of the effective number of tests, a practical tool for comparing families of non-independent significance tests. *Genet. Epidemiol.* **33**, 559–568 (2009).
4. Li, M.-X., Gui, H.-S., Kwan, J. S. H. & Sham, P. C. GATES: A Rapid and Powerful Gene-Based Association Test Using Extended Simes Procedure. *Am. J. Hum. Genet.* **88**, 283–293 (2011).
