# Supplementary material for: Joint multi-ancestry and admixed GWAS reveals the complex genetics behind human cranial vault shape
Source: Nat Commun. 2023 Nov 16;14:7436. doi: 10.1038/s41467-023-43237-8 (PMC10654897; doi:10.1038/s41467-023-43237-8)
Supplement: Supplementary file 3 — Description of Additional Supplementary Files [file 41467_2023_43237_MOESM3_ESM.pdf]

Description of Additional Supplementary Files

File Name: Supplementary Data 1
Description: **Overview figures of 30 genome-wide significant loci. A**, LocusZoom plots for the segment in which the SNP had its lowest P-value (one-sided chi-square). Points are colored based on linkage disequilibrium (r^2) in the 1000 Genomes Phase 3 EUR population. **B,** -log10(P-values) (one-sided chi-square) across hierarchical cranial vault segments. Genome-wide significant associations ($P < 5e\text{-}8$) are indicated with black circles on the polar dendrogram and segments that significantly replicated in the UK Biobank ($P < 0.0244$) are indicated with '*'. **C,** Latent shapes associated with the most significant segment (top row), and full cranial vault (bottom row). Red and blue represent an outwards and inwards deformation respectively relative to the overall average cranial vault shape.

File Name: Supplementary Data 2
Description: **Comparison of multi-ancestry and European shape associations at the joint set of loci. A**, Shape associated with lead SNPs from the full multi-ancestry GWAS (FULL; n = 6,772) and European subset GWAS (EUR; n = 4,198). Bold rsID labels indicate that the SNP was a lead SNP in the respective GWAS; regular type rsID labels indicate that the SNP was not genome-wide significant but had the lowest P-value among all SNPs within 200kb or in LD ($r^2 > 0.2$). Blue and red indicate inward depression and outward protrusion respectively. **B,** -log10(P-values) (one-sided chi-square) across hierarchical cranial vault segments. Genome-wide significant ($P < 5e\text{-}8$) segments are indicated with a black circle. The strongest association across all segments is indicated with '*'. **C,** LocusCompare plot of minimal P-values across hierarchical segments. Colors indicate LD with the most significant SNP across the FULL and EUR GWAS (purple diamond). If that SNP was not found in the 1000 Genomes dataset, the next most significant SNP was chosen. **D,** LocusZoom plots of minimal P-values across hierarchical segments. Panel legend matches with **C**.
