# Supplementary material for: Joint multi-ancestry and admixed GWAS reveals the complex genetics behind human cranial vault shape
Source: Nat Commun. 2023 Nov 16;14:7436. doi: 10.1038/s41467-023-43237-8 (PMC10654897; doi:10.1038/s41467-023-43237-8)

Supplementary Information
Overview of Genome-Wide Significant Loci
S. Goovaerts et al.

Contents

RS3936018 3
RS2009778 4
RS6739488 5
RS17479393 6
RS970797 7
RS7626244 8
RS35614773 9
RS1351637 10
RS3822730 11
RS4714260 12
RS3799970 13
RS9491697 14
RS296418 15
RS148673350 16
RS202055590 17
RS1581525 18
RS147676525 19
RS7813717 20
RS10120728 21
RS7920484 22
RS61920200 23
RS10843158 24
RS151174669 25
RS11609649 26
RS1034266 27
RS1380208 28
RS4842918 29
RS12940346 30
RS1321454 31
RS6054748 32

See page 2 for captions

Overview figures of 30 genome-wide significant loci. **A**, LocusZoom plots for the segment in which the SNP had its lowest P-value (one-sided chi-square). Points are colored based on linkage disequilibrium (r^2) in the 1000 Genomes Phase 3 EUR population. **B,** -log10(P-values) (one-sided chi-square) across hierarchical cranial vault segments. Genome-wide significant associations (P < 5e-8) are indicated with black circles on the polar dendrogram and segments that significantly replicated in the UK Biobank (P < 0.0244) are indicated with '*'. **C,** Latent shapes associated with the most significant segment (top row), and full cranial vault (bottom row). Red and blue represent an outwards and inwards deformation respectively relative to the overall average cranial vault shape.

See page 2 for captions

rs3936018

A

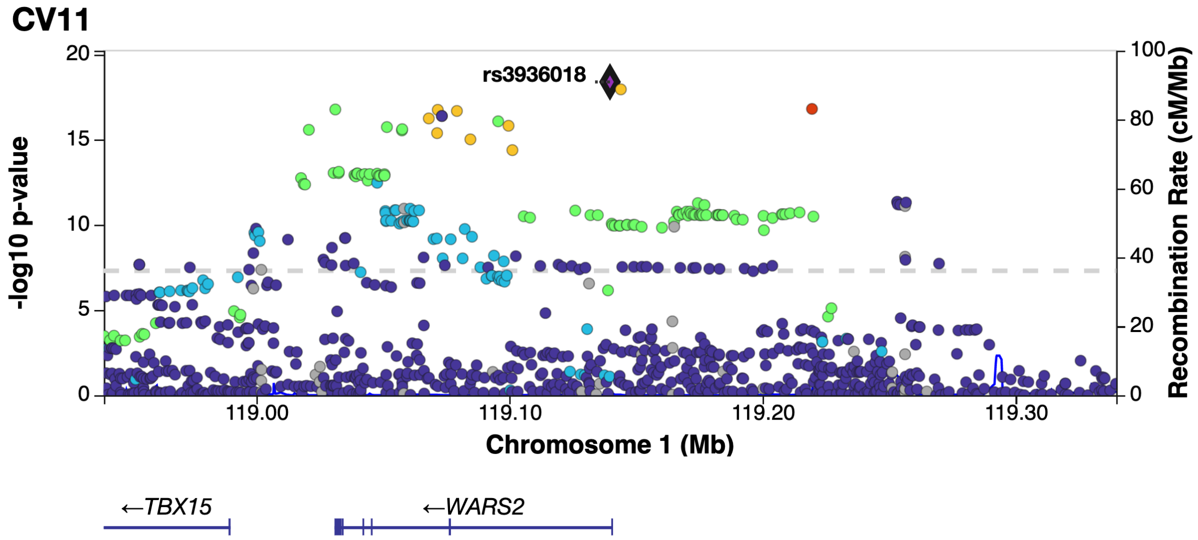

B

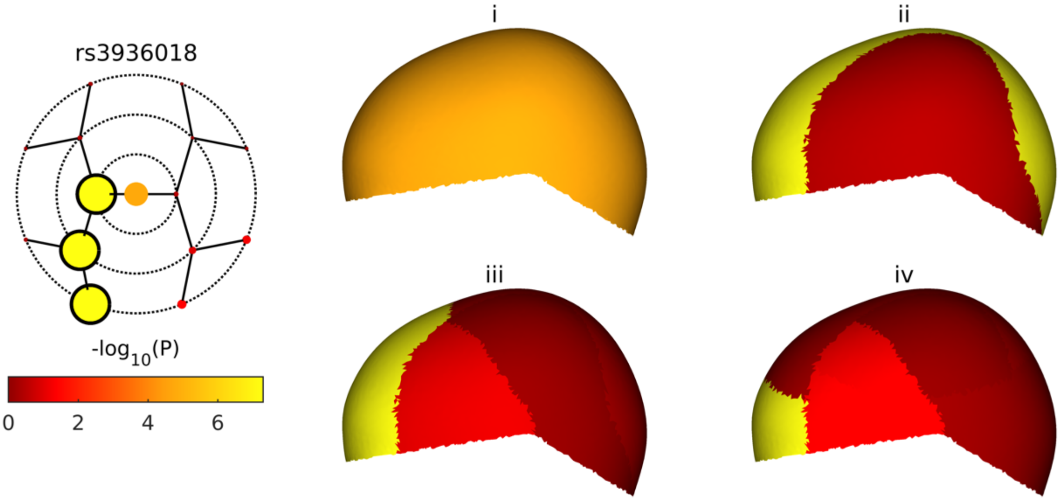

C

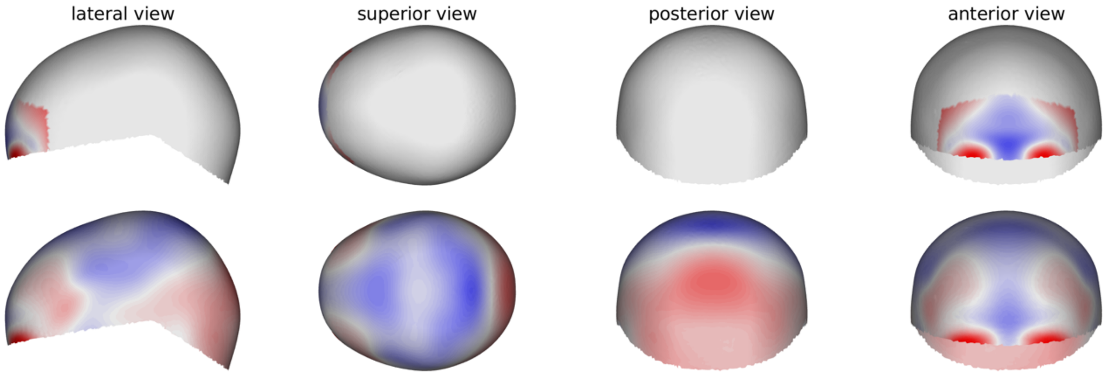

See page 2 for captions

rs2009778

A

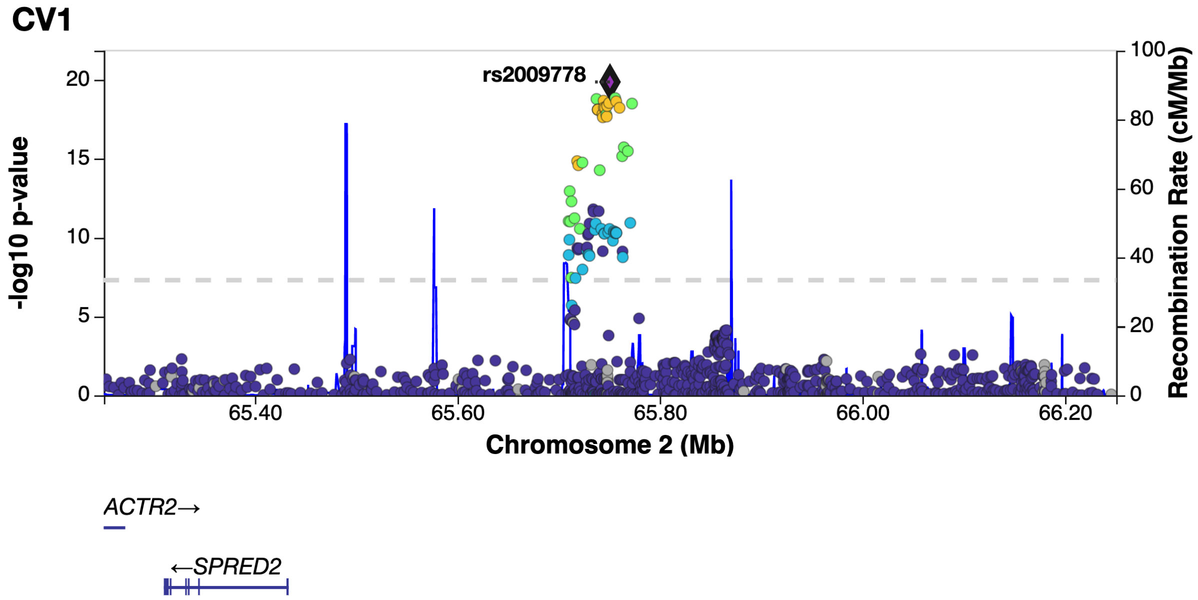

B

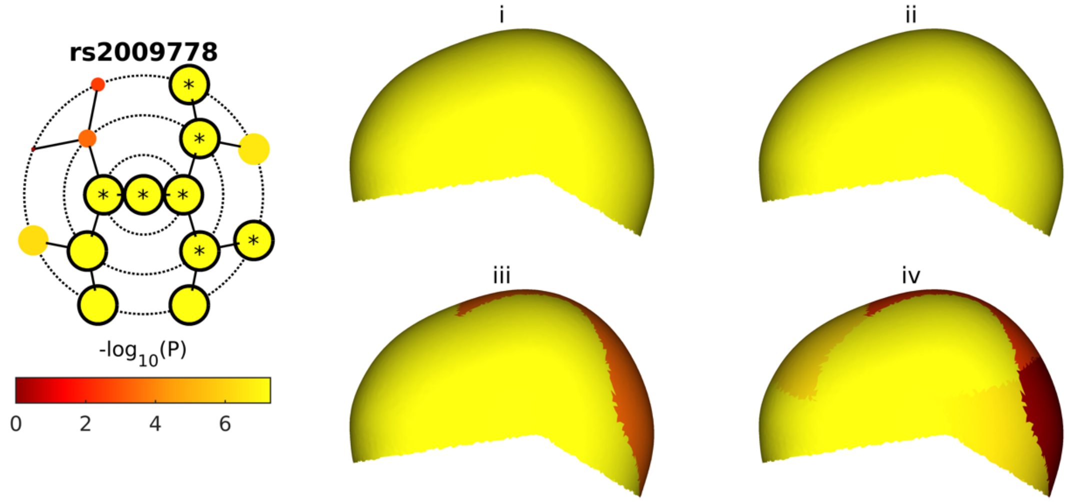

C

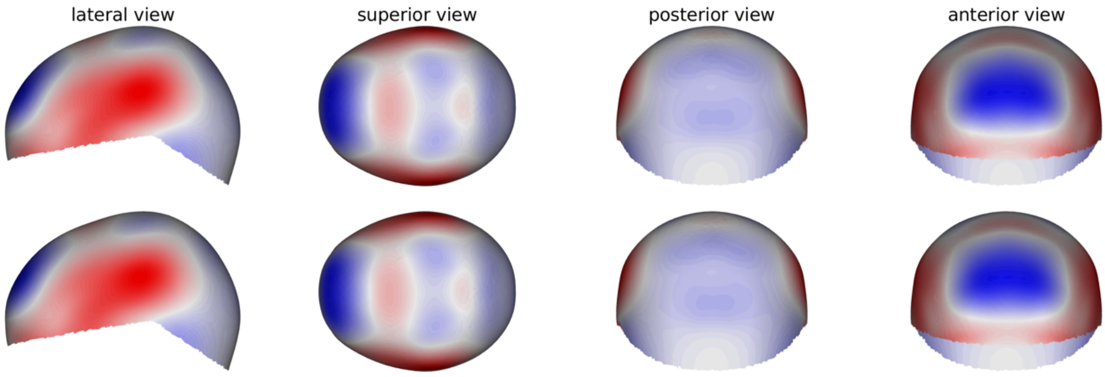

See page 2 for captions

rs6739488

A

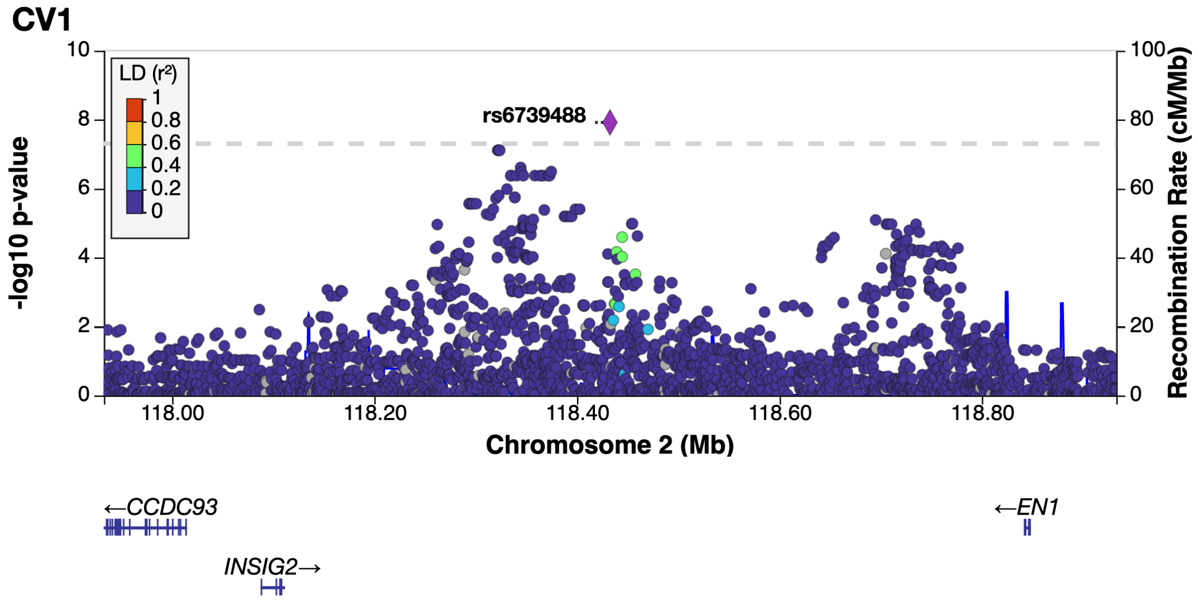

B

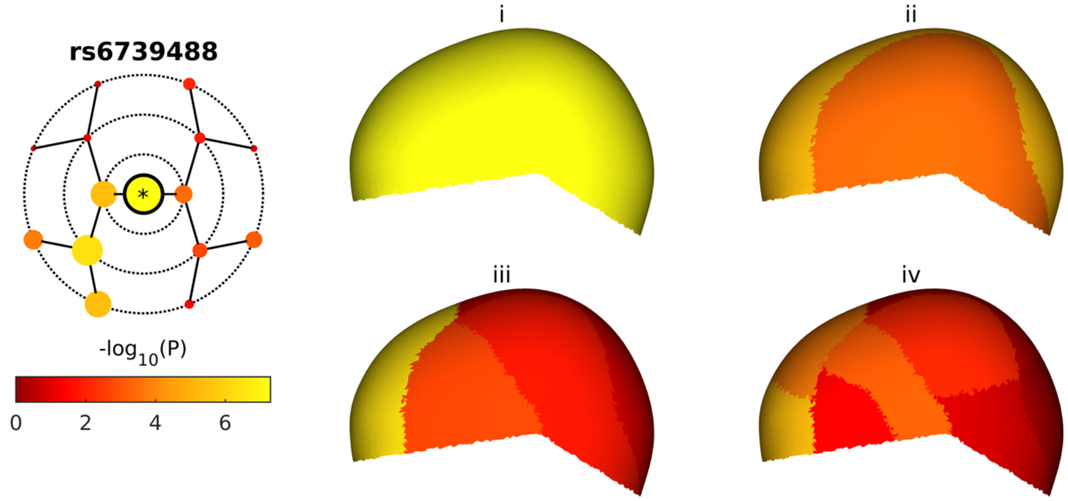

C

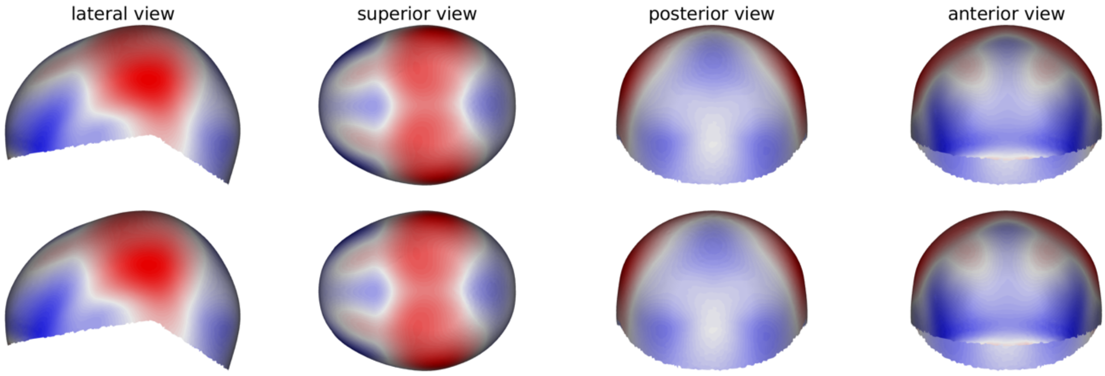

See page 2 for captions

rs17479393

A

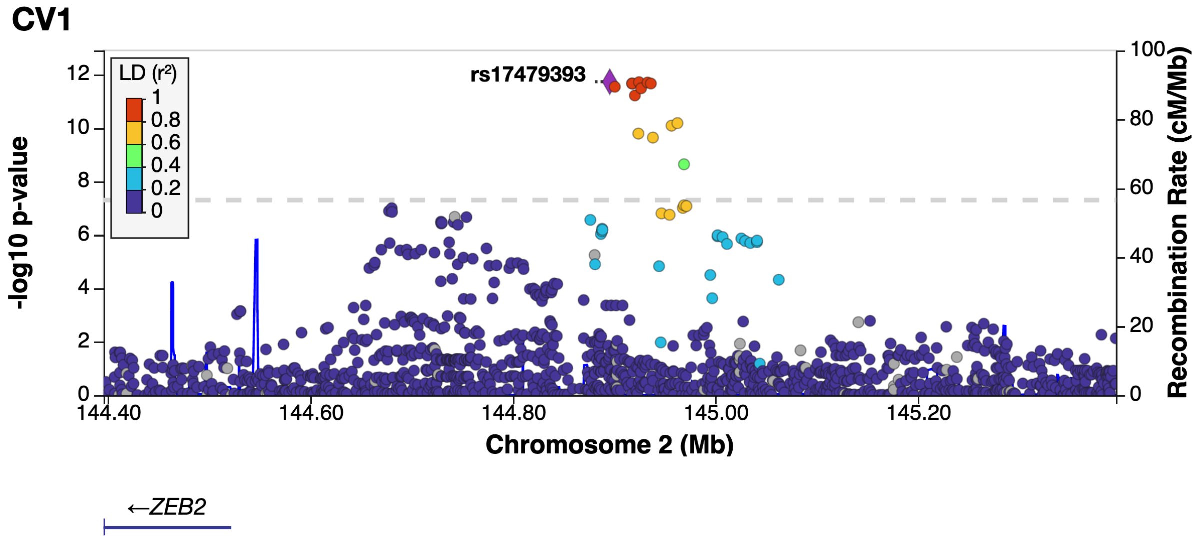

B

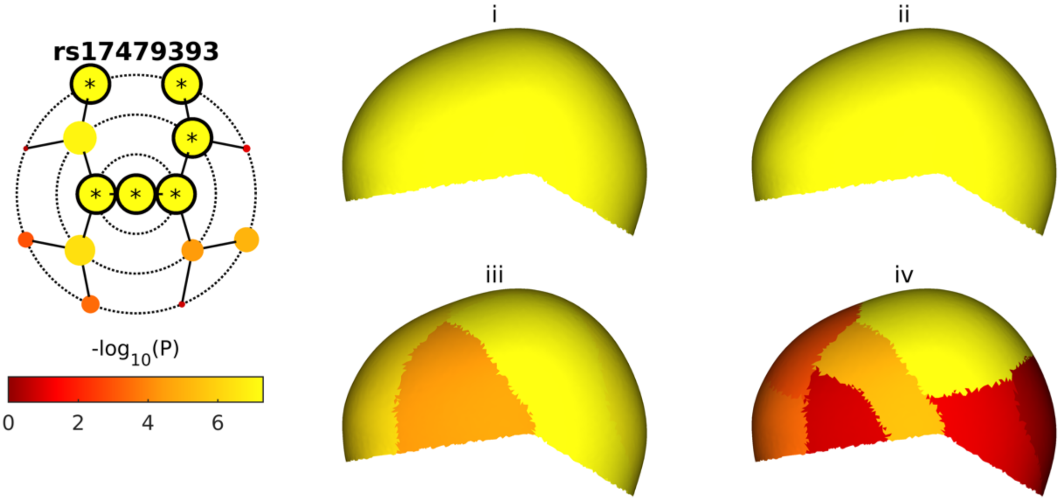

C

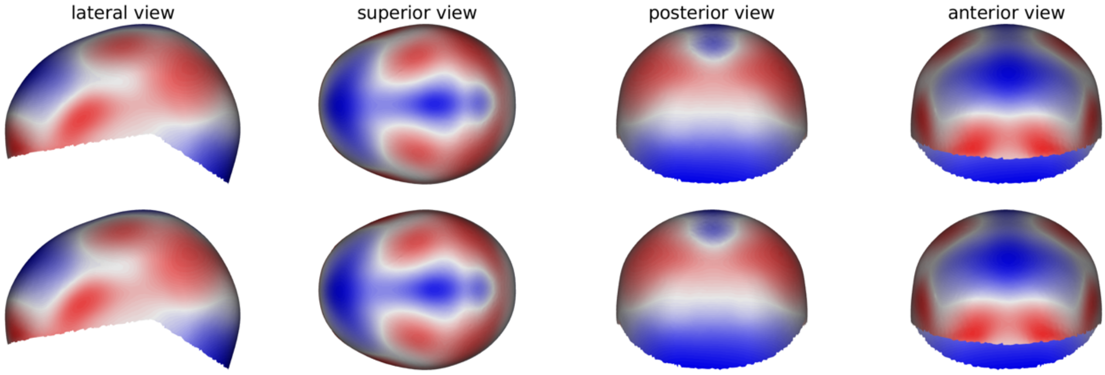

See page 2 for captions

rs970797

A

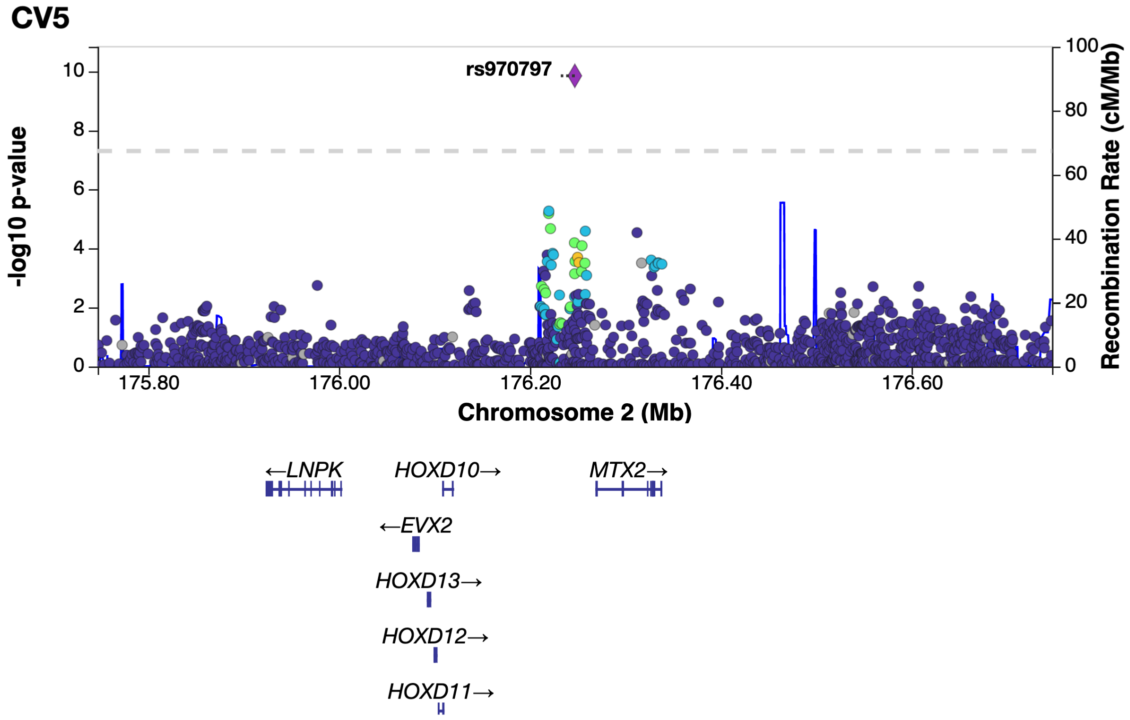

B

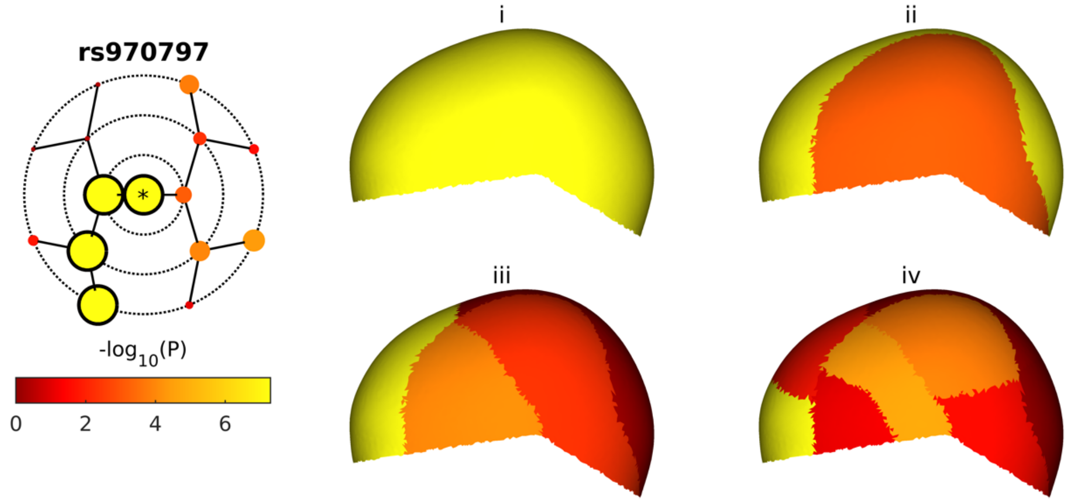

C

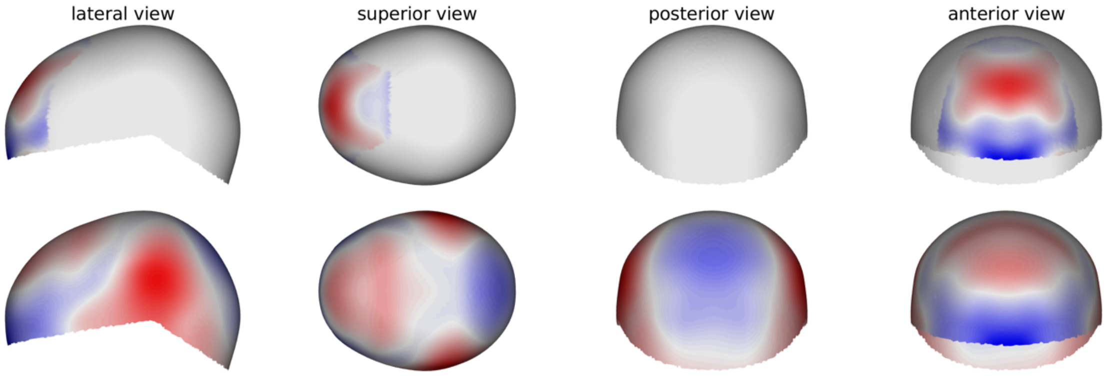

See page 2 for captions

rs7626244

A

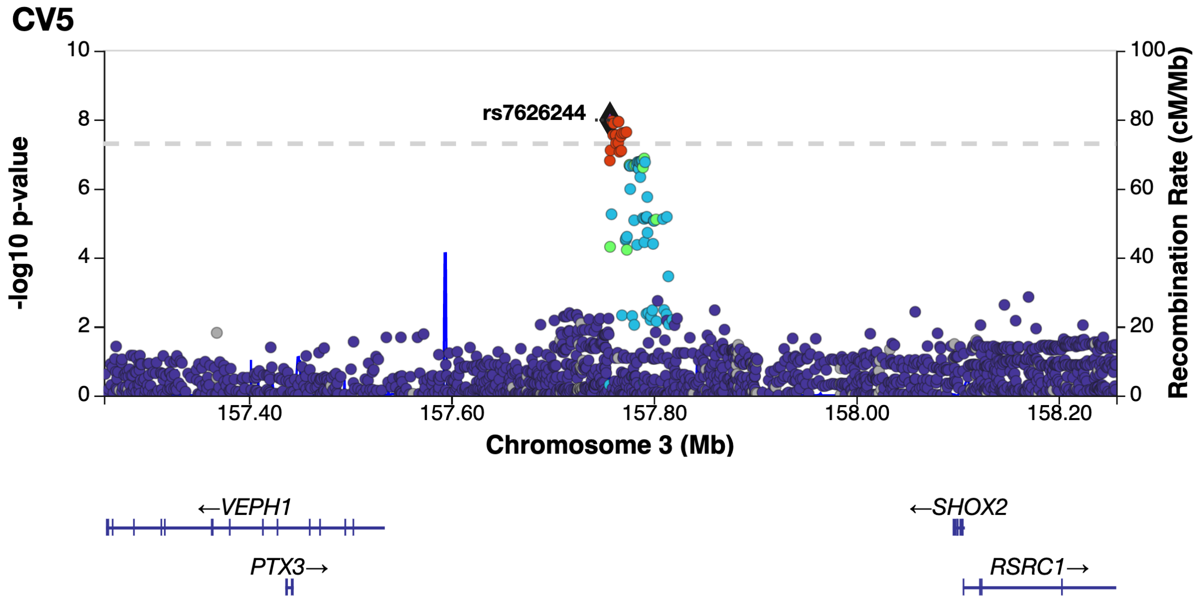

B

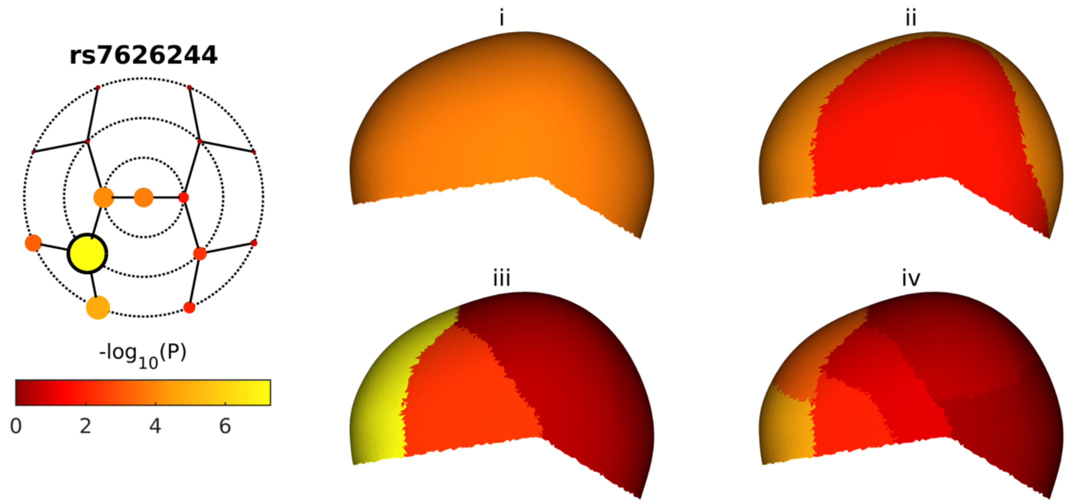

C

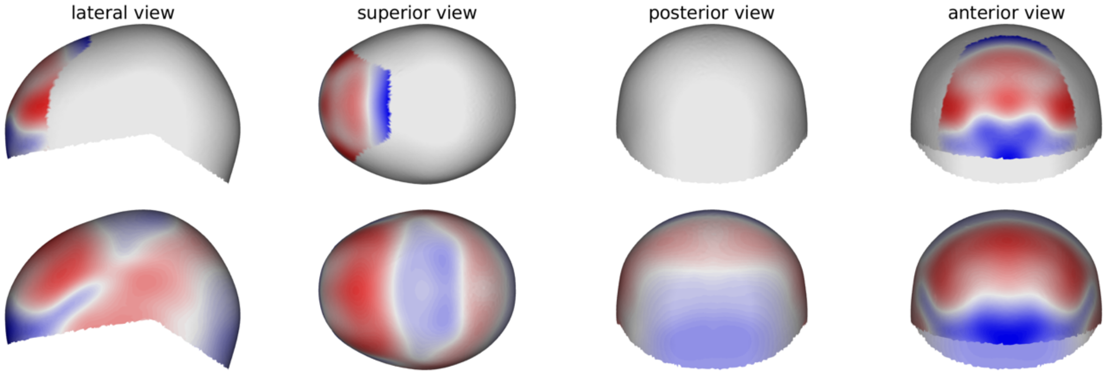

See page 2 for captions

rs35614773

A

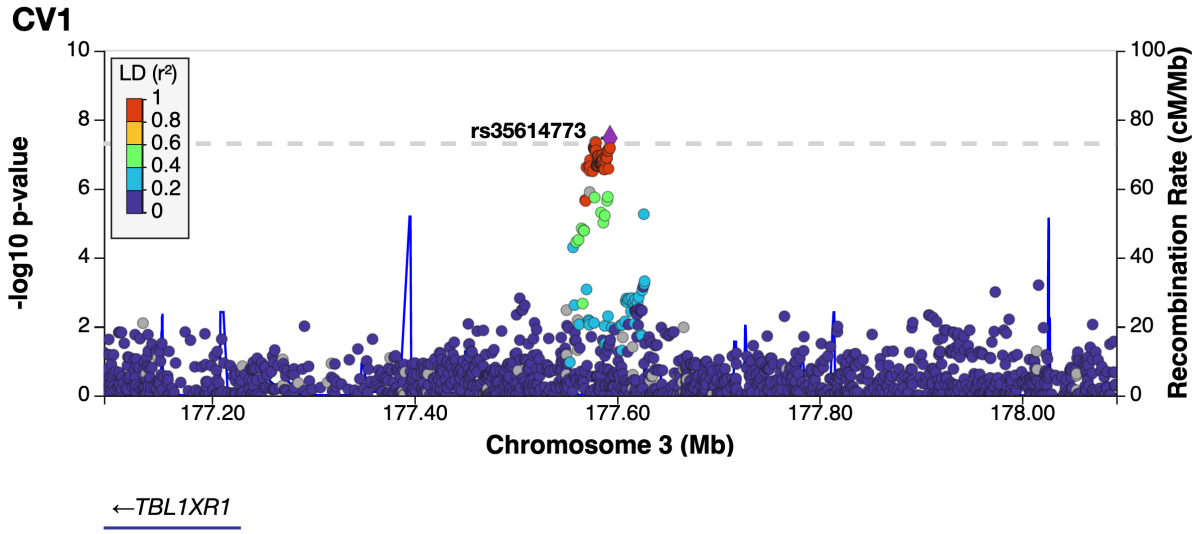

B

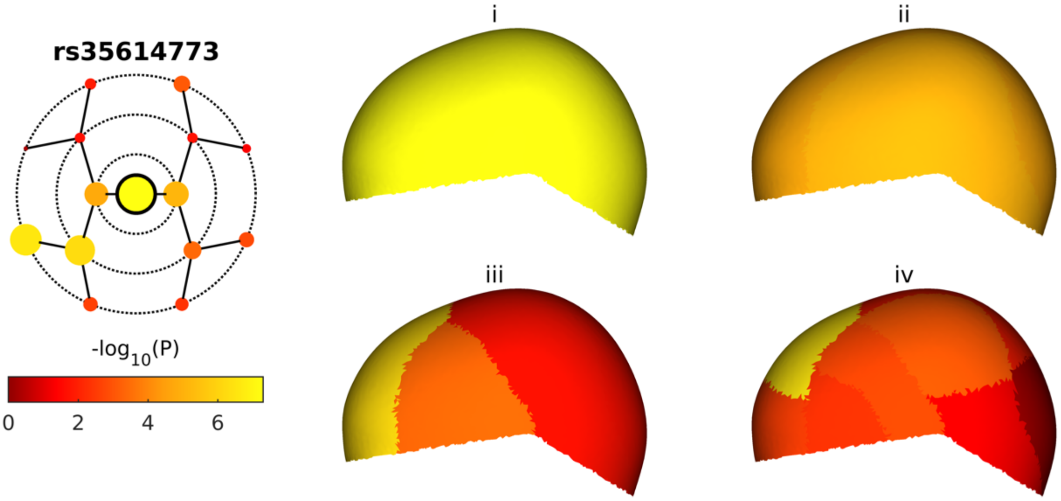

C

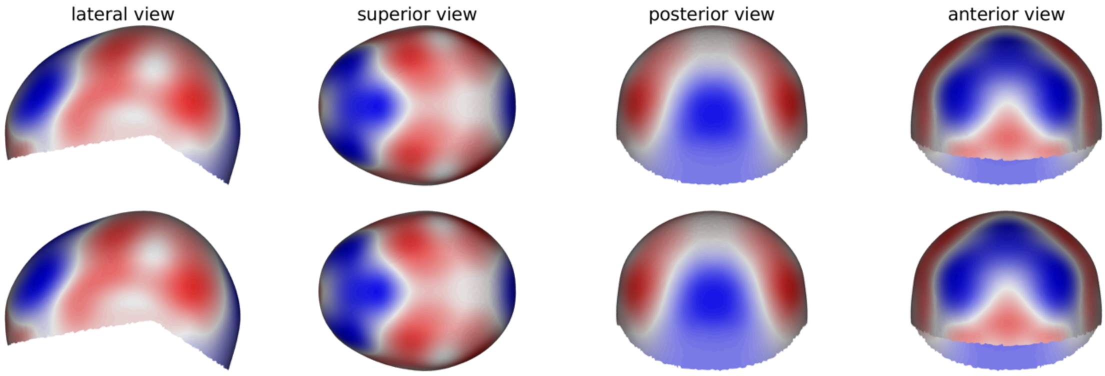

See page 2 for captions

rs1351637

A

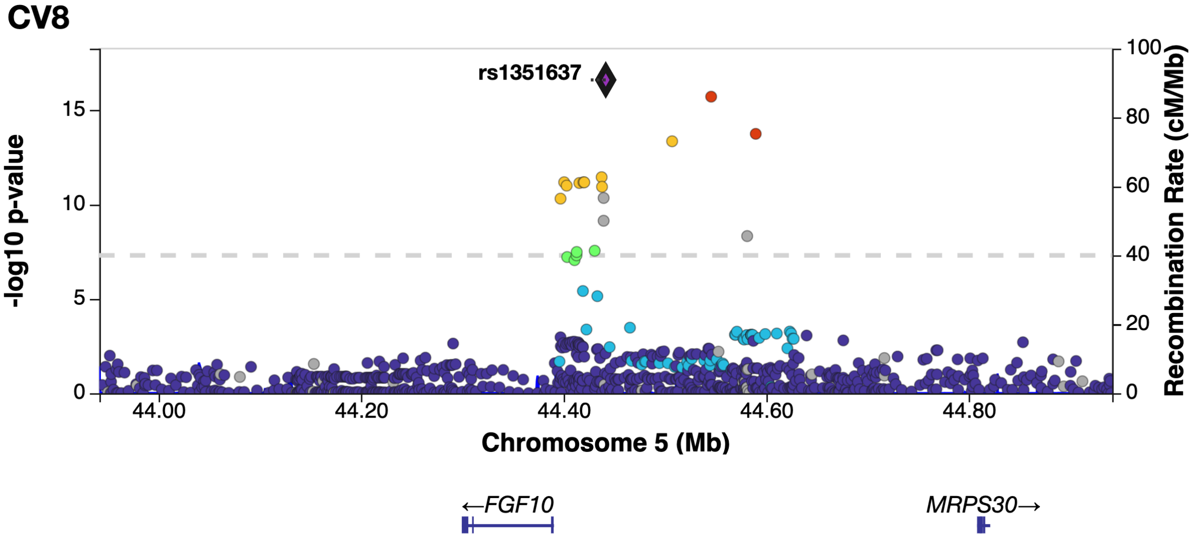

B

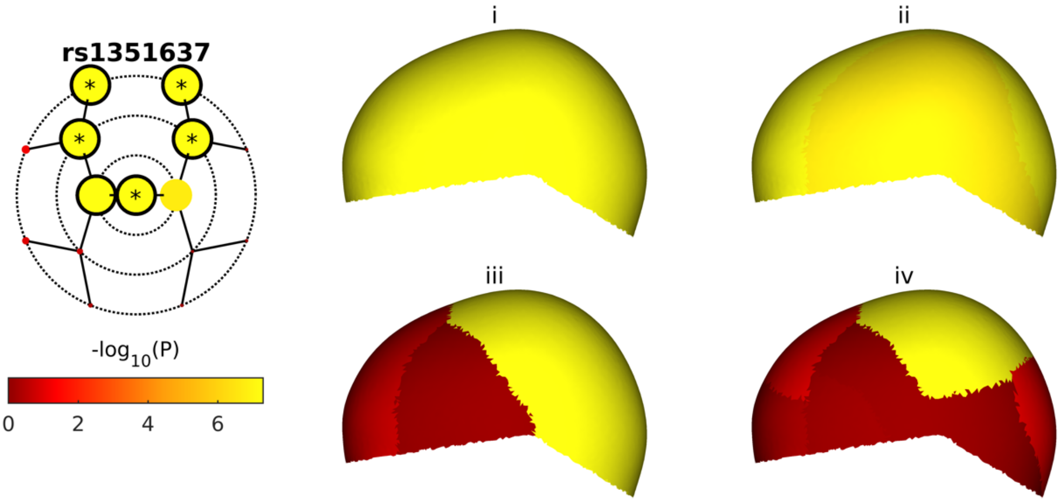

C

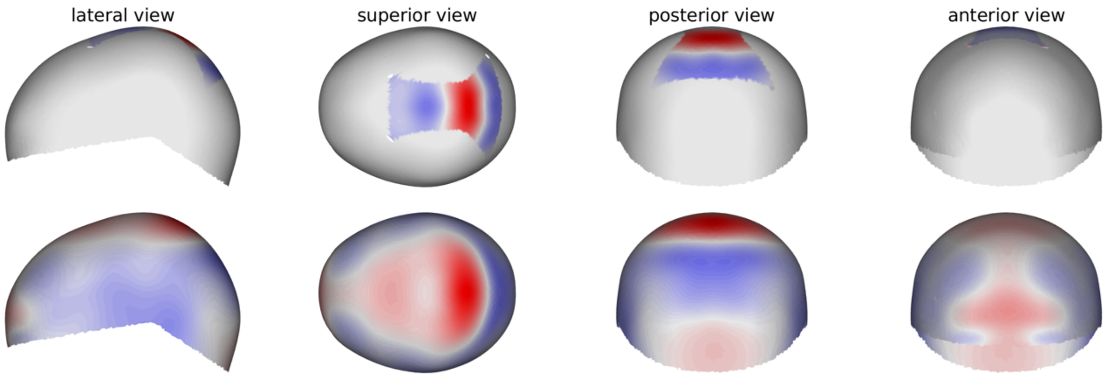

See page 2 for captions

rs3822730

A

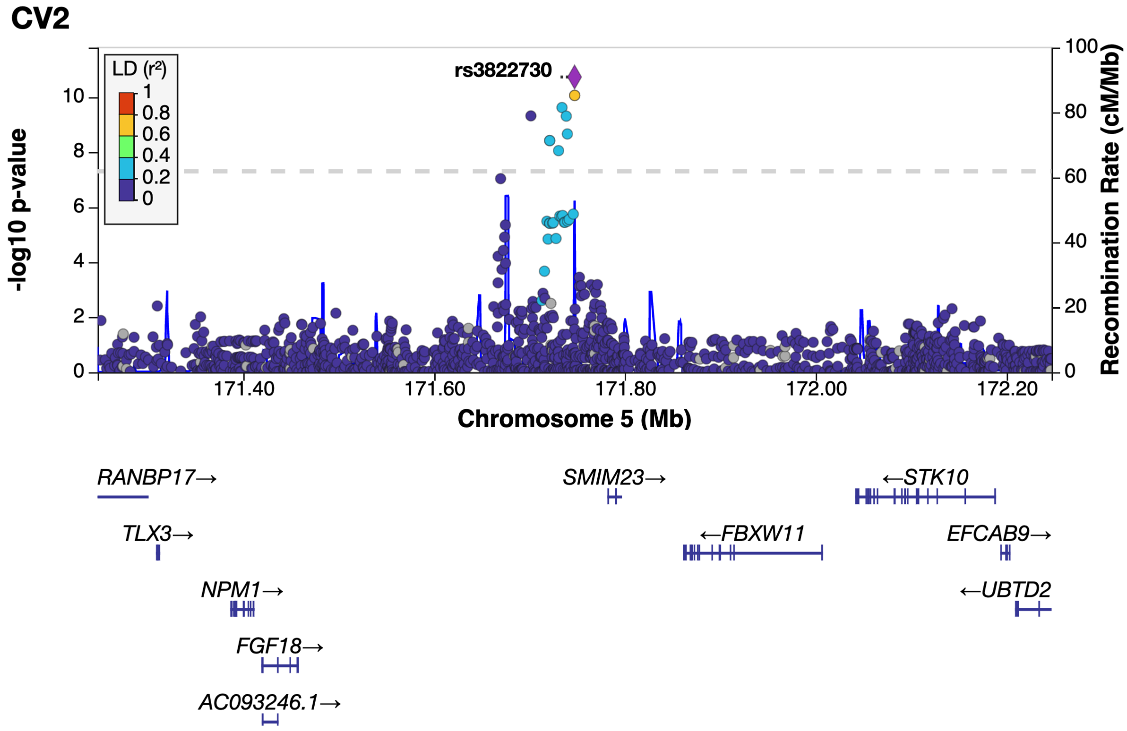

B

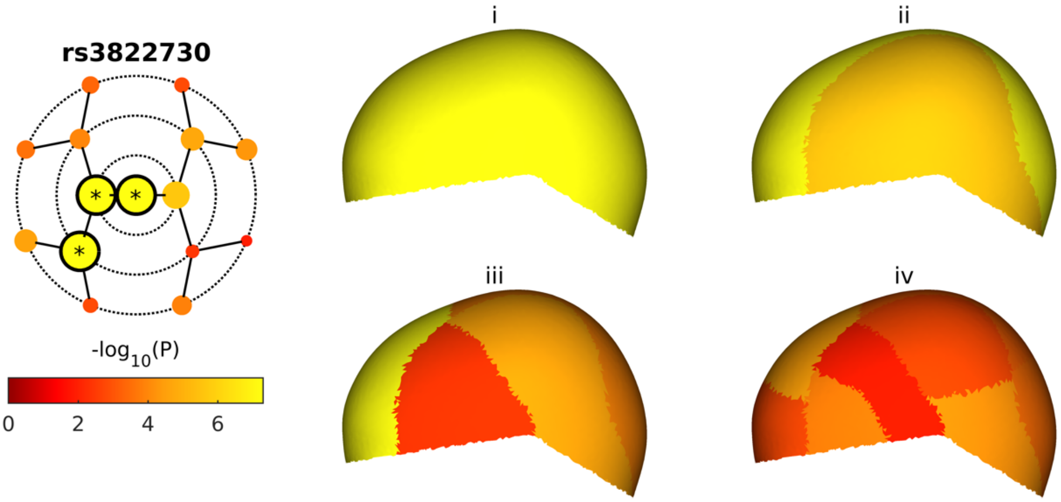

C

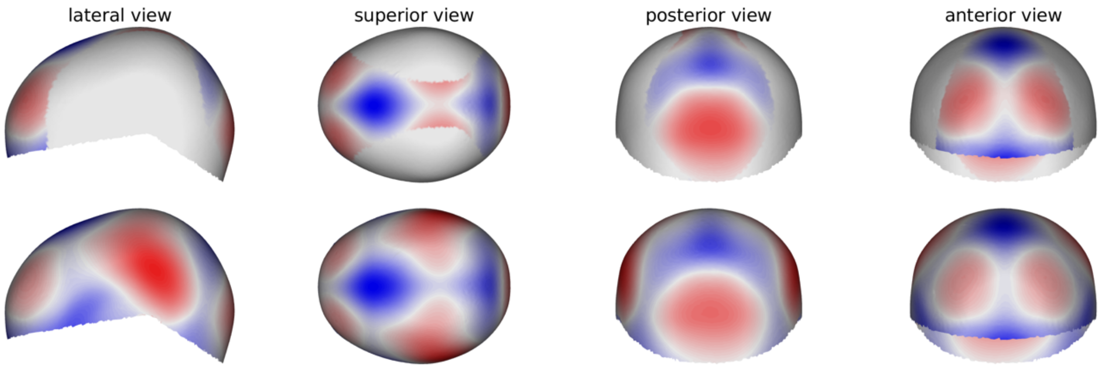

See page 2 for captions

rs4714260

A

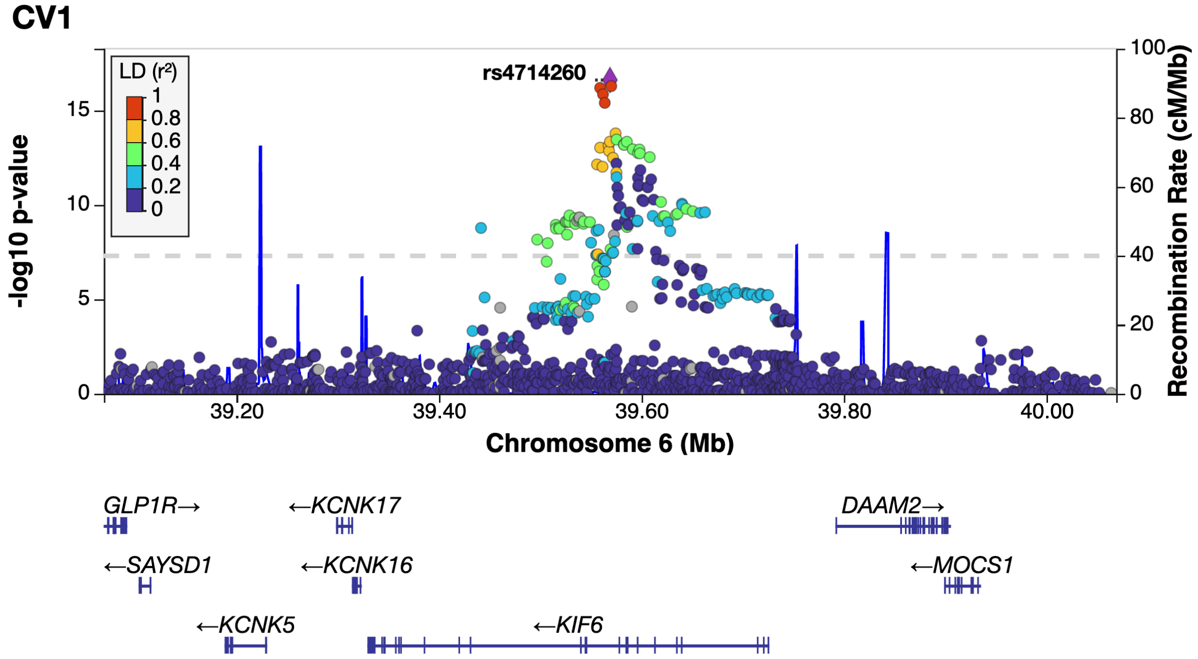

B

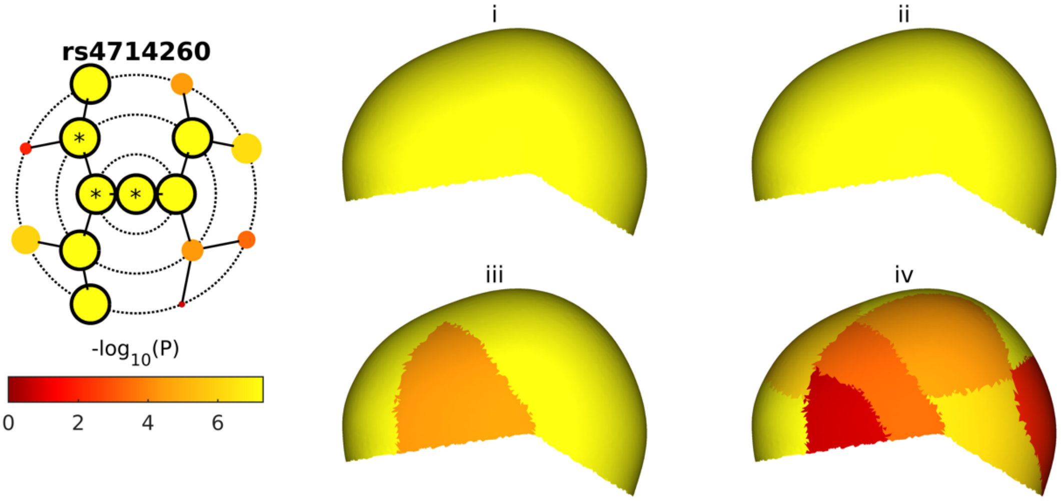

C

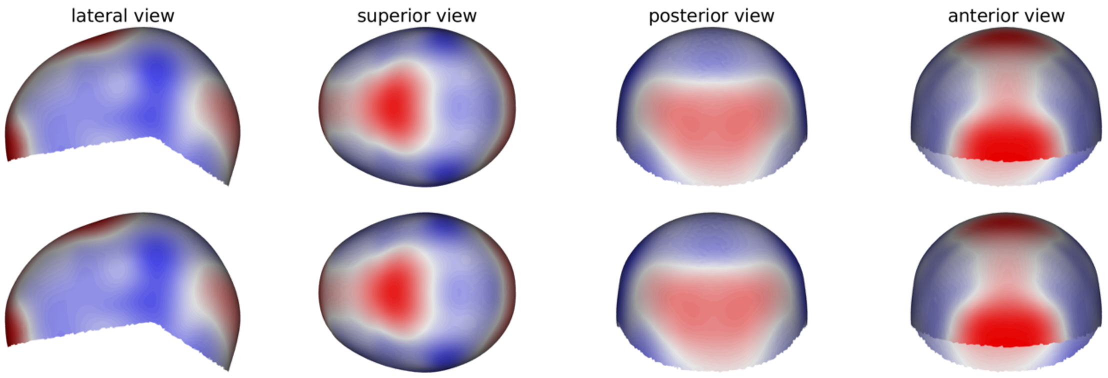

See page 2 for captions

rs3799970

A

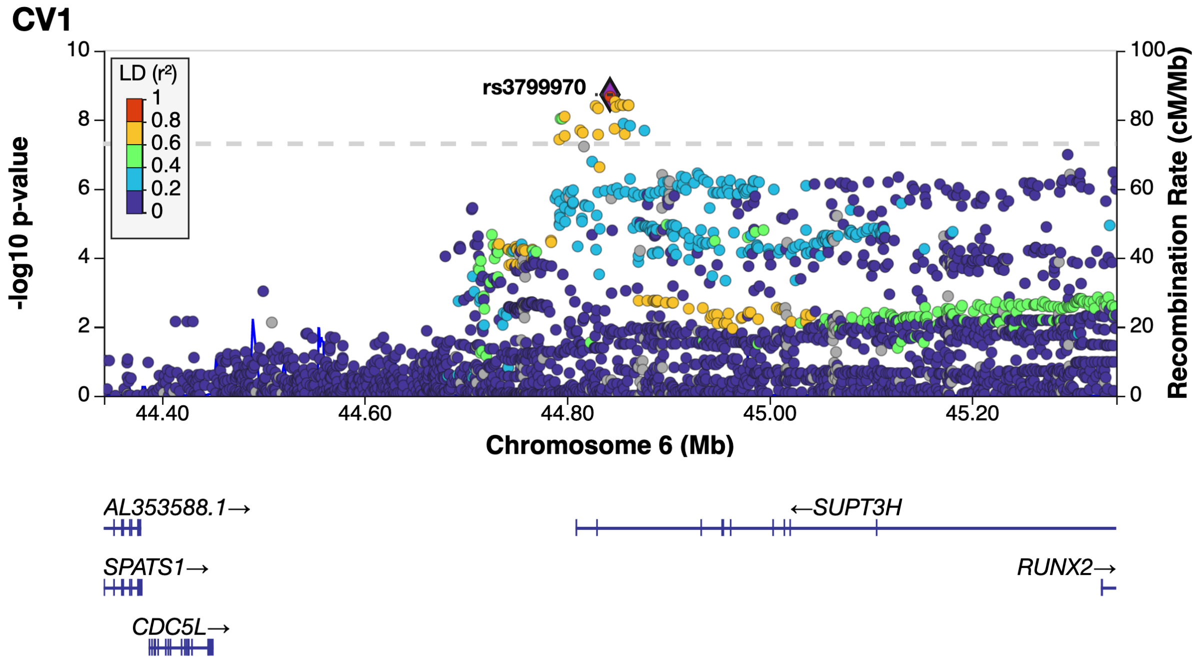

B

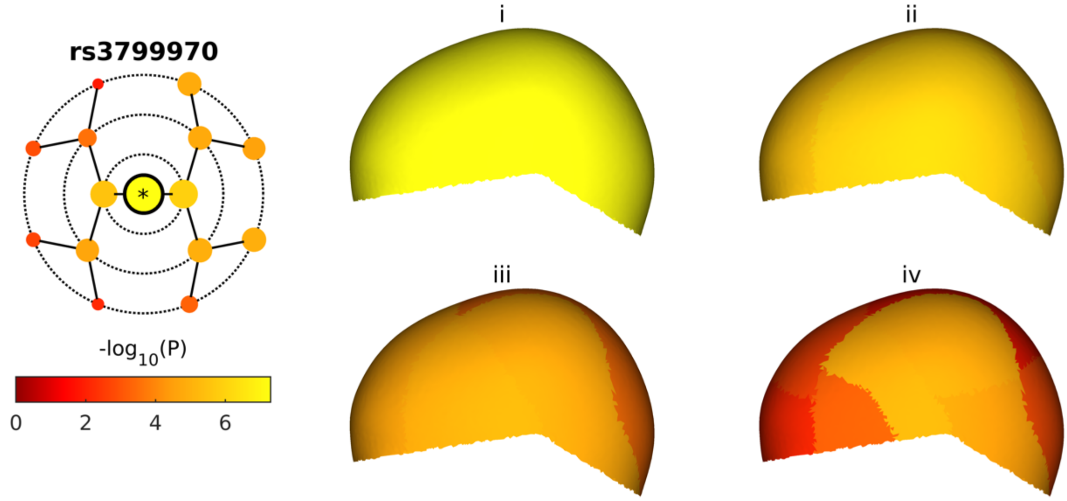

C

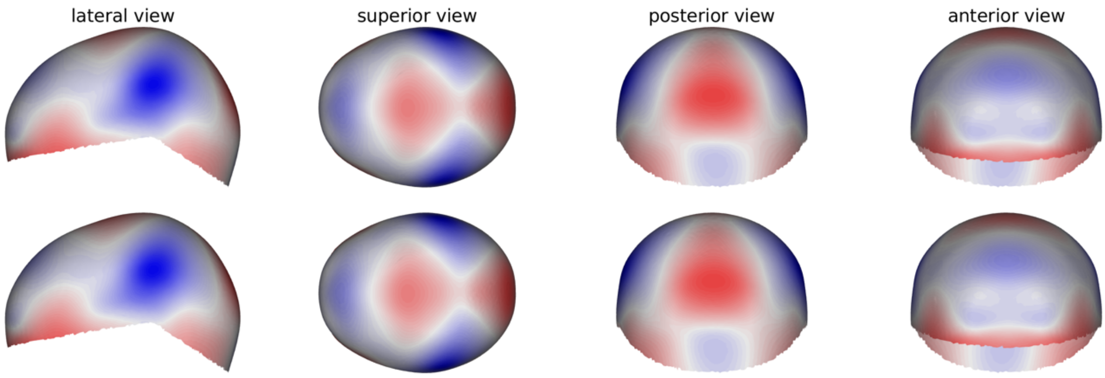

See page 2 for captions

rs9491697

A

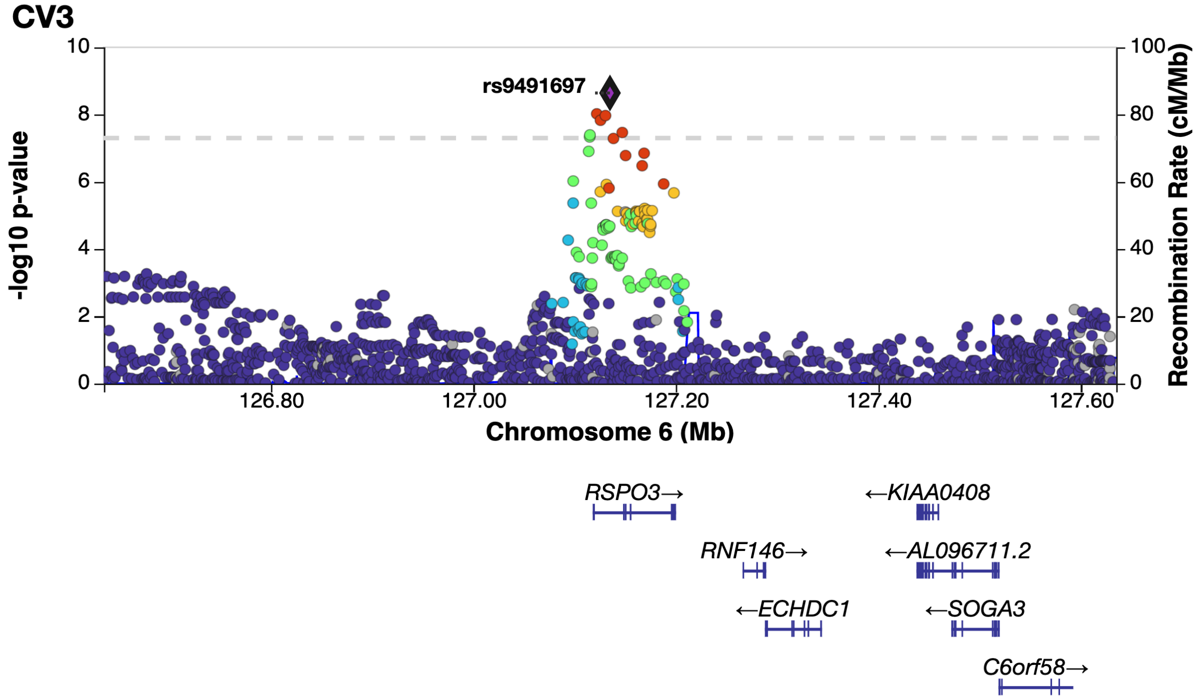

B

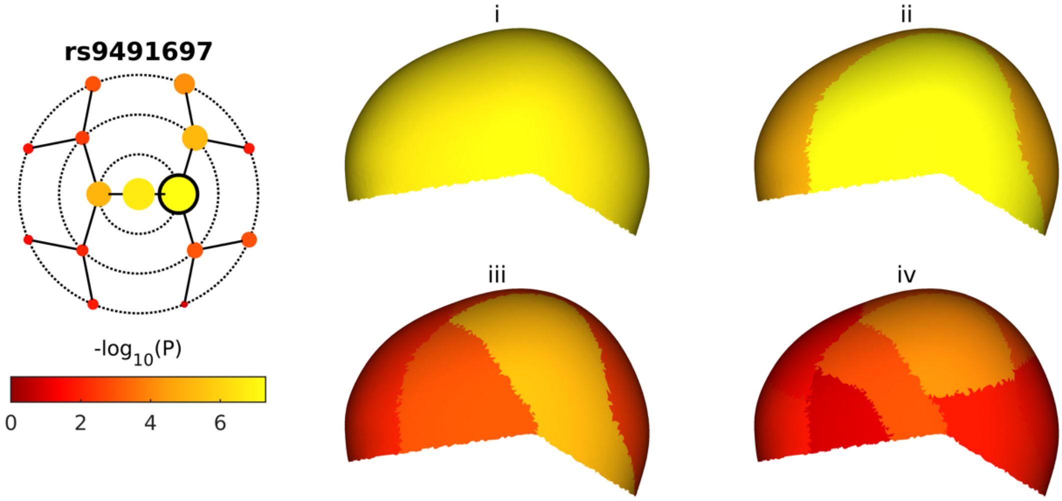

C

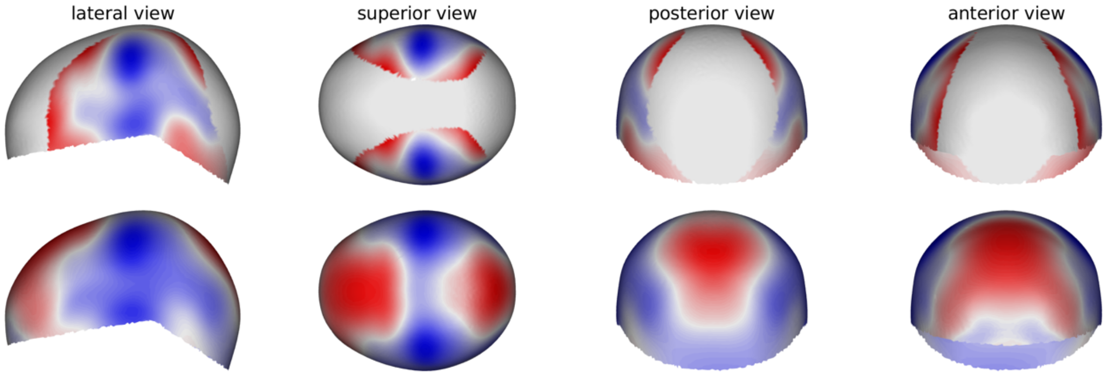

See page 2 for captions

rs296418

A

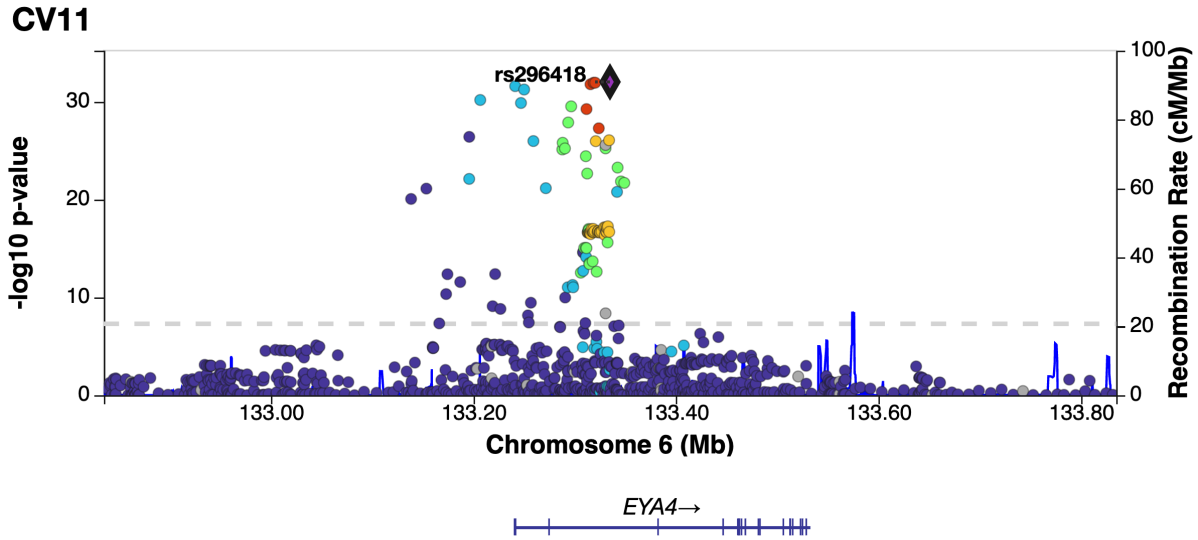

B

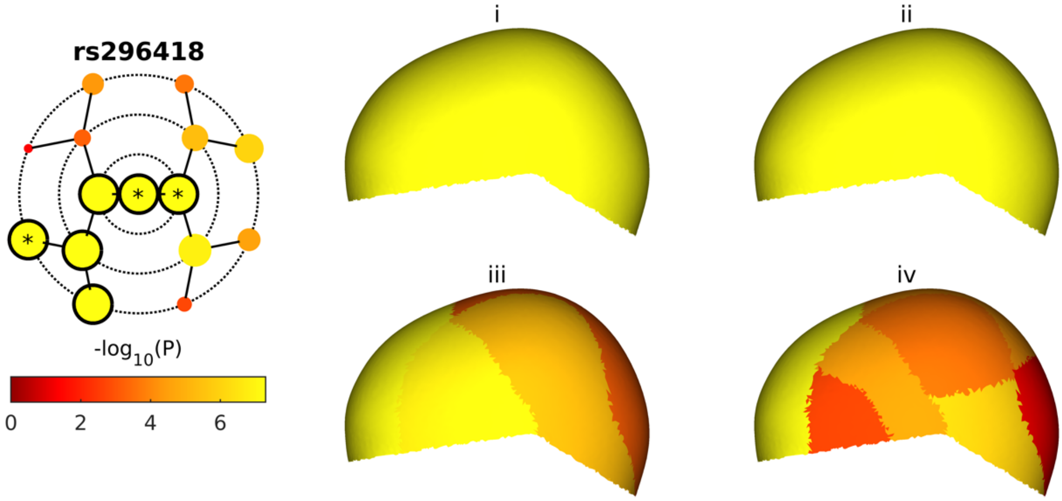

C

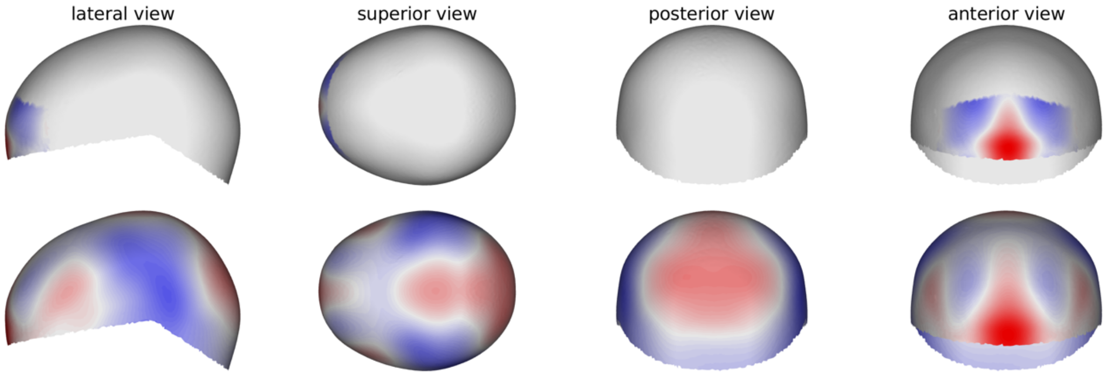

See page 2 for captions

rs148673350

A

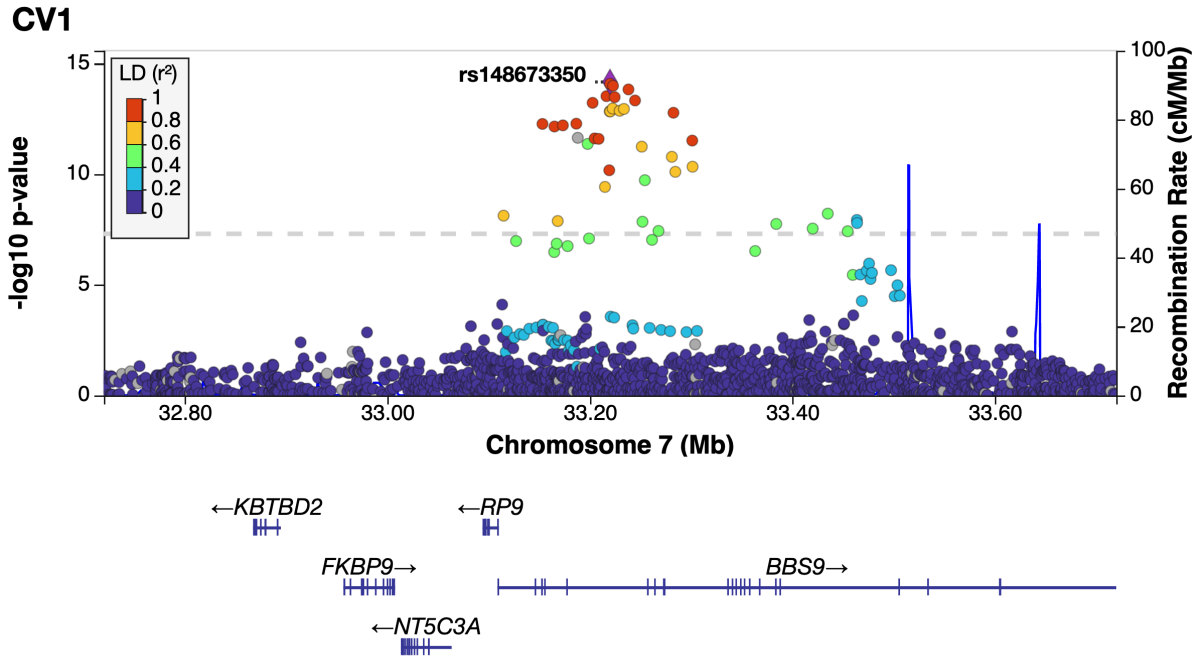

B

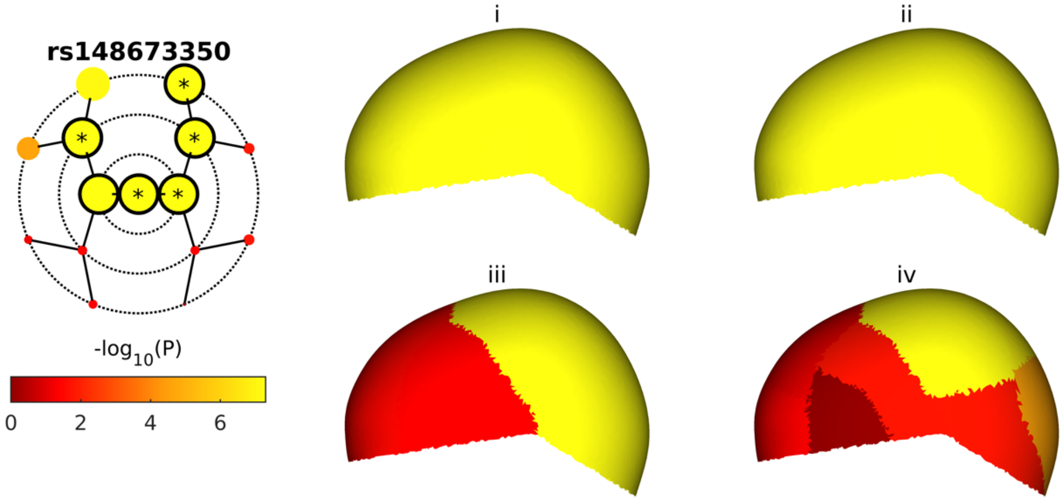

C

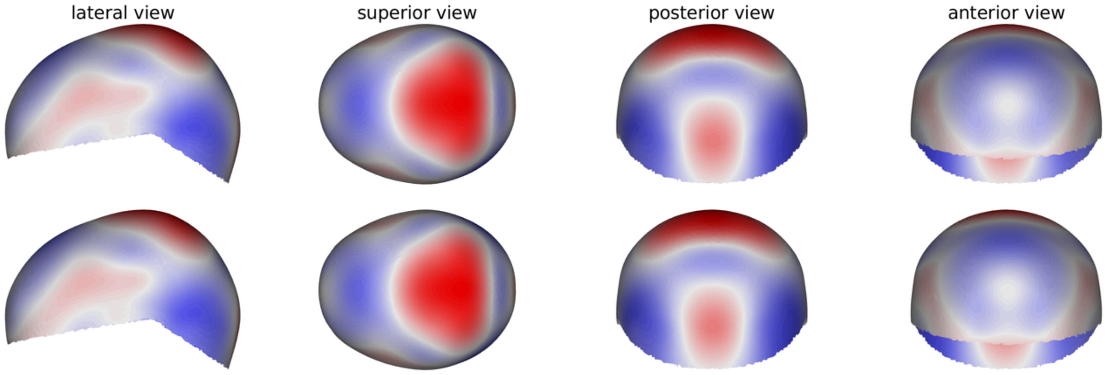

See page 2 for captions

rs202055590

A

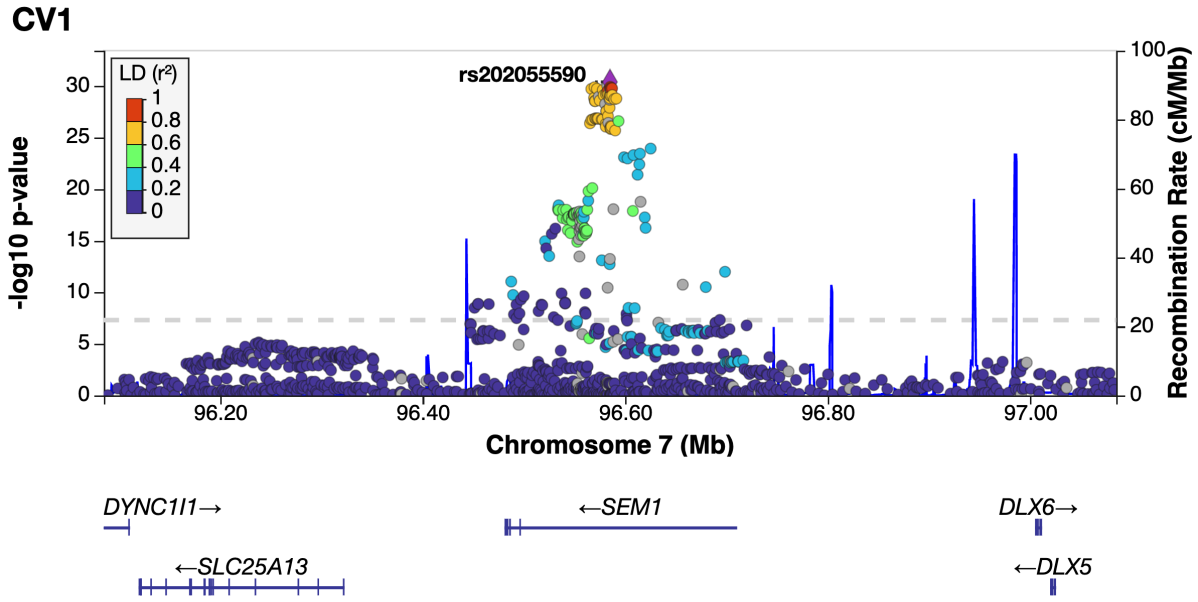

B

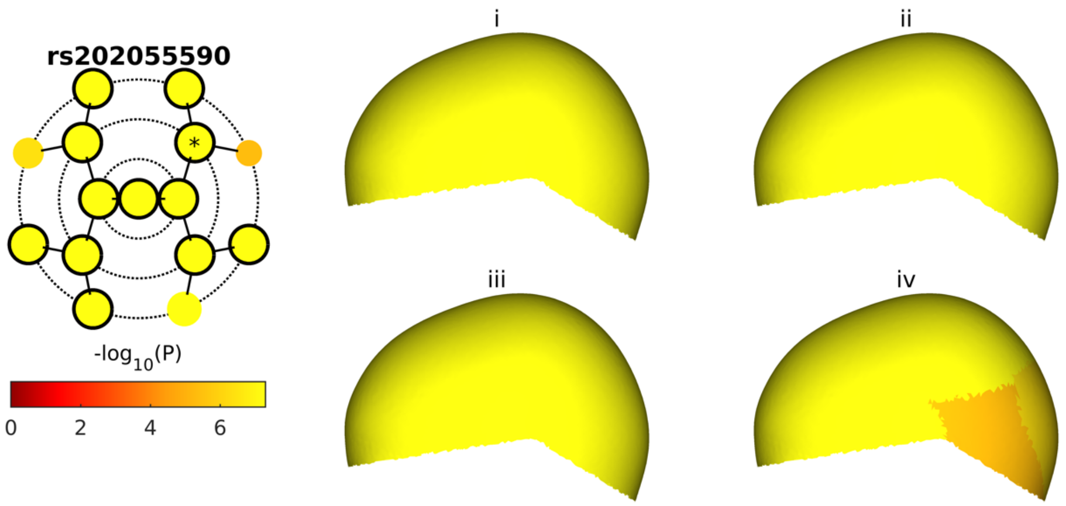

C

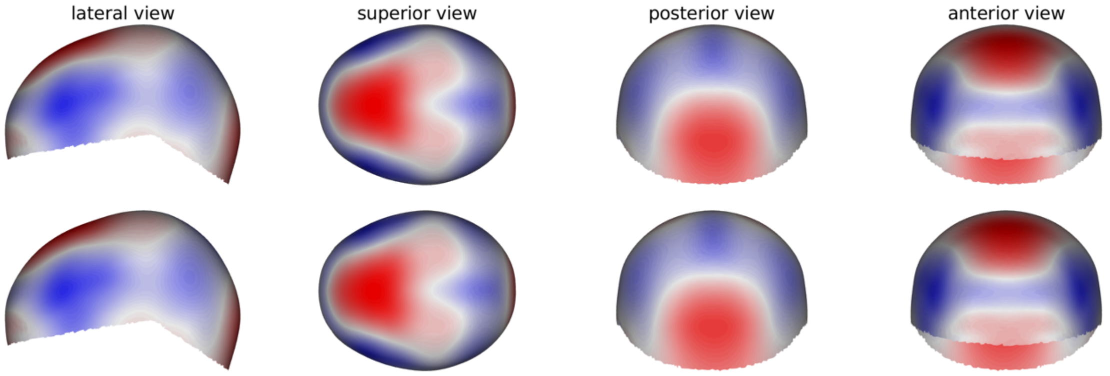

See page 2 for captions

rs1581525

A

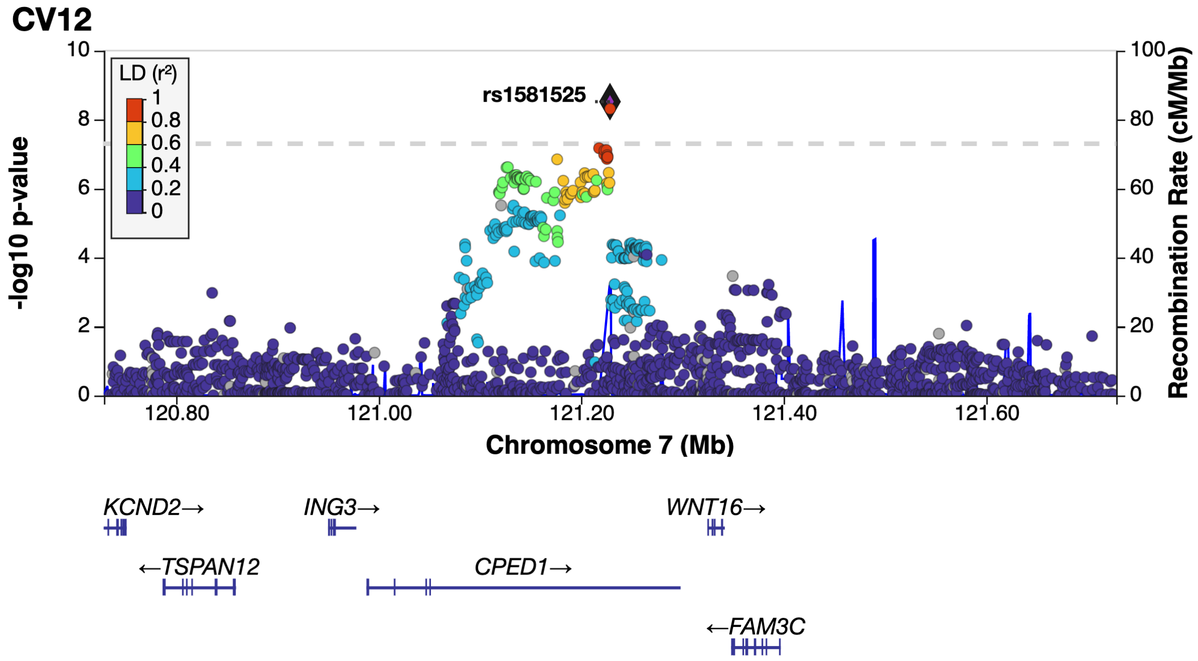

B

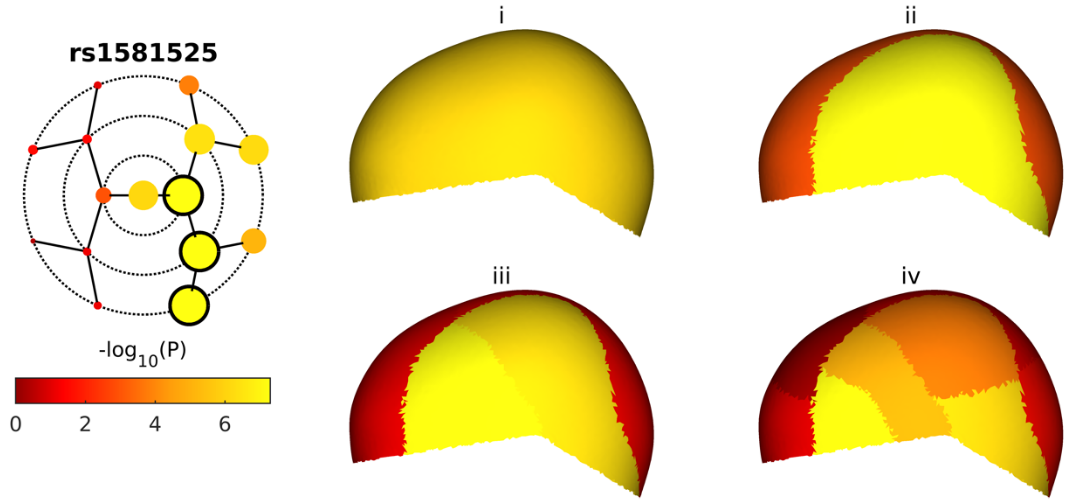

C

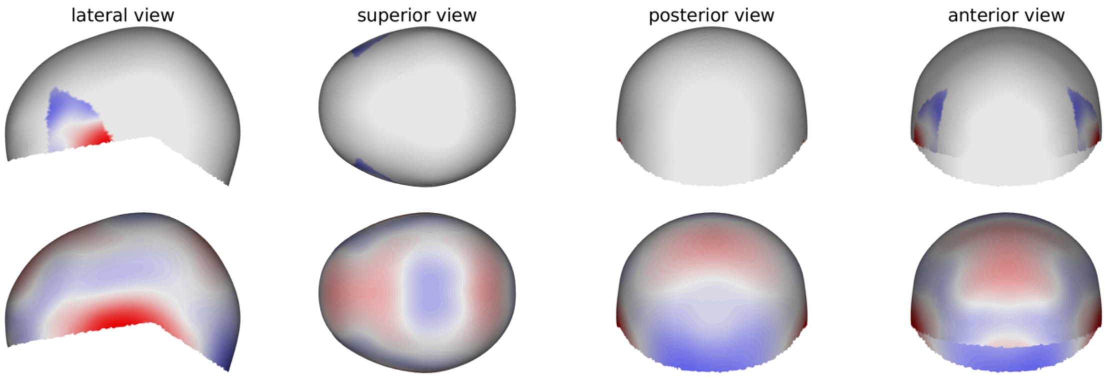

See page 2 for captions

rs147676525

A

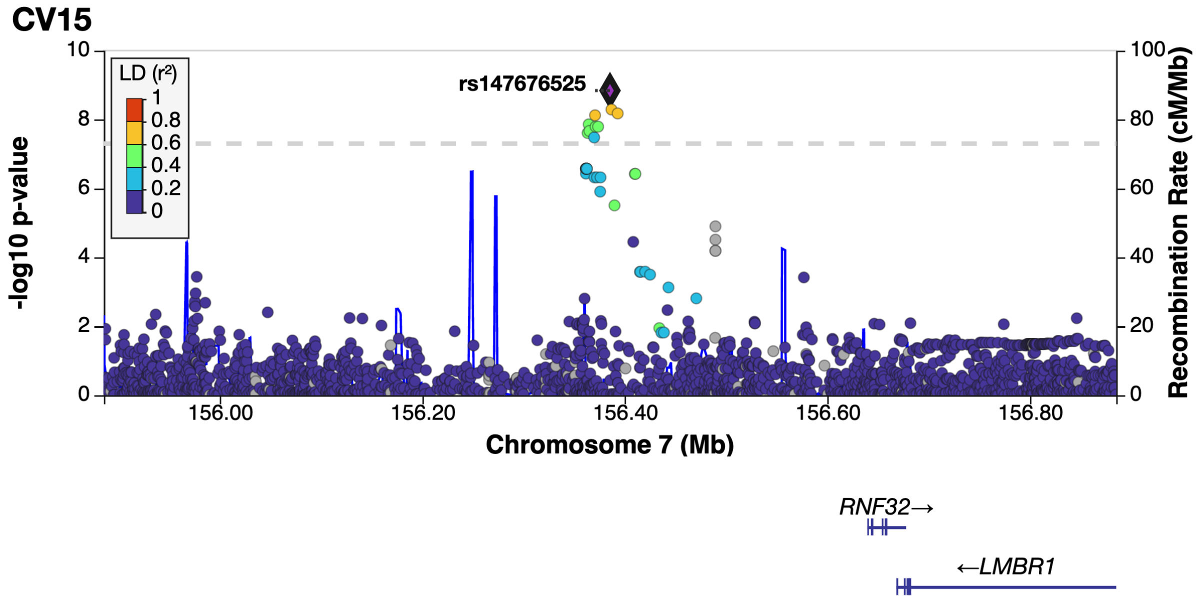

B

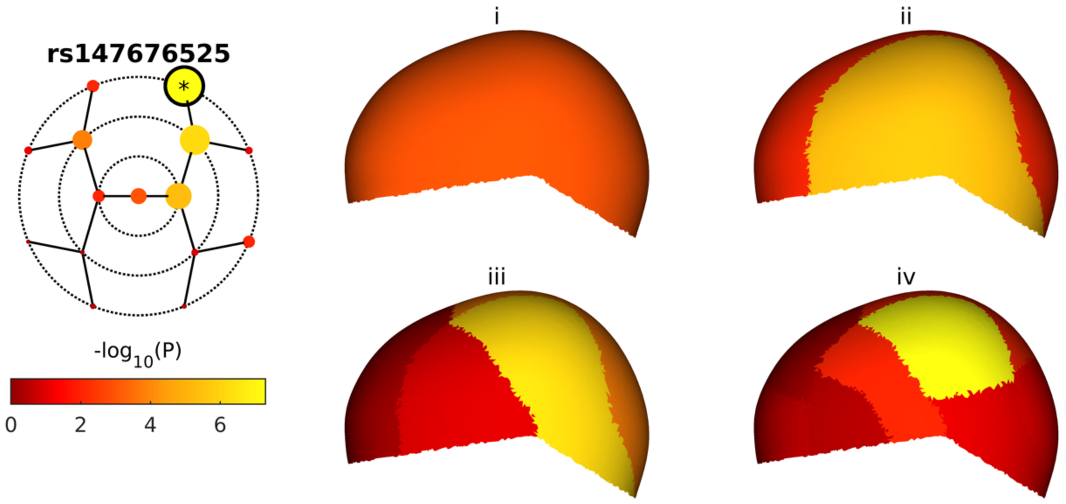

C

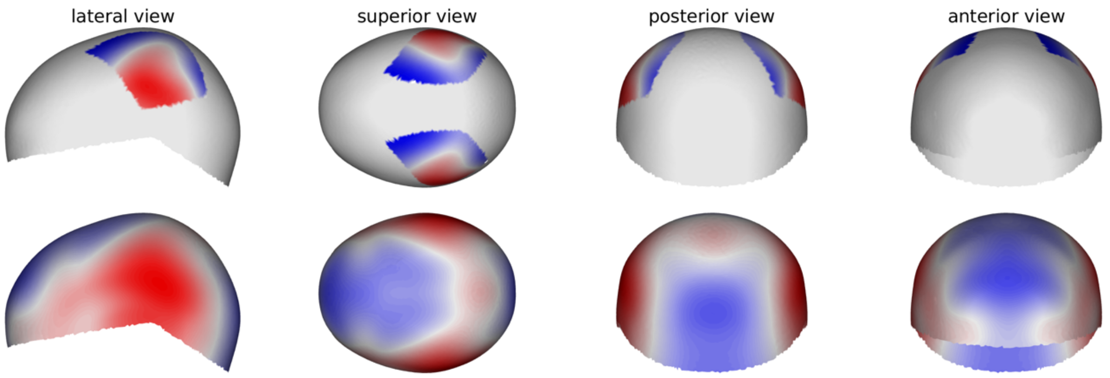

See page 2 for captions

rs7813717

A

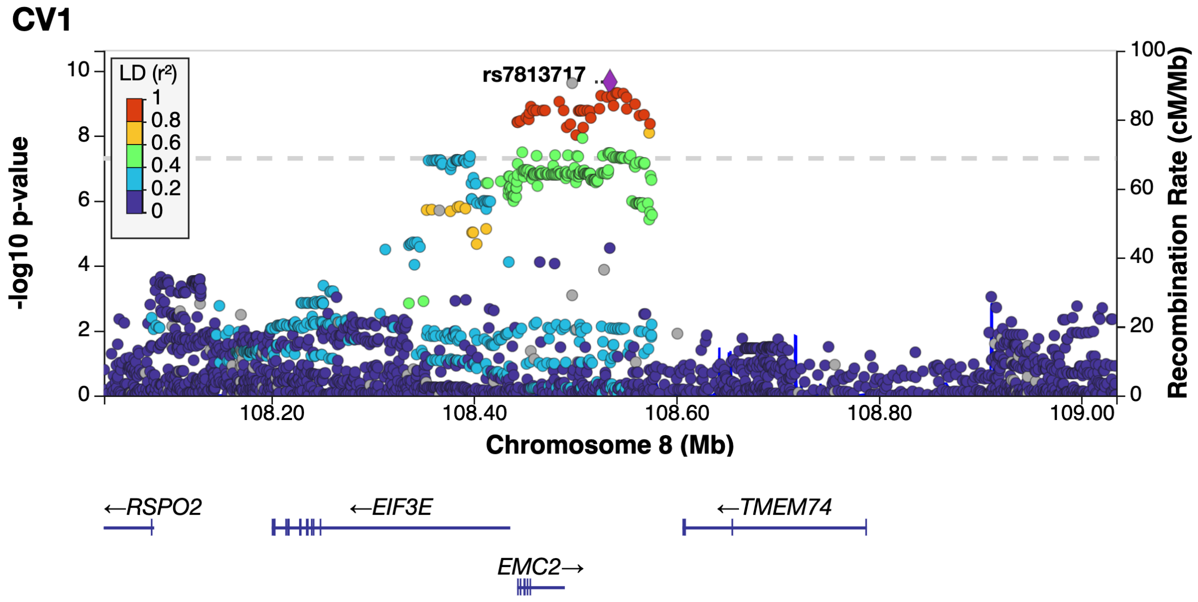

B

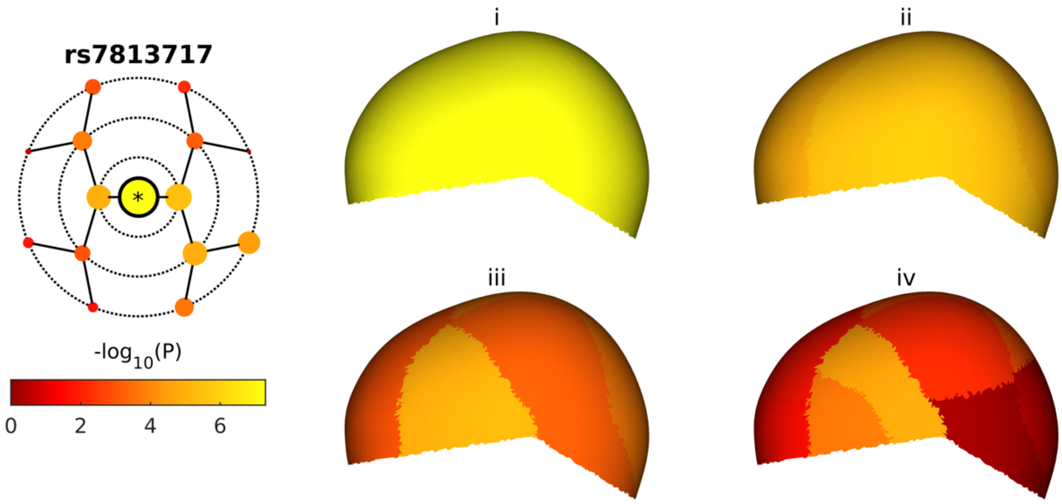

C

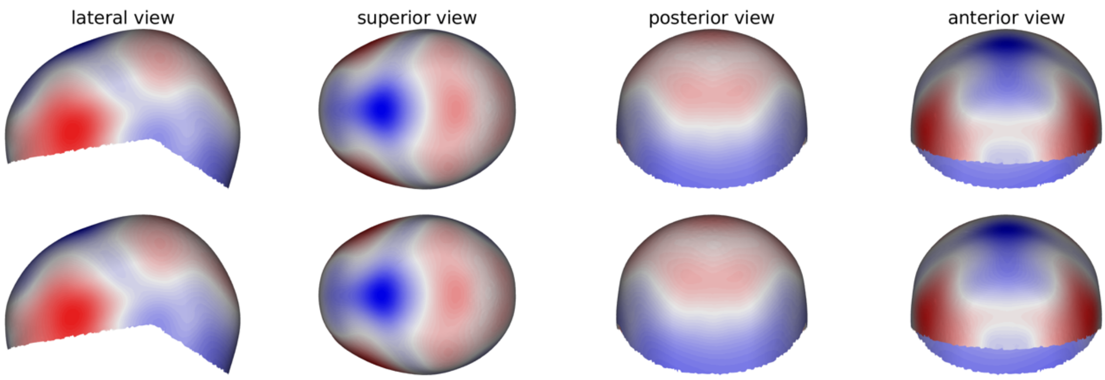

See page 2 for captions

rs10120728

A

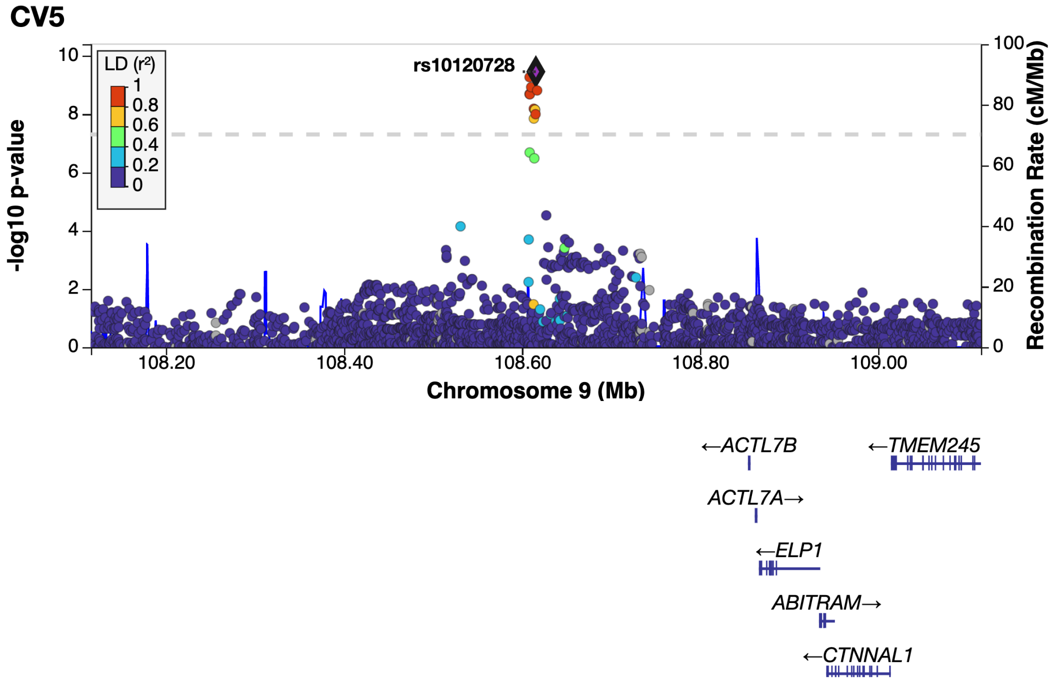

B

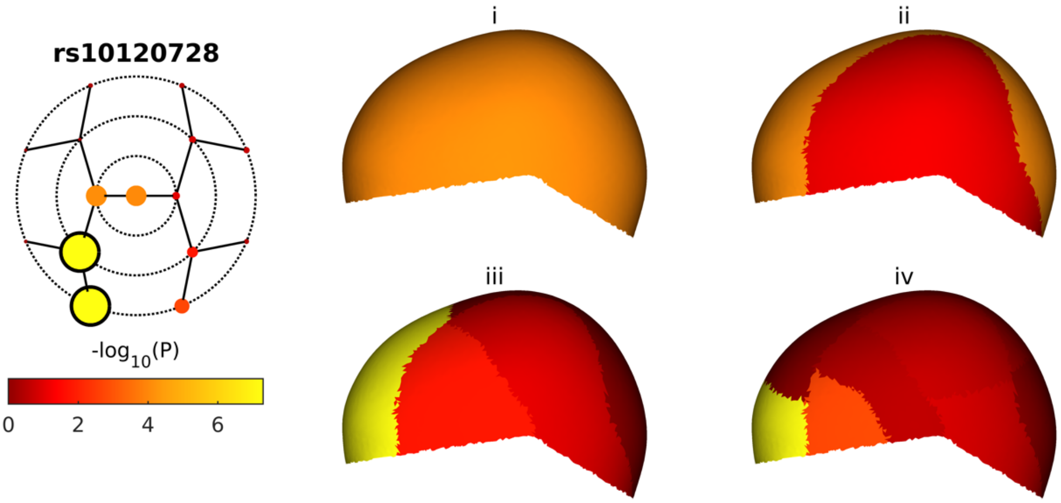

C

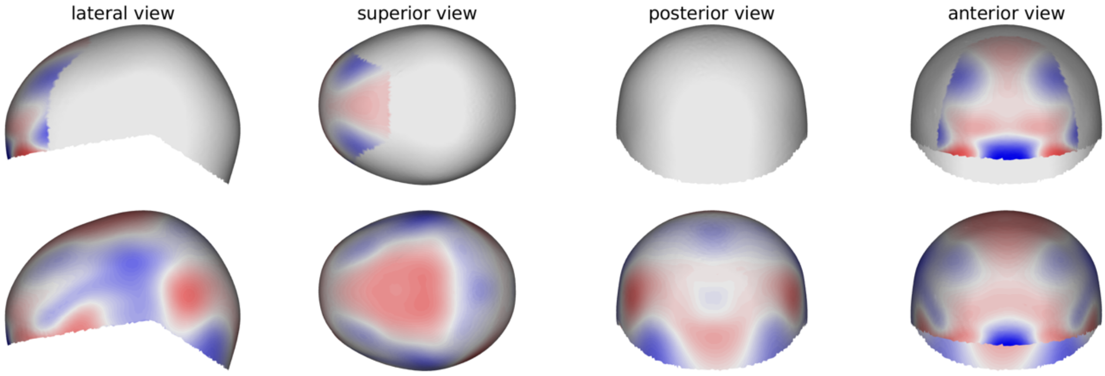

See page 2 for captions

rs7920484

A

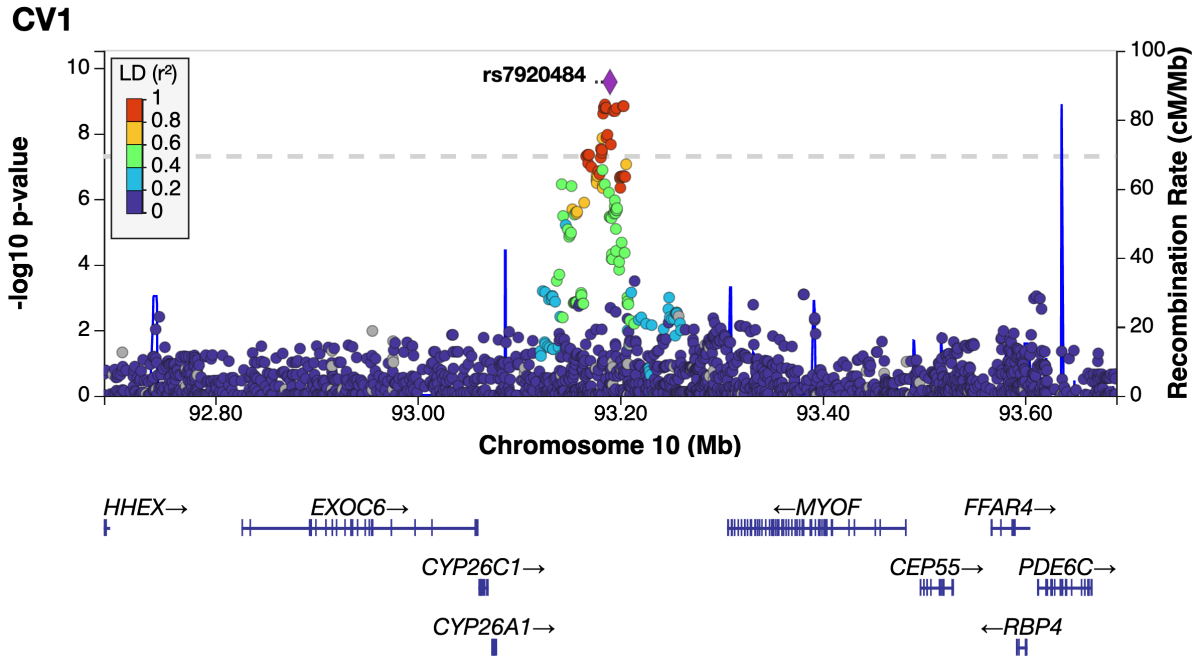

B

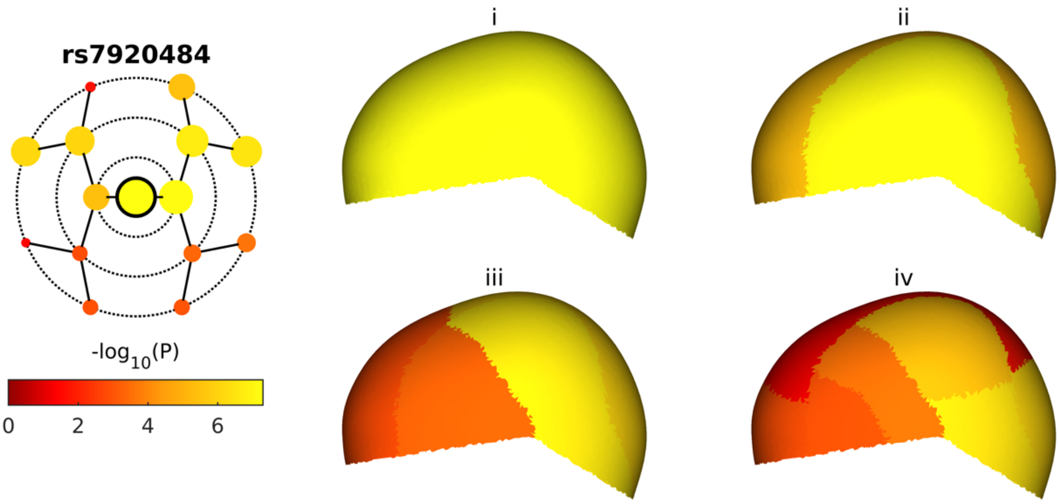

C

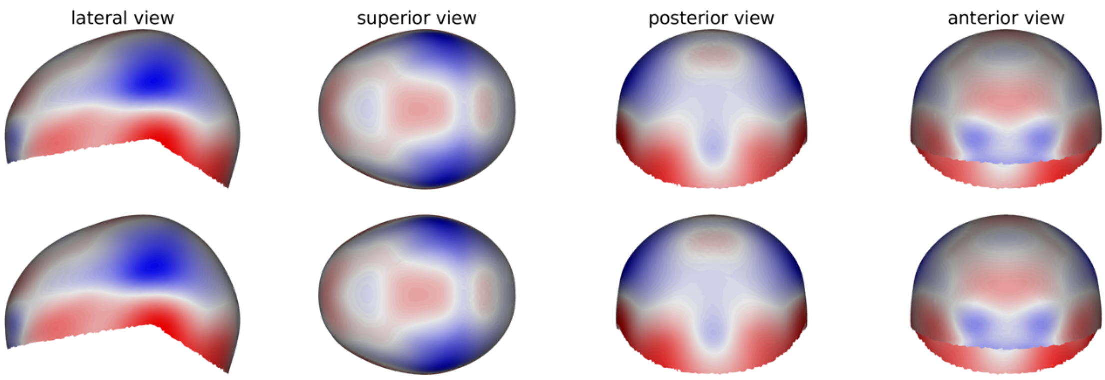

See page 2 for captions

rs61920200

A

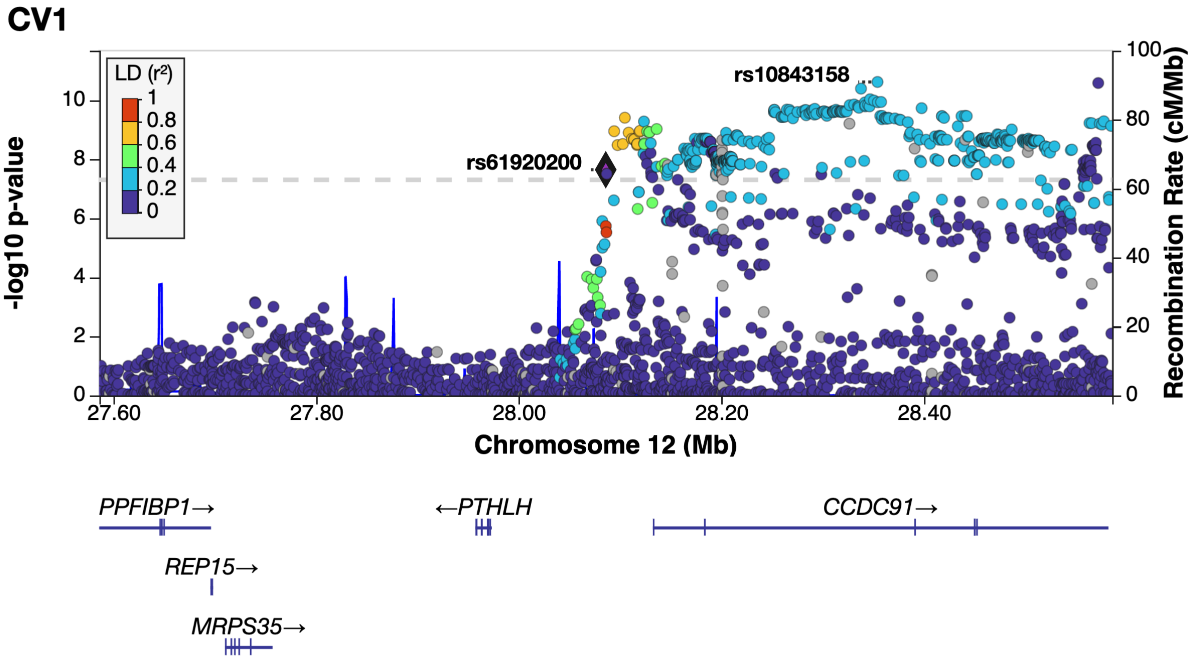

B

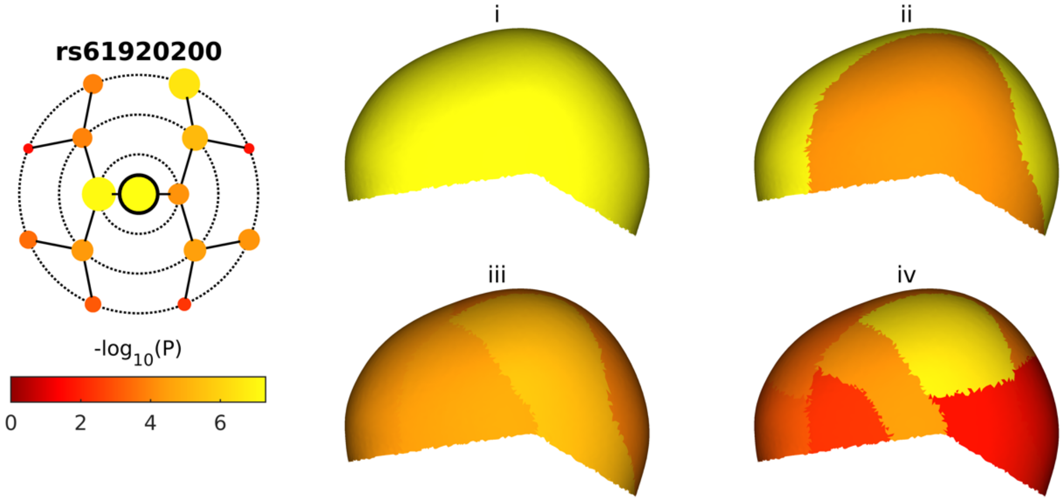

C

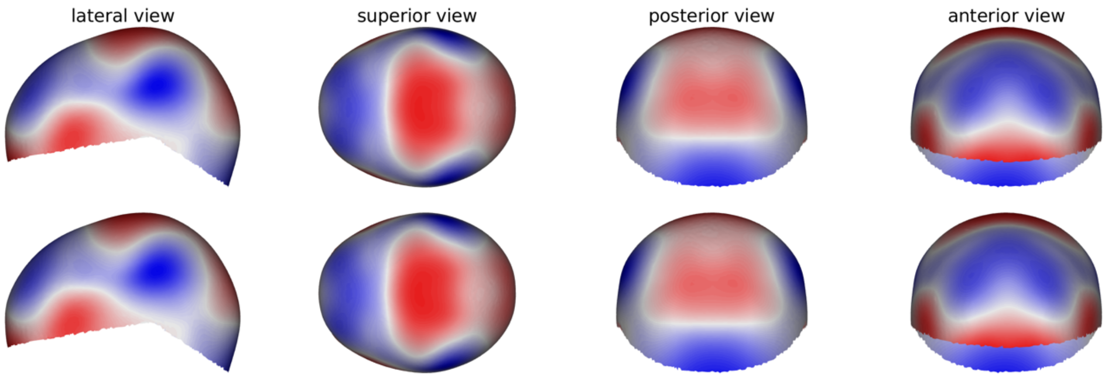

See page 2 for captions

rs10843158

A

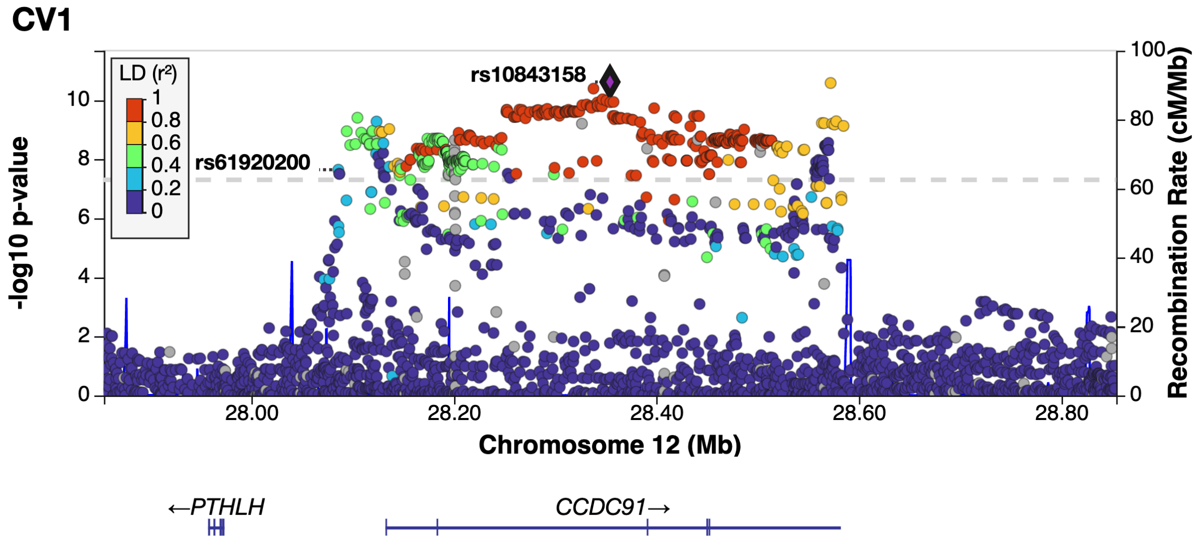

B

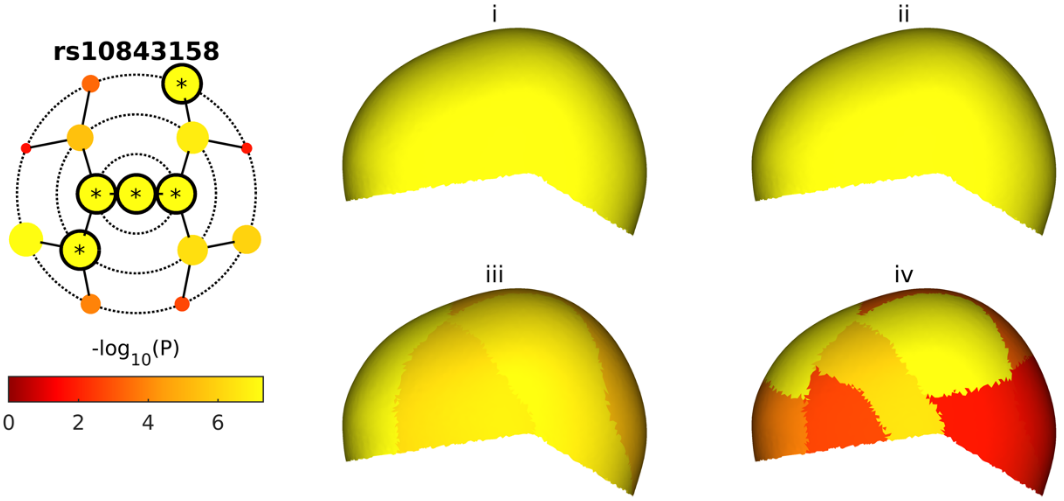

C

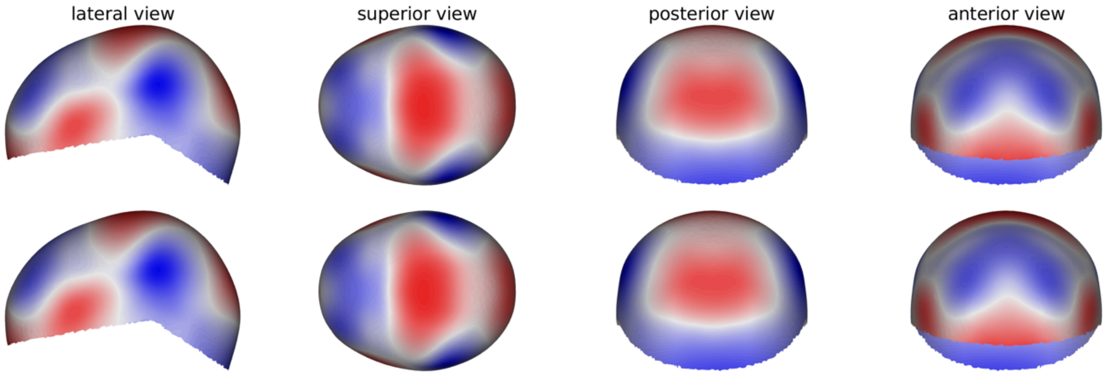

See page 2 for captions

rs151174669

A

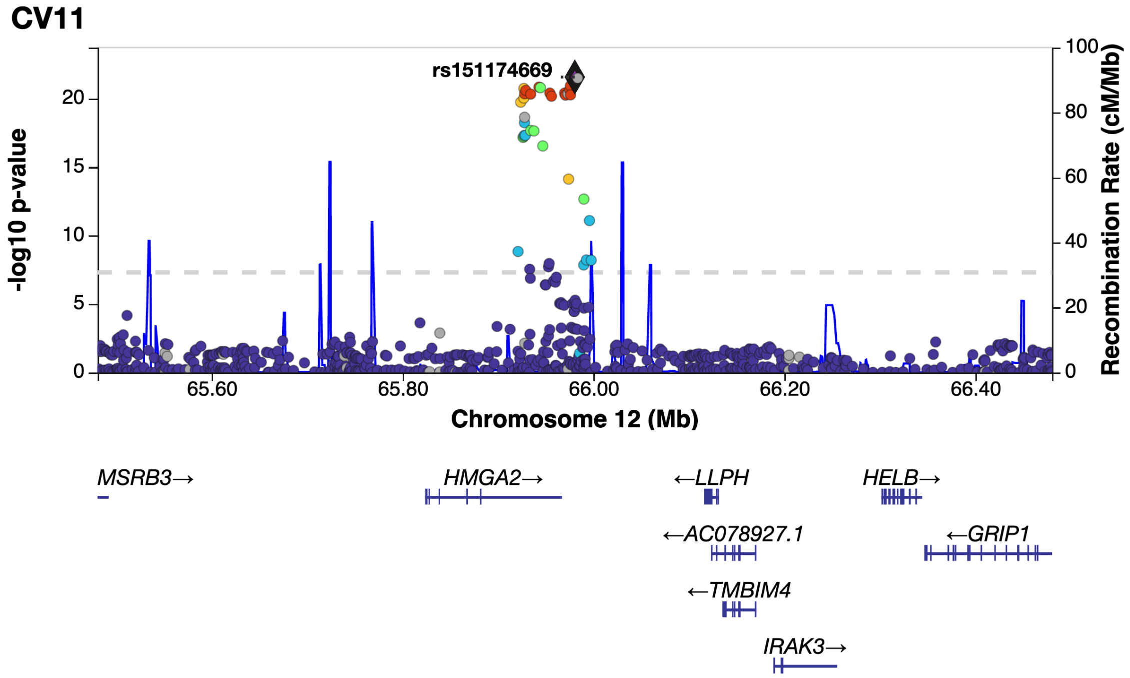

B

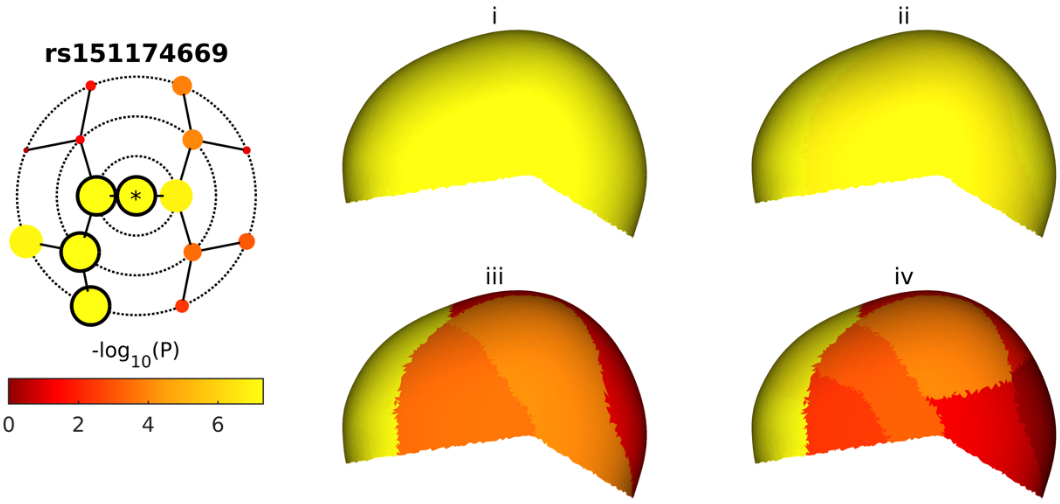

C

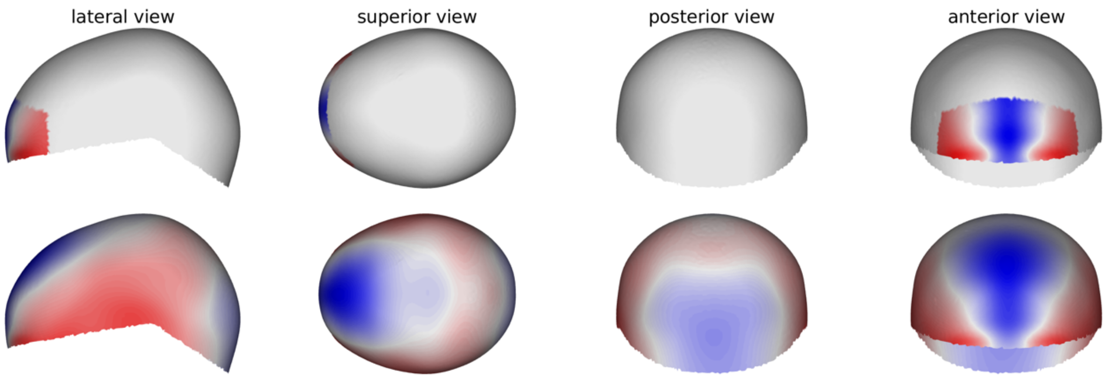

See page 2 for captions

rs11609649

A

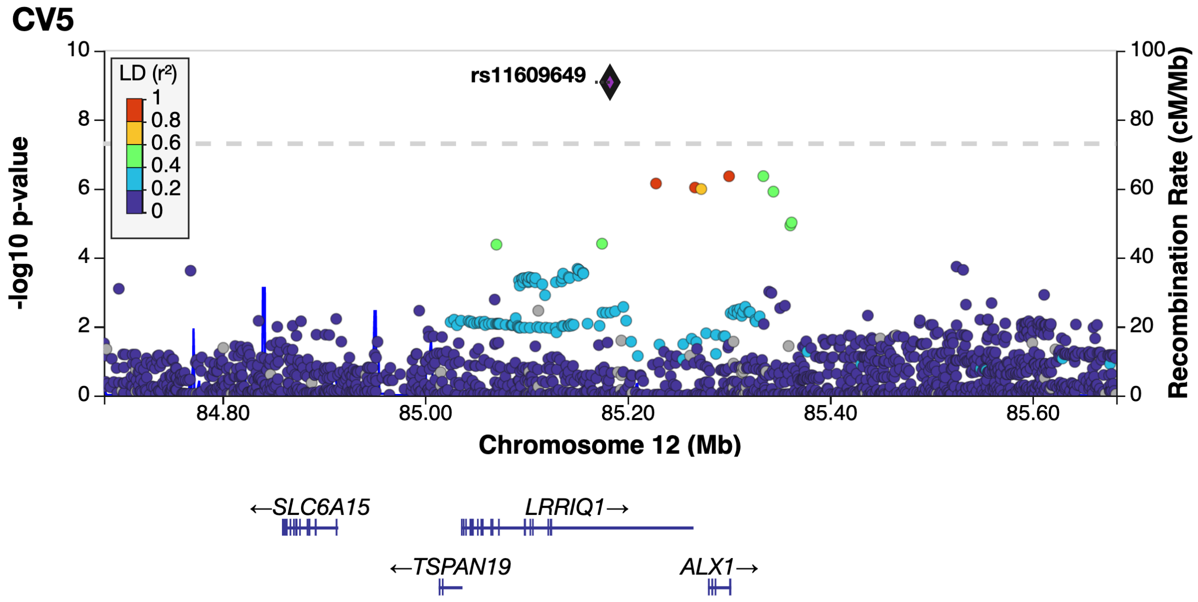

B

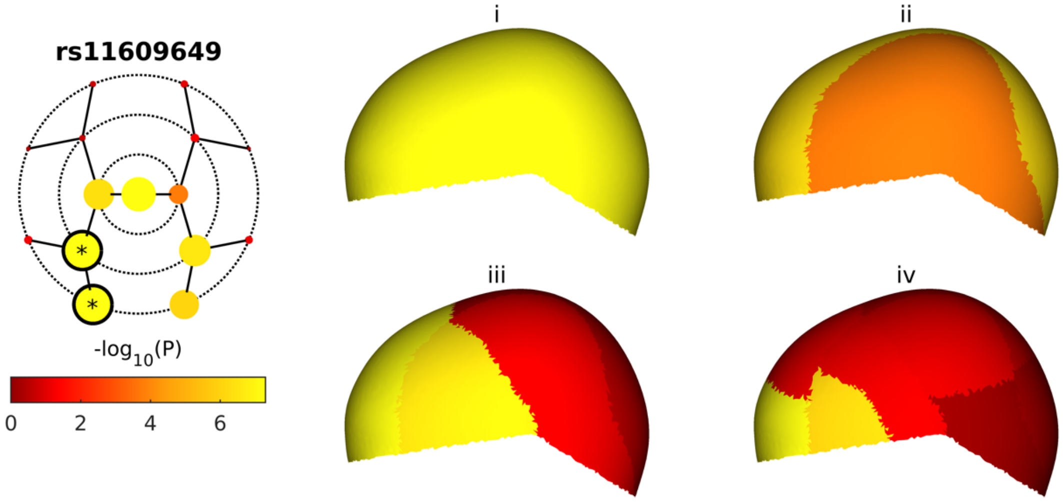

C

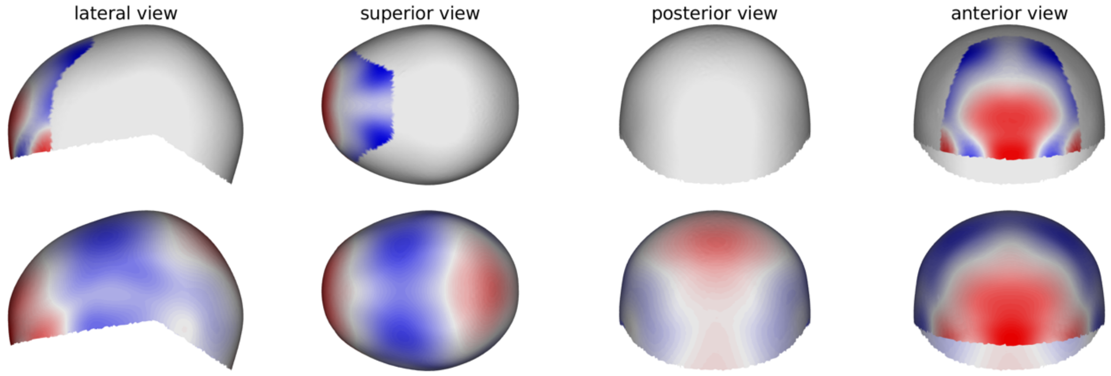

See page 2 for captions

rs1034266

A

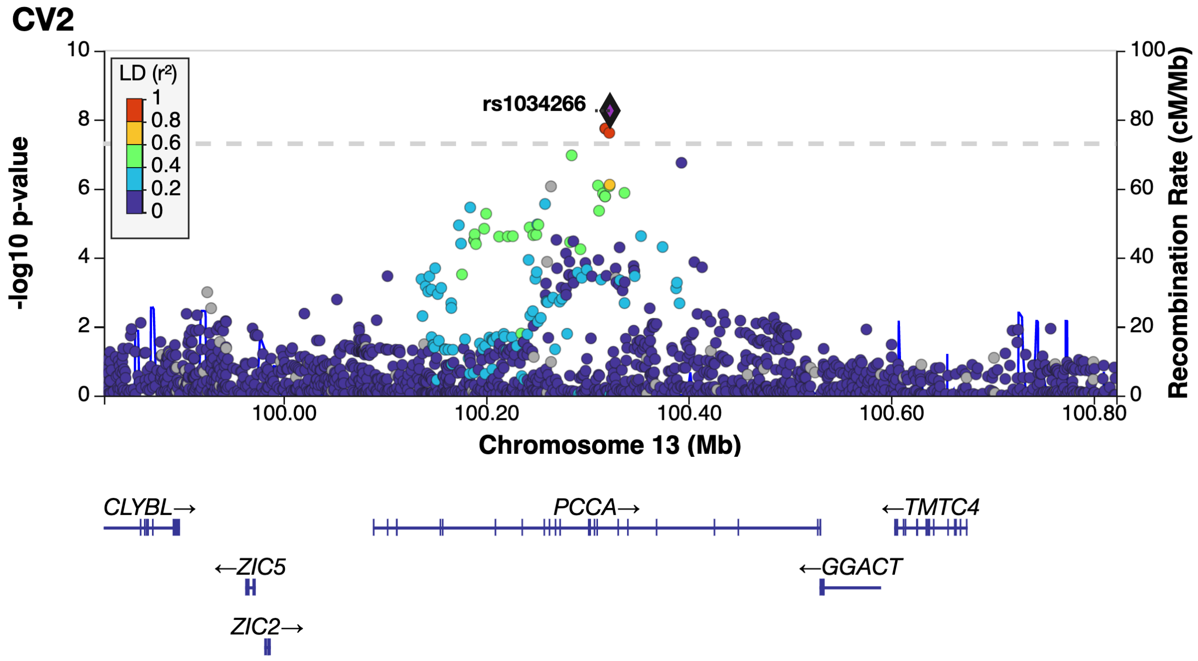

B

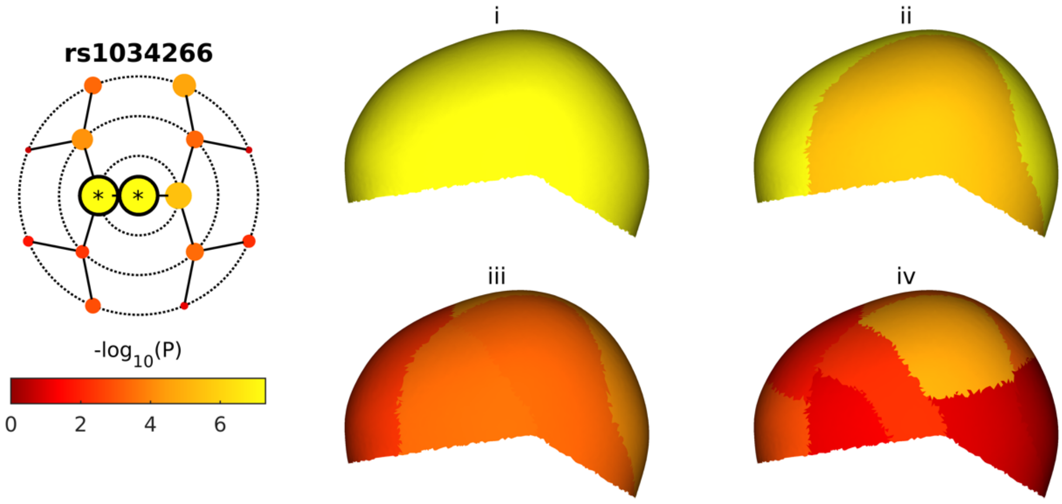

C

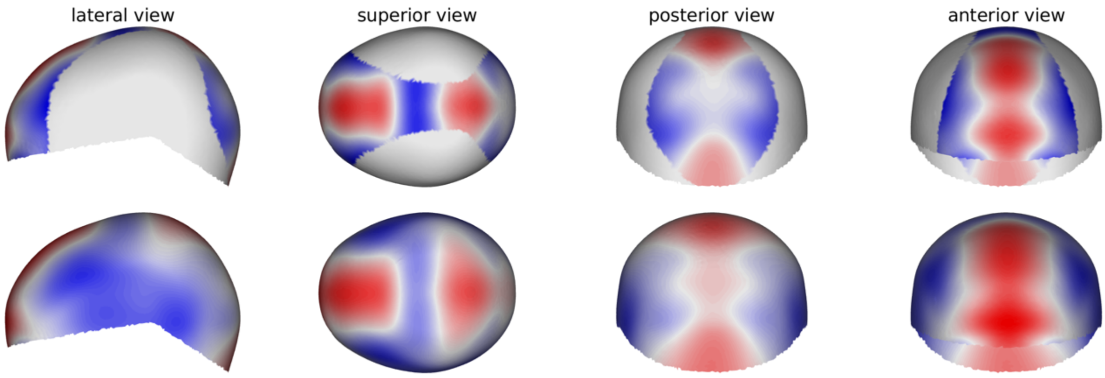

See page 2 for captions

rs1380208

A

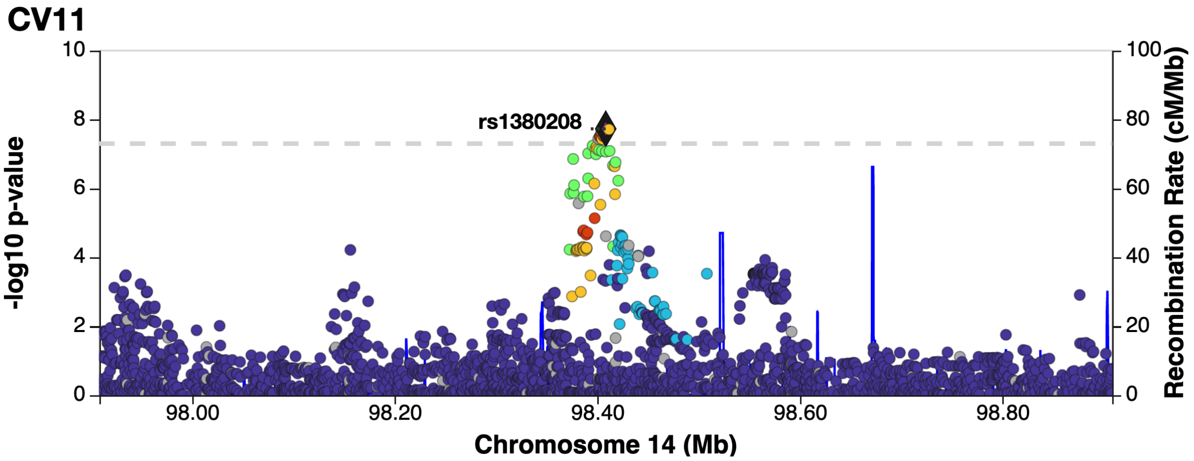

B

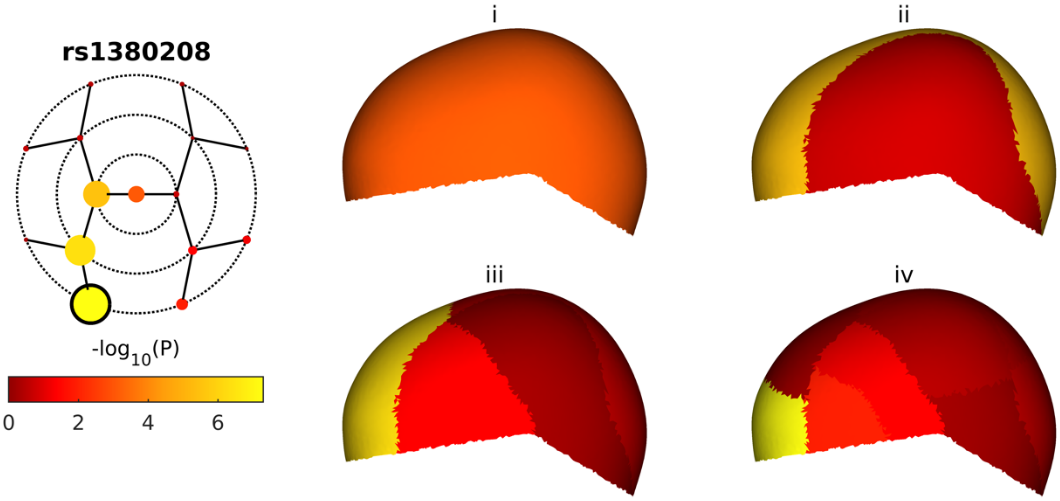

C

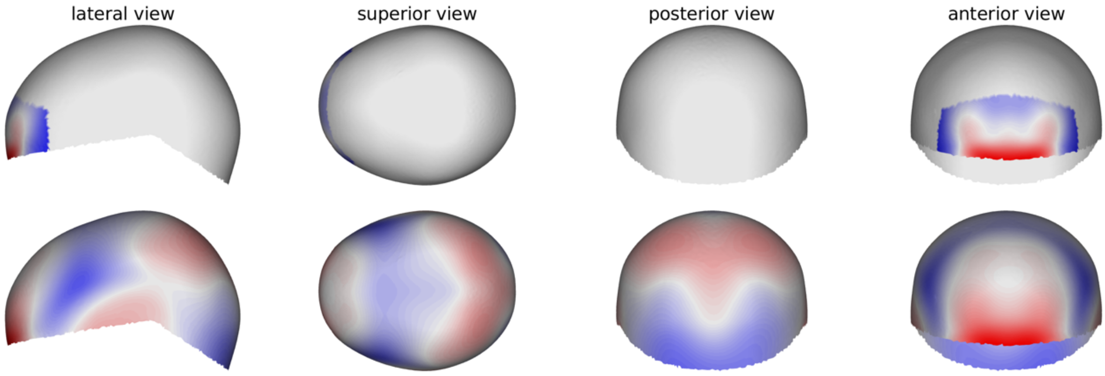

See page 2 for captions

rs4842918

A

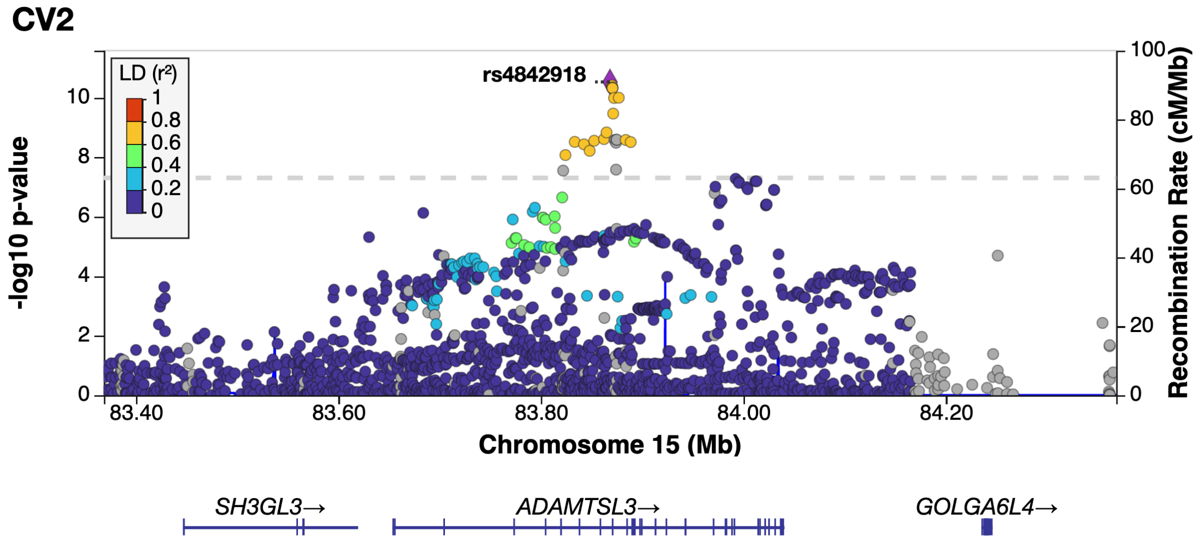

B

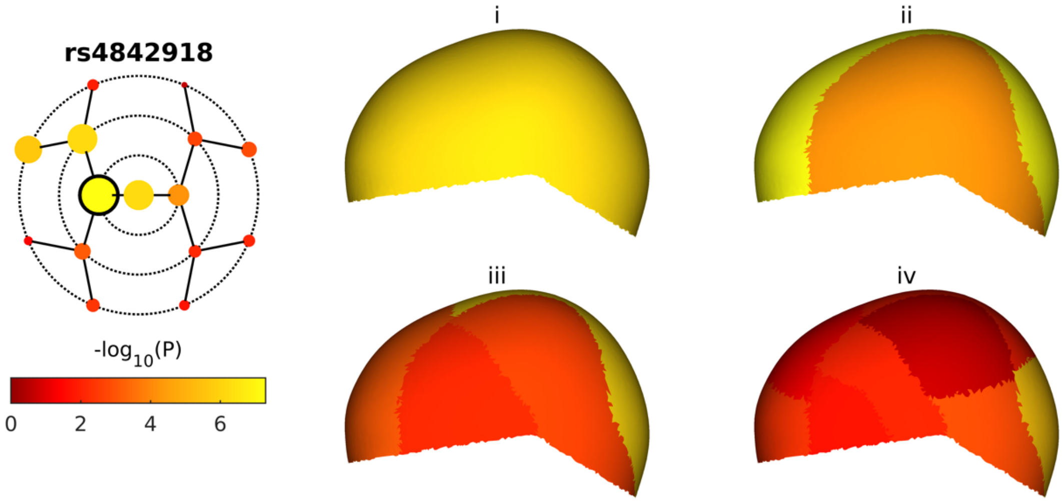

C

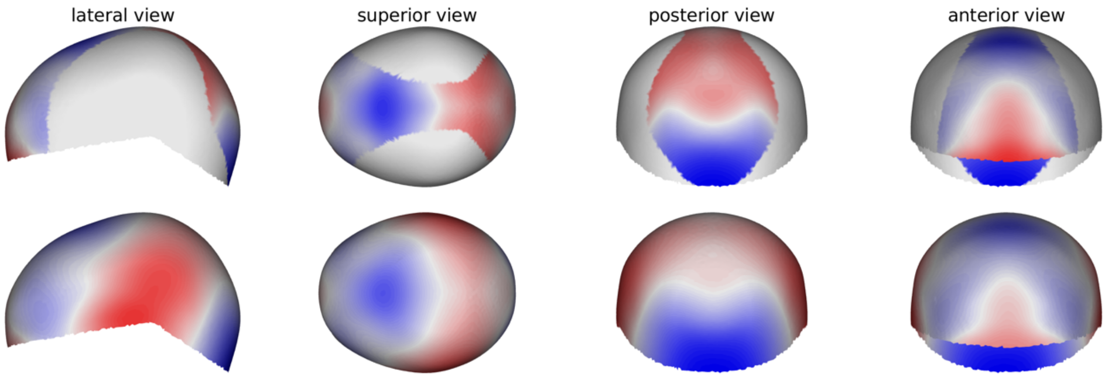

See page 2 for captions

rs12940346

A

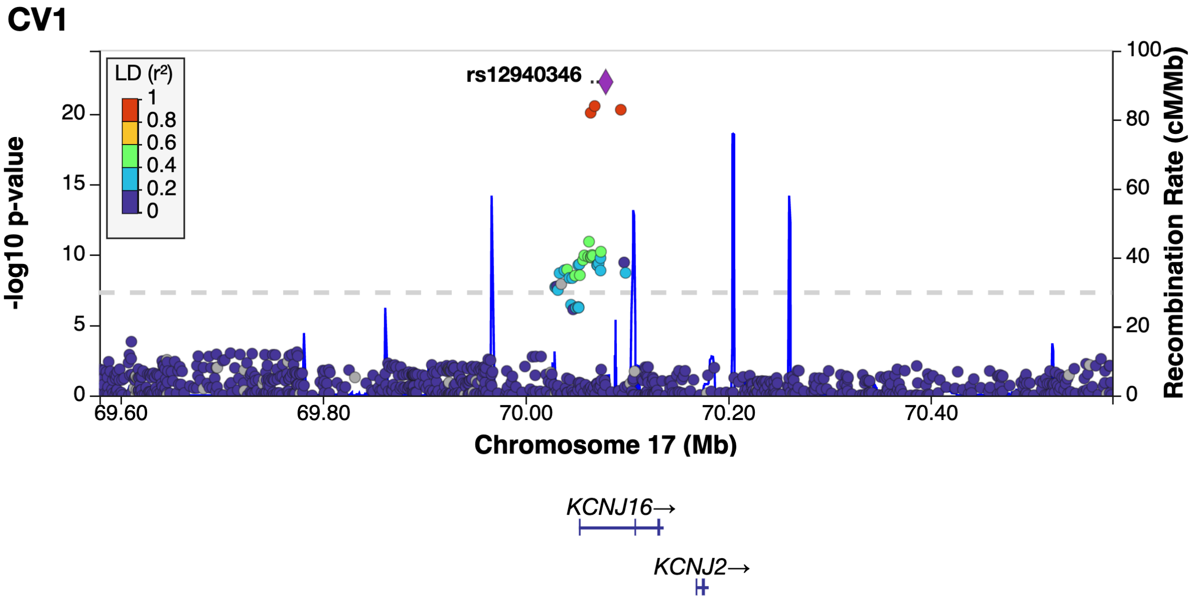

B

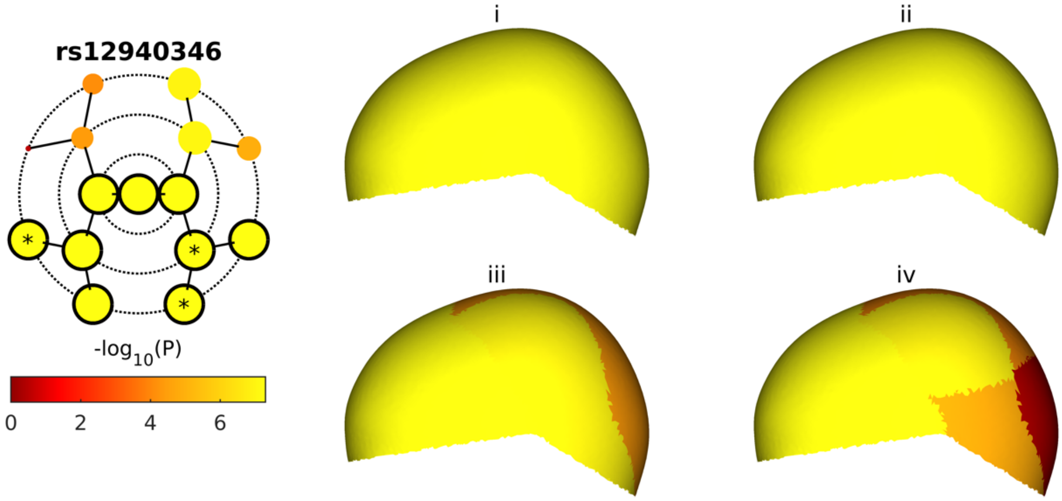

C

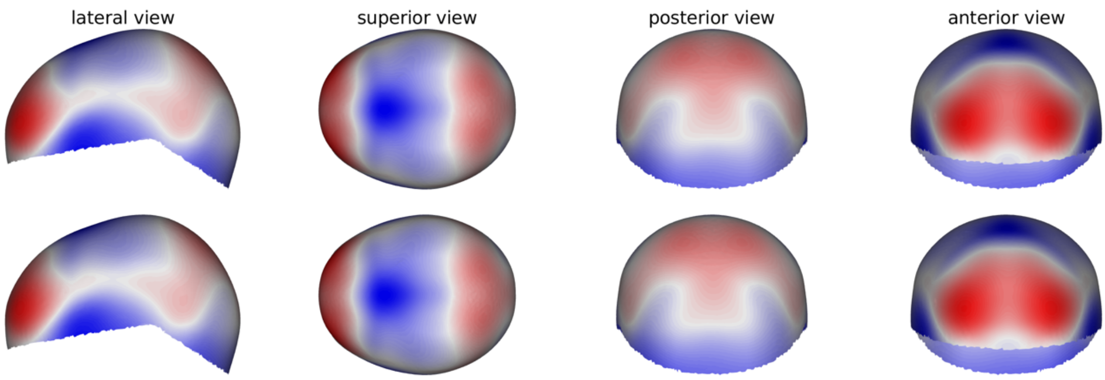

See page 2 for captions

rs1321454

A

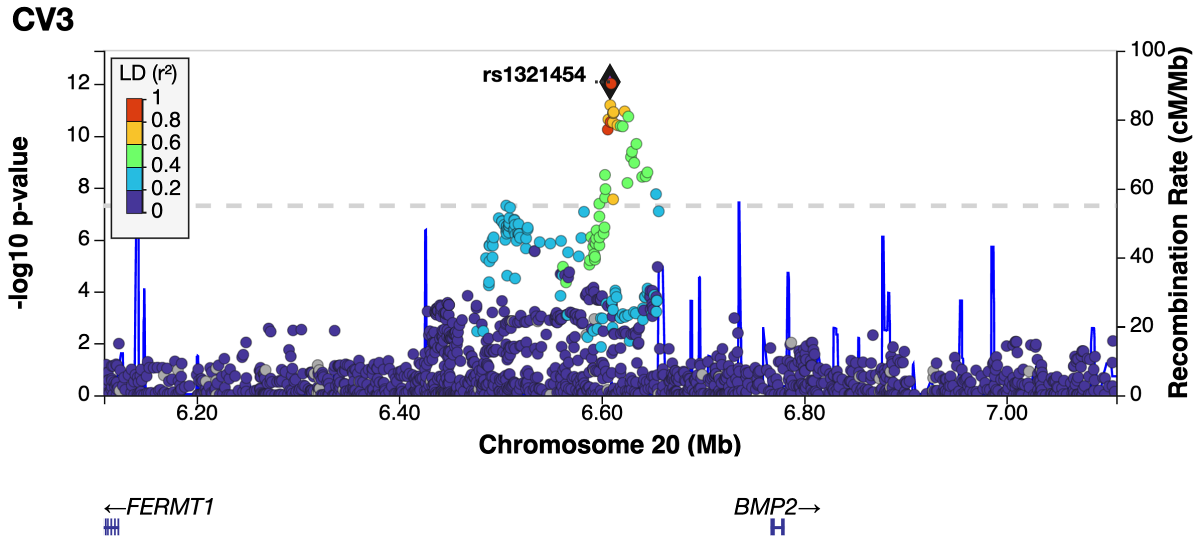

B

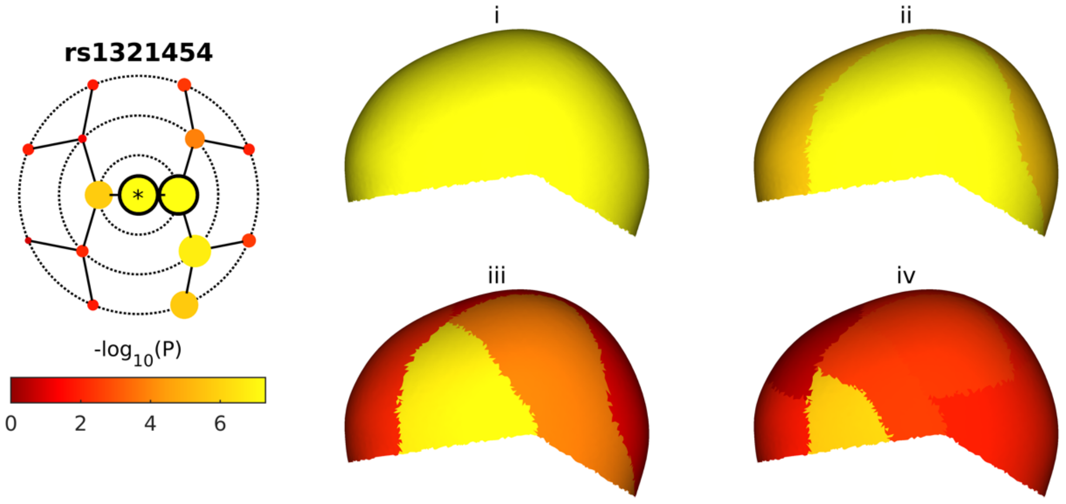

C

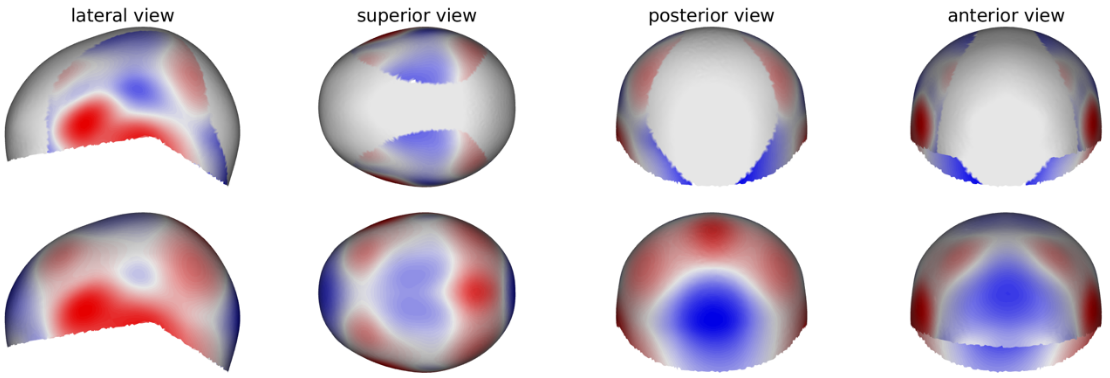

See page 2 for captions

rs6054748

A

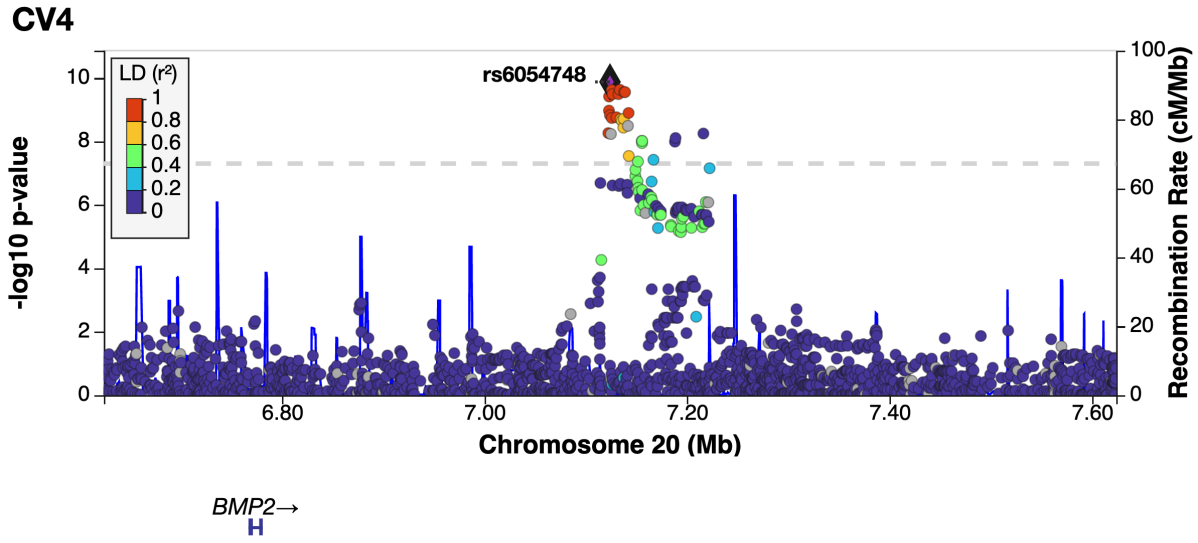

B

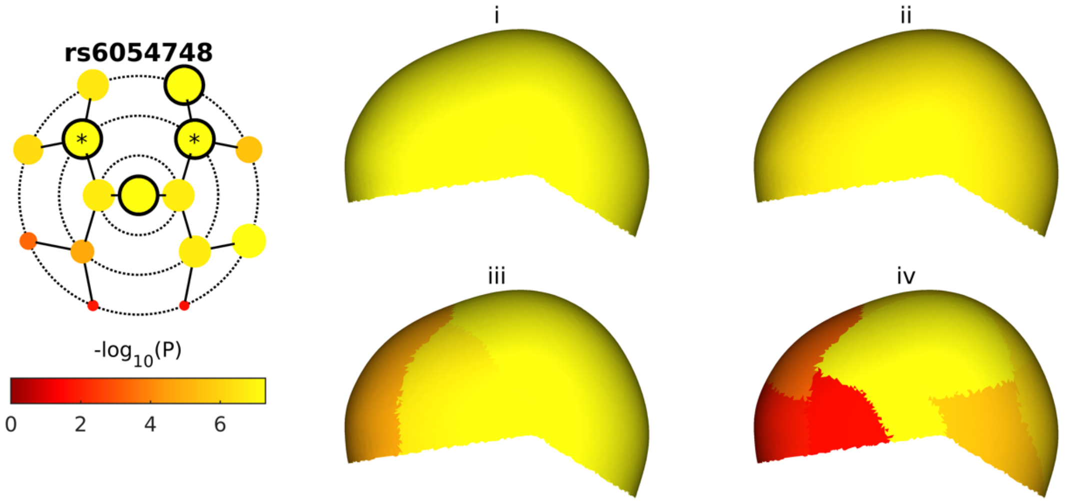

C

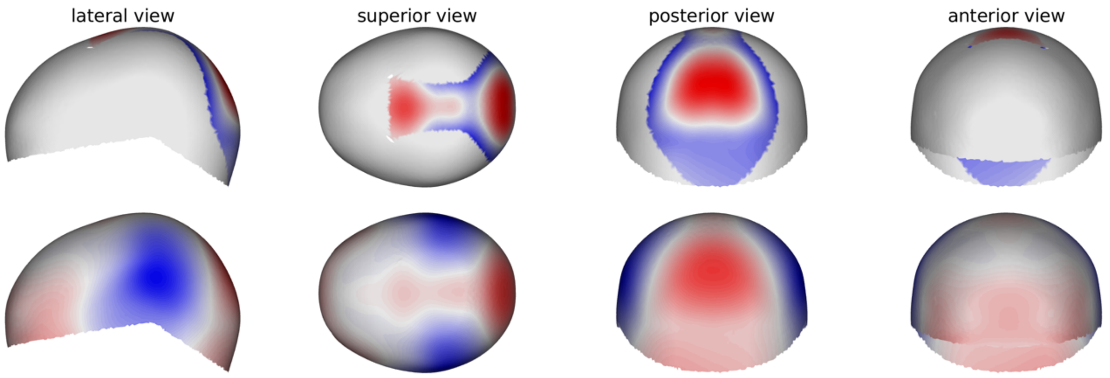

Supplement: Supplementary file 4 — Supplementary Data 1 [file 41467_2023_43237_MOESM4_ESM.pdf]
