# Supplementary material for: Joint multi-ancestry and admixed GWAS reveals the complex genetics behind human cranial vault shape
Source: Nat Commun. 2023 Nov 16;14:7436. doi: 10.1038/s41467-023-43237-8 (PMC10654897; doi:10.1038/s41467-023-43237-8)
Supplement: Supplementary file 5 — Supplementary Data 2 [file 41467_2023_43237_MOESM5_ESM.pdf]

Supplementary Information

Multi-ancestry versus European signals

S. Goovaerts et al.

Contents

RS3936018 – RS3936018 3

RS11126057 – RS2009778 3

RS6739488 – RS6739488 3

RS17479393 – RS17479393 4

RS970797 – RS970797 4

RS7630713 – RS7626244 4

RS13083115 – RS35614773 5

RS62368491 – RS1351637 5

RS3822730 – RS3822730 5

RS9367014 – RS4714260 6

RS3799974 – RS3799970 6

RS6902885 – RS9491697 6

RS498079 – RS296418 7

RS17396870 – RS148673350 7

RS202055590 – RS202055590 7

RS34275932 – RS1581525 8

RS144986754 – RS147676525 8

RS9297414 – RS7813717 8

RS7038482 – RS10120728 9

RS72815155 – RS7920484 9

RS563186113 – RS74976049 9

RS11049359 – RS61920200 10

RS11049359 – RS10843158 10

RS11175968 – RS151174669 10

RS11609649 – RS11609649 11

RS7981883 – RS1034266 11

RS1531307 – RS1380208 11

RS55876638 – RS4842918 12

RS12940346 – RS12940346 12

RS6038571 – RS1321454 12

RS1884302 – RS6054748 13

See page 2 for captions

Comparison of multi-ancestry and European shape associations at the joint set of loci. A, Shape associated with lead SNPs from the full multi-ancestry GWAS (FULL; n = 6,772) and European subset GWAS (EUR; n = 4,198). Bold rsID labels indicate that the SNP was a lead SNP in the respective GWAS; regular type rsID labels indicate that the SNP was not genome-wide significant but had the lowest P-value among all SNPs within 200kb or in LD ($r^2 > 0.2$). Blue and red indicate inward depression and outward protrusion respectively. **B,** -log10(P-values) (one-sided chi-square) across hierarchical cranial vault segments. Genome-wide significant (P < 5e-8) segments are indicated with a black circle. The strongest association across all segments is indicated with '*'. **C,** LocusCompare[1] plot of minimal P-values across hierarchical segments. Colors indicate LD with the most significant SNP across the FULL and EUR GWAS (purple diamond). If that SNP was not found in the 1000 Genomes dataset, the next most significant SNP was chosen. **D,** LocusZoom[2] plots of minimal P-values across hierarchical segments. Panel legend matches with **C**.

See page 2 for captions

rs3936018 – rs3936018

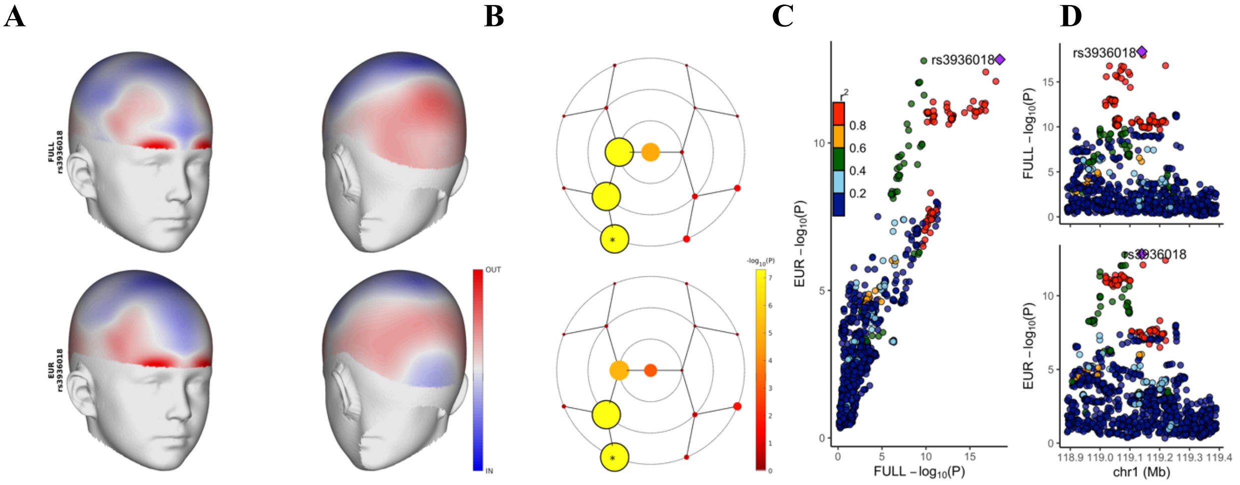

rs11126057 – rs2009778

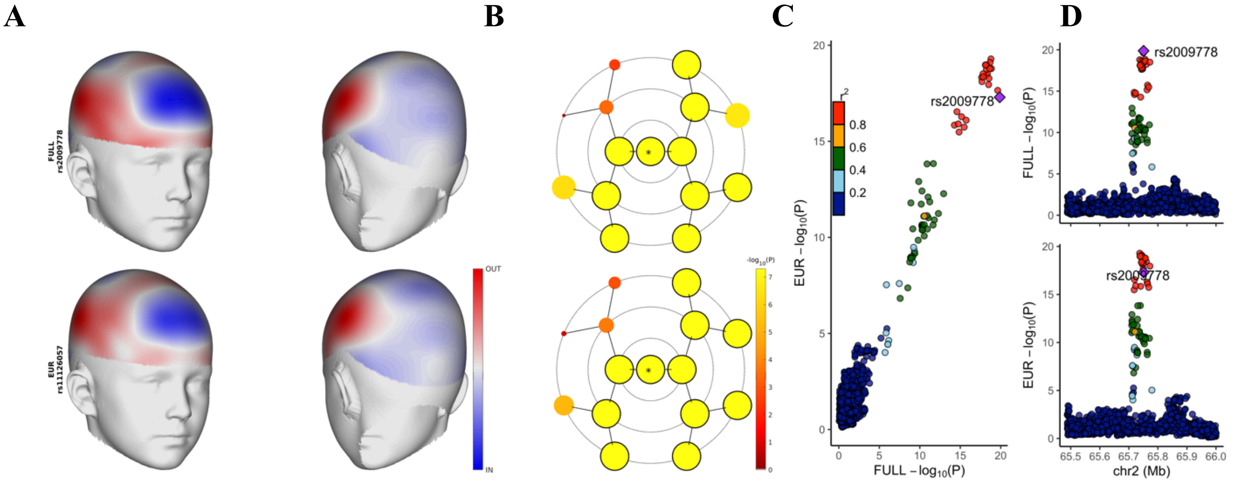

rs6739488 – rs6739488

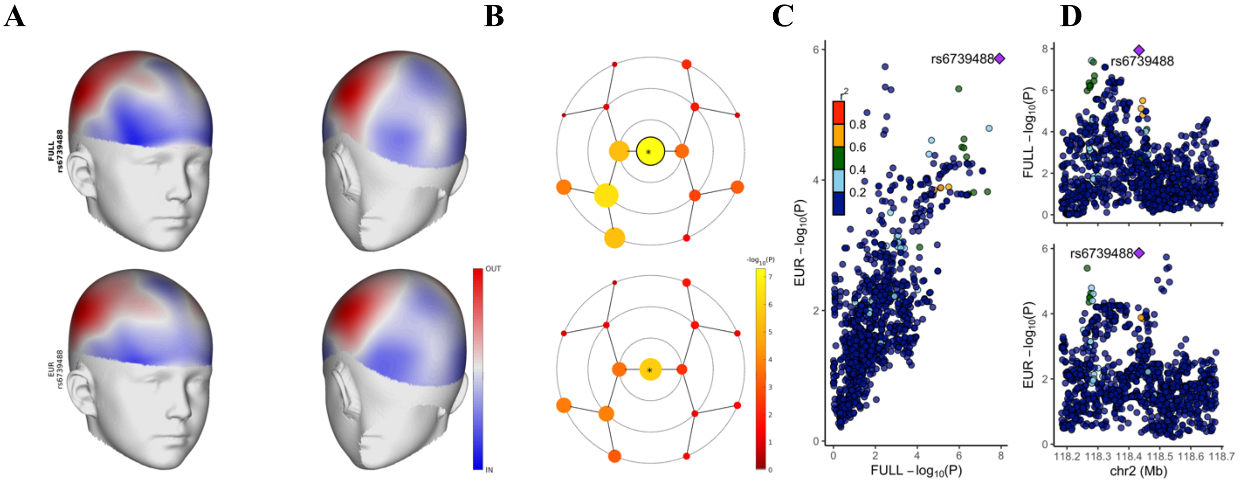

See page 2 for captions

rs17479393 – rs17479393

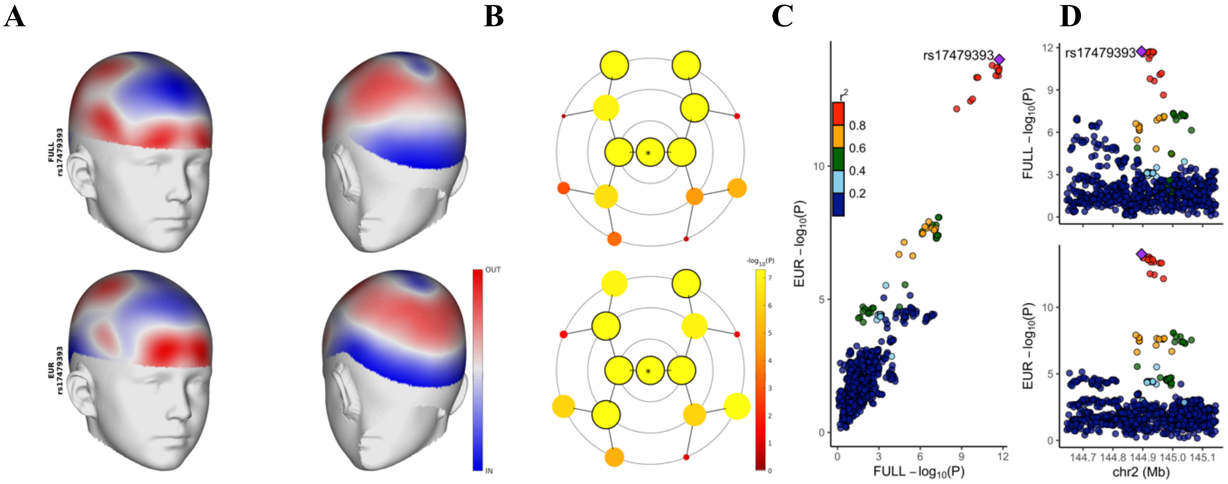

rs970797 – rs970797

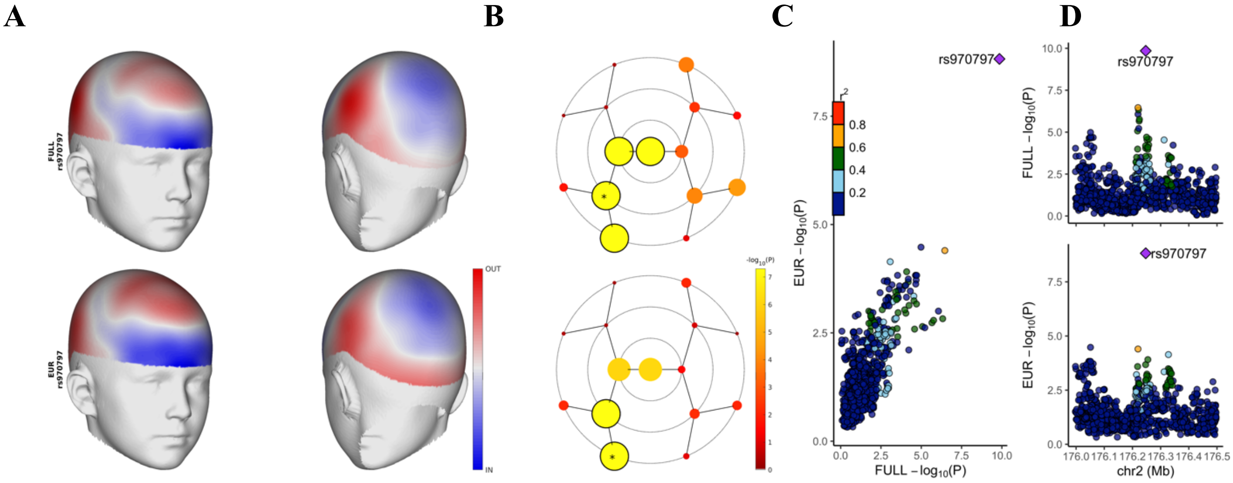

rs7630713 – rs7626244

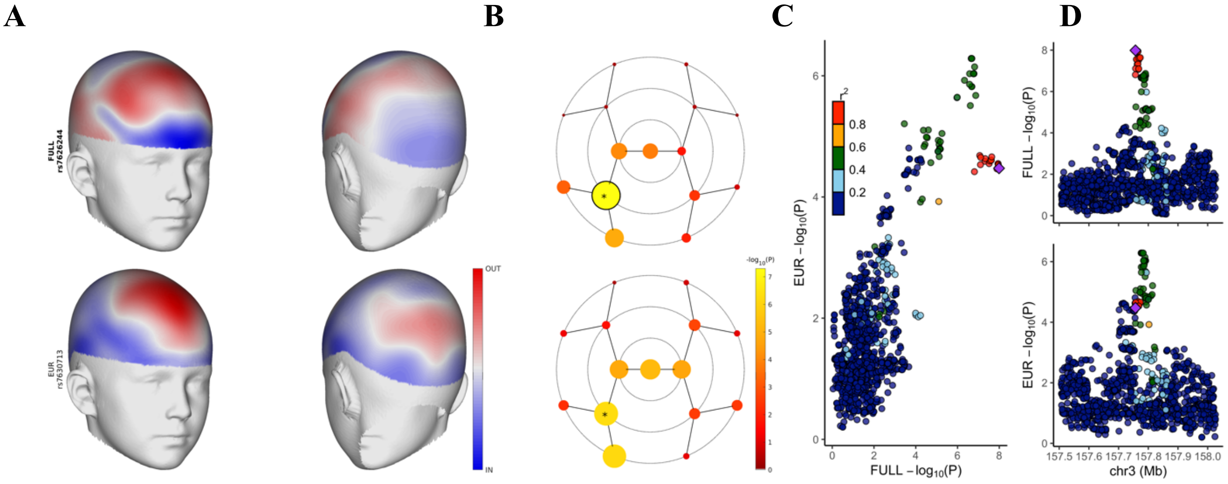

See page 2 for captions

rs13083115 – rs35614773

A **B** **C** **D**

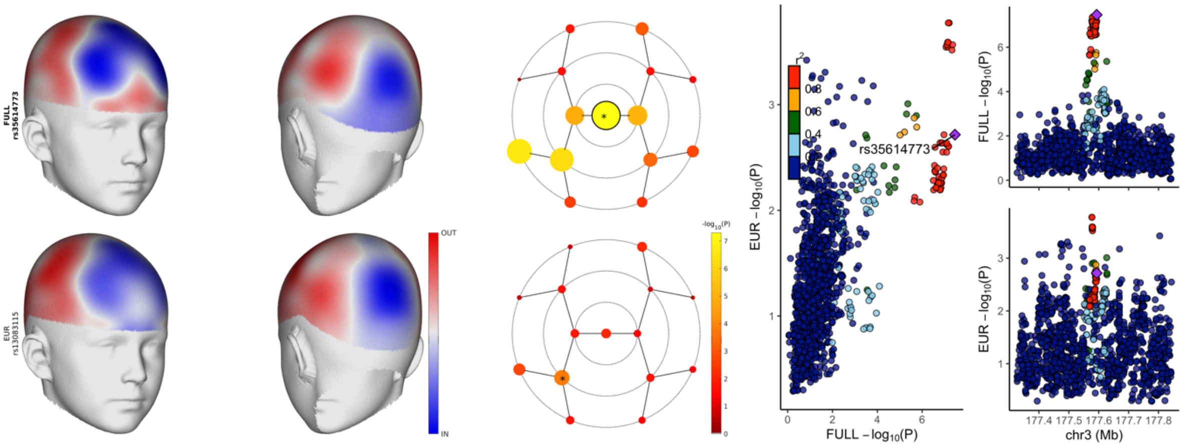

rs62368491 – rs1351637

A **B** **C** **D**

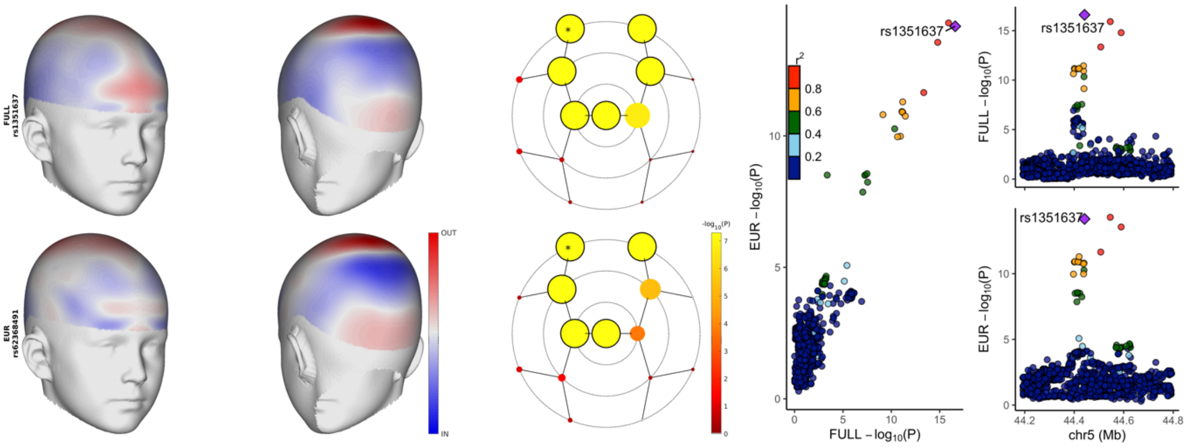

rs3822730 – rs3822730

A **B** **C** **D**

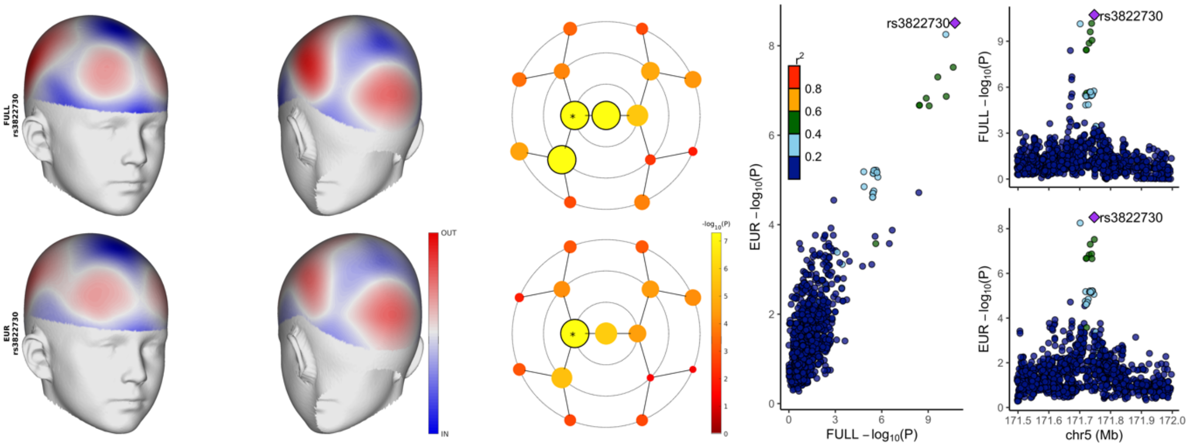

See page 2 for captions

rs9367014 – rs4714260

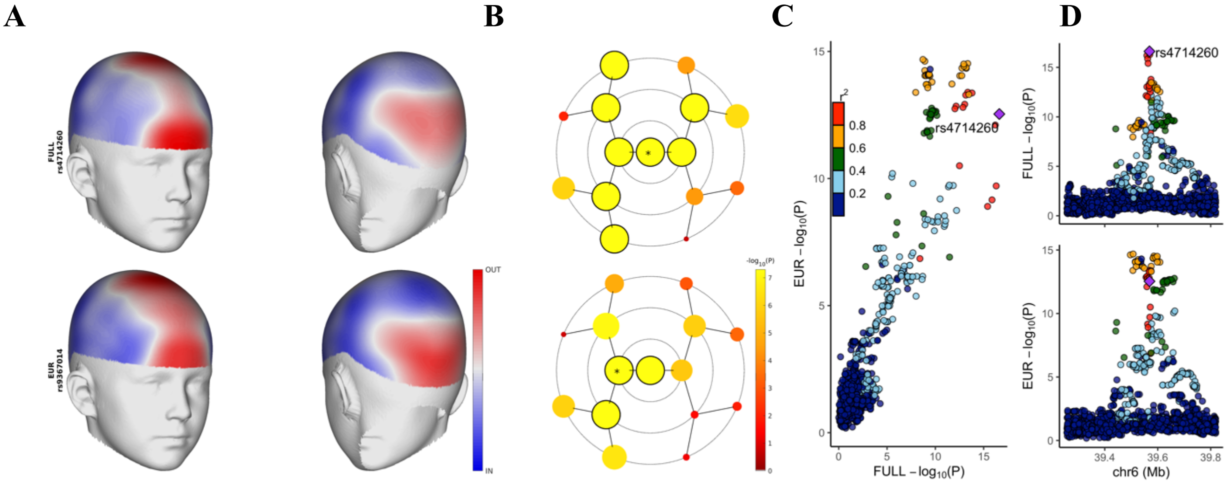

rs3799974 – rs3799970

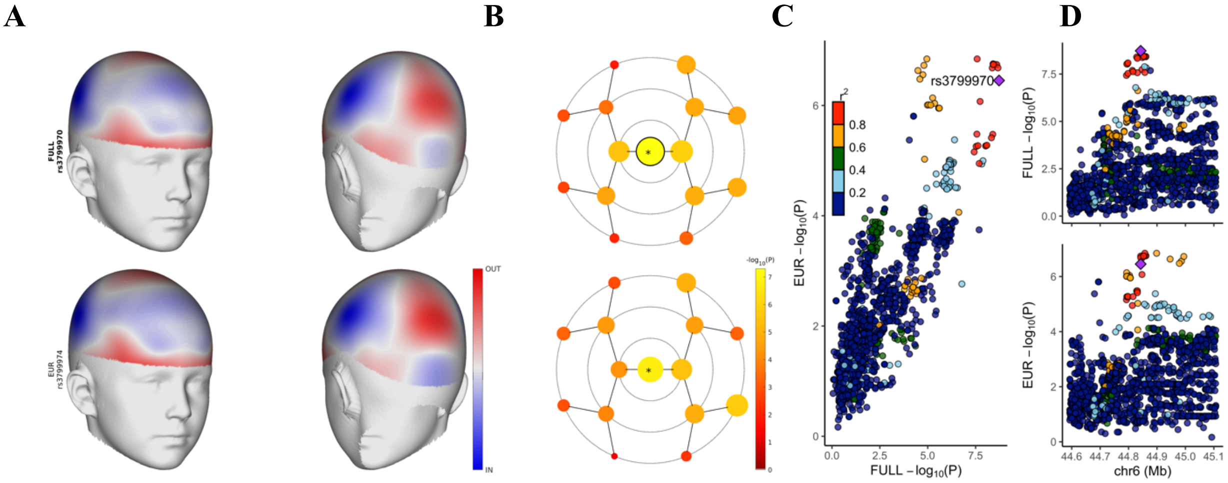

rs6902885 – rs9491697

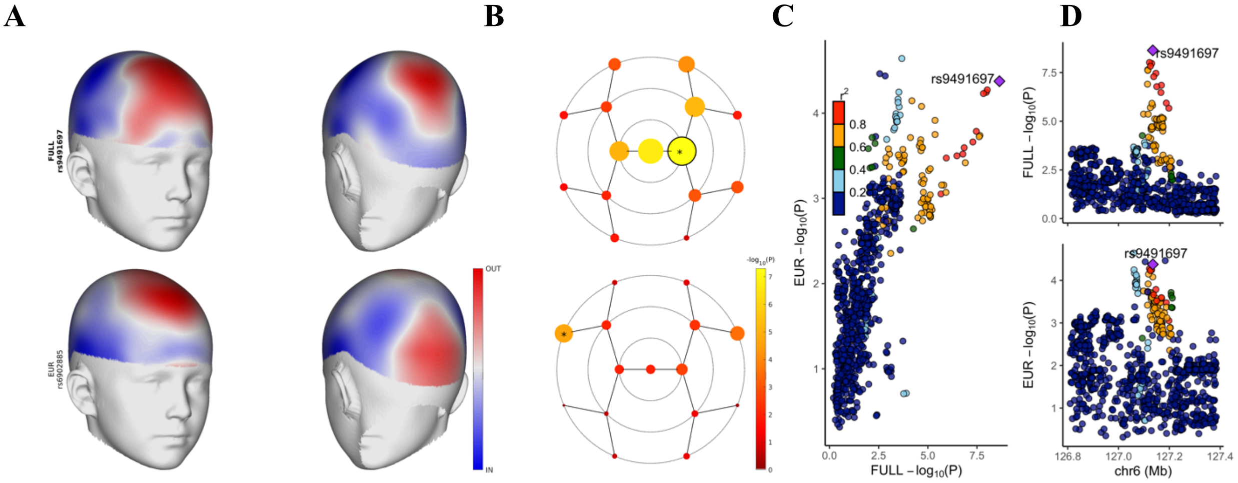

See page 2 for captions

rs498079 – rs296418

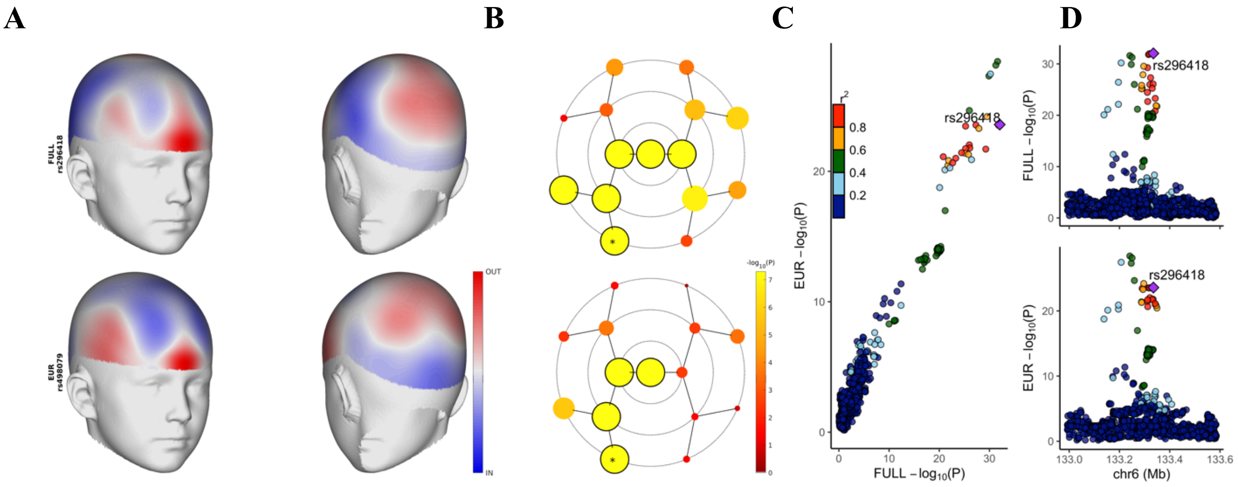

rs17396870 – rs148673350

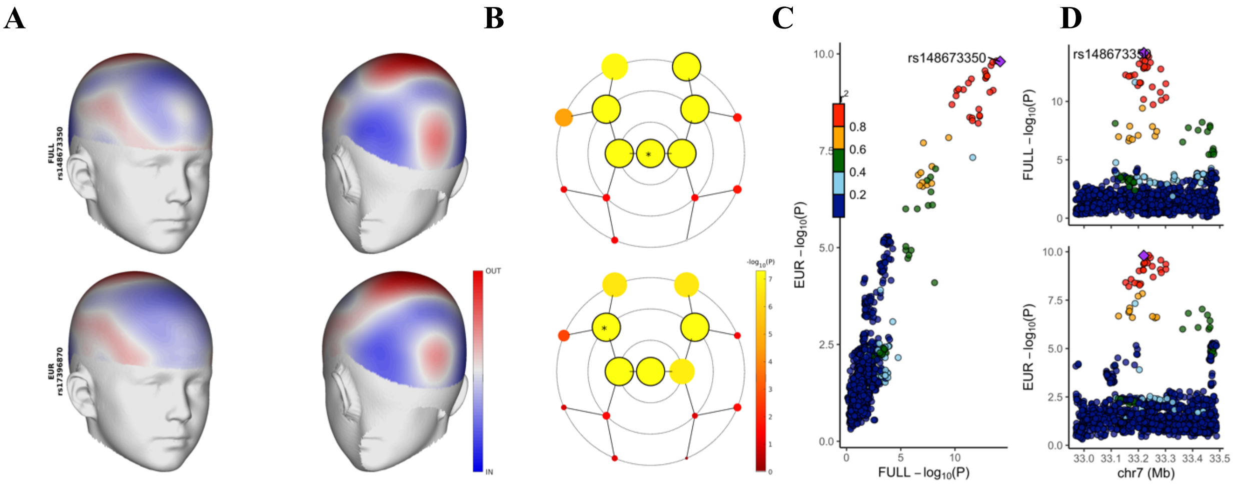

rs202055590 – rs202055590

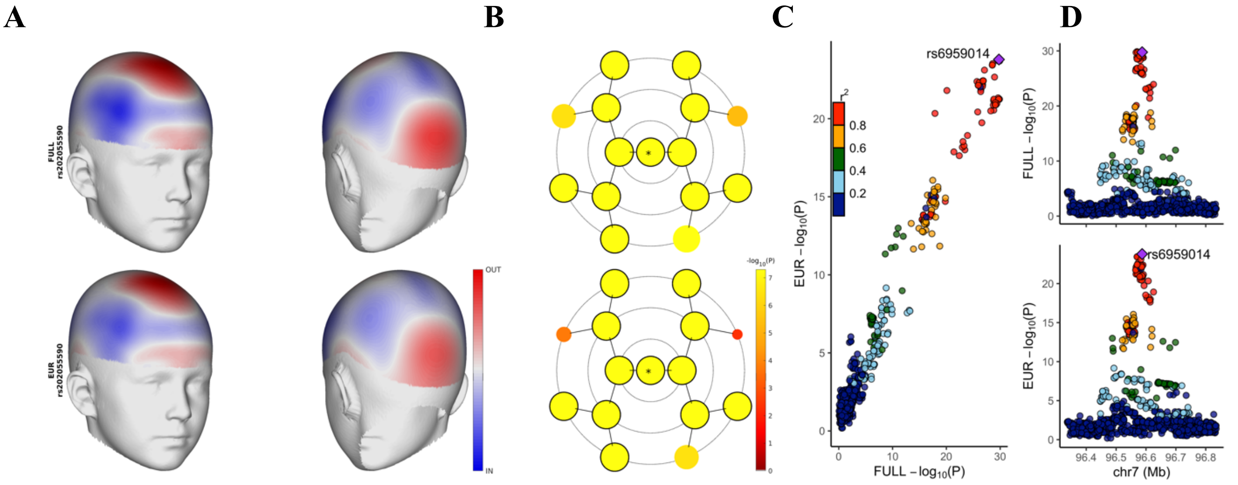

See page 2 for captions

rs34275932 – rs1581525

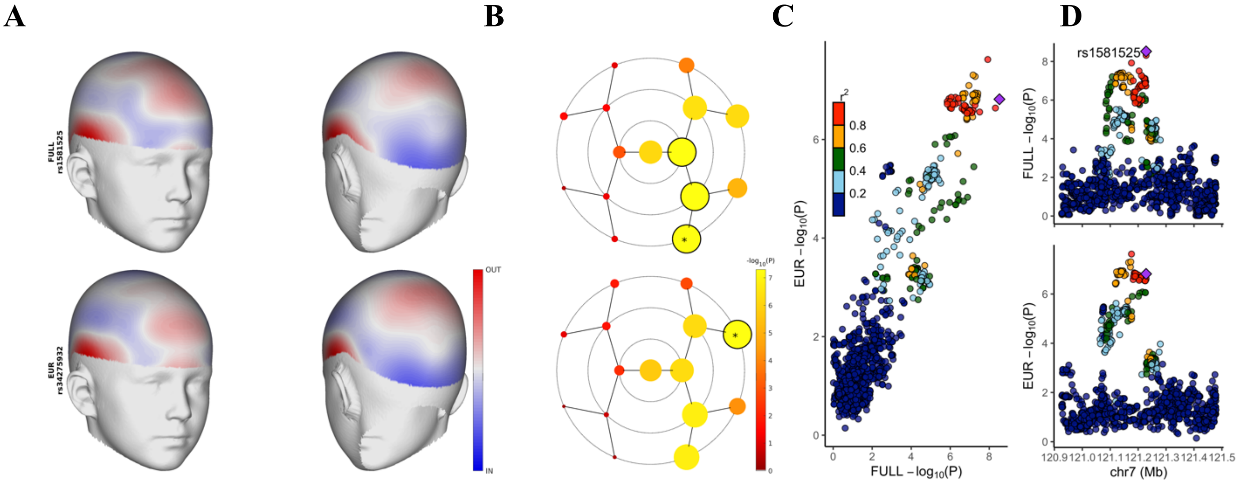

rs144986754 – rs147676525

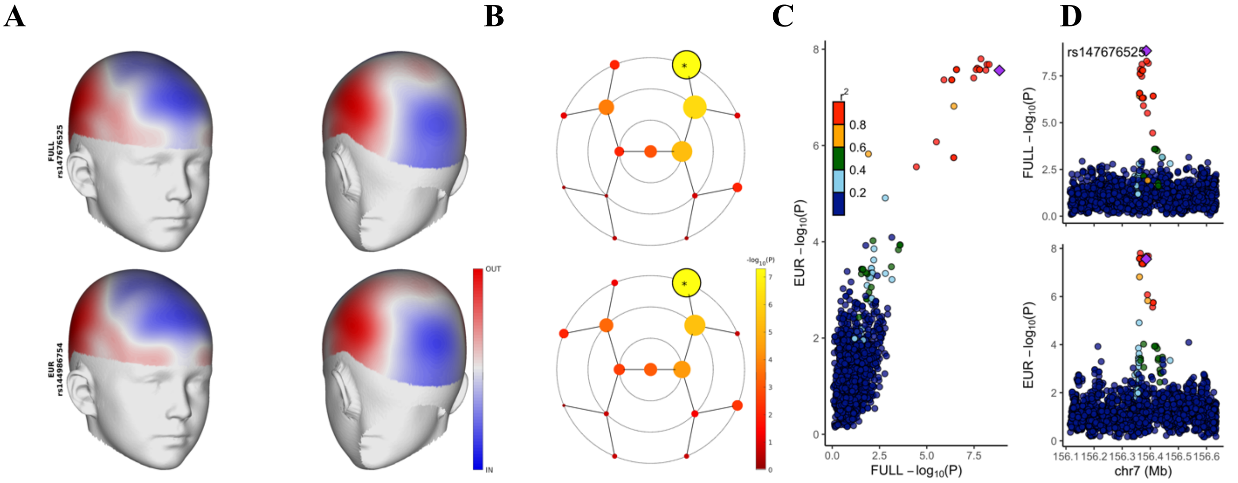

rs9297414 – rs7813717

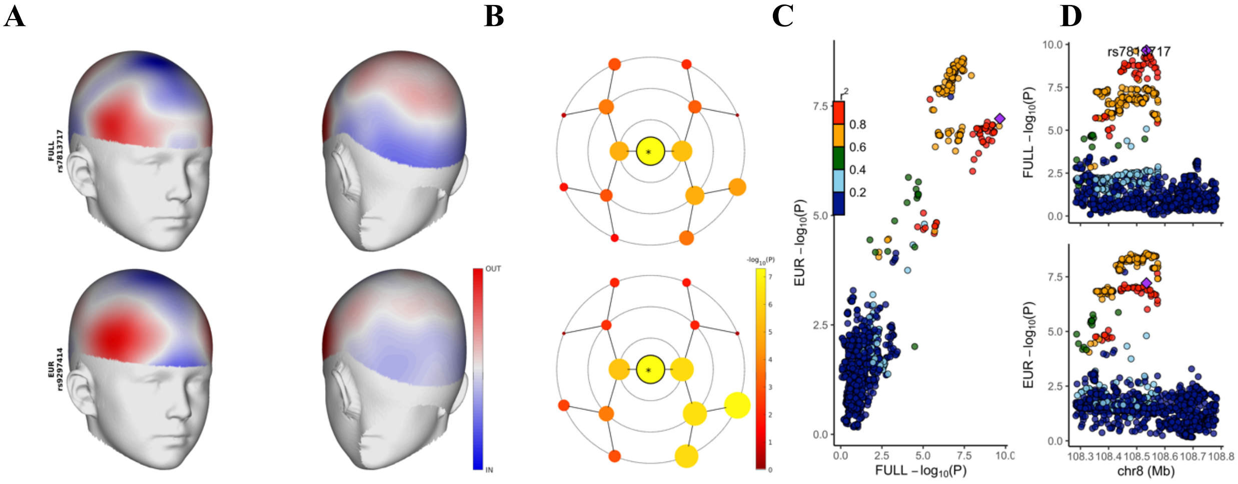

See page 2 for captions

rs7038482 – rs10120728

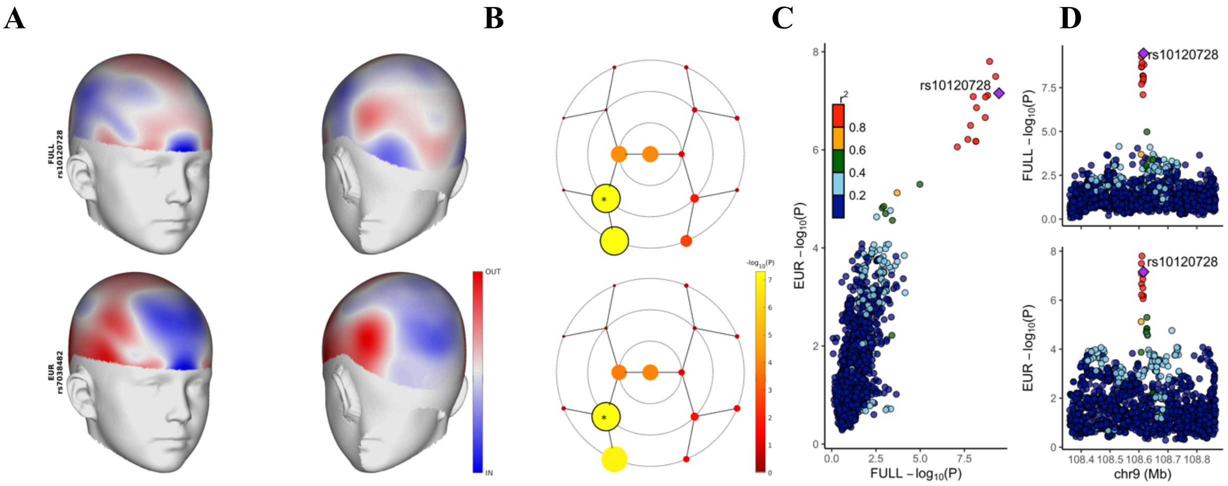

rs72815155 – rs7920484

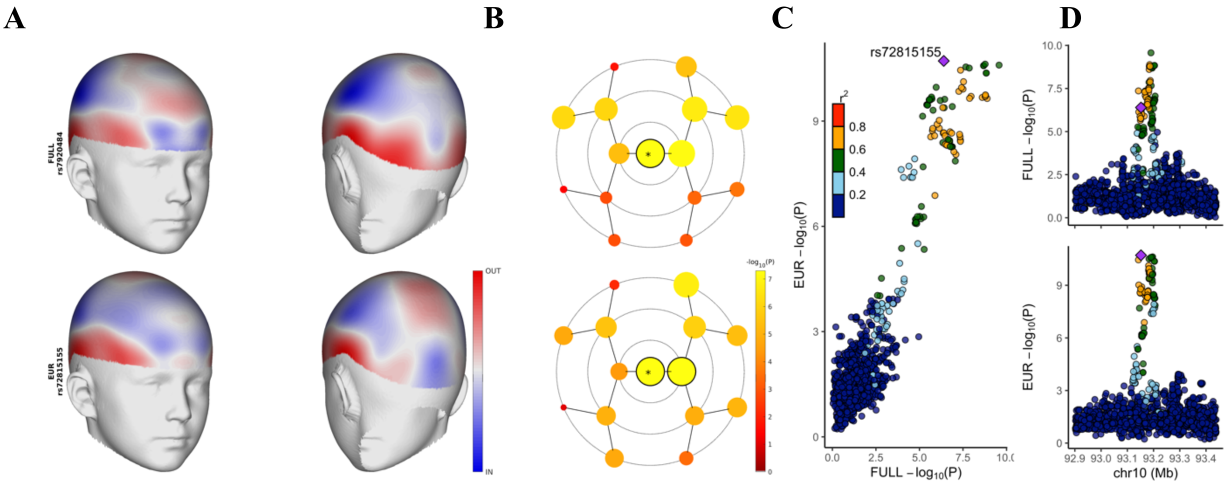

rs563186113 – rs74976049

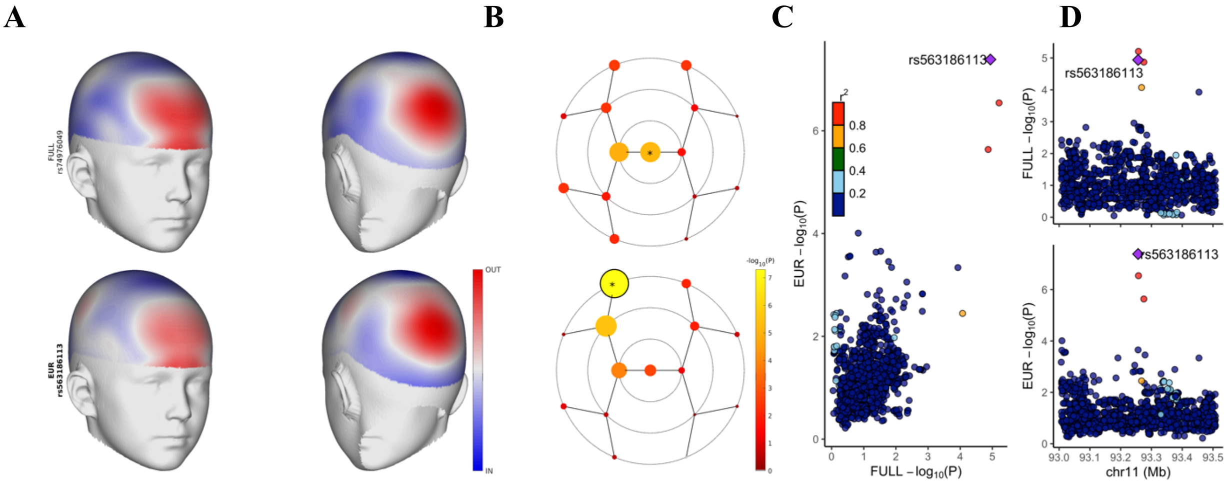

See page 2 for captions

rs11049359 – rs61920200

A B C D

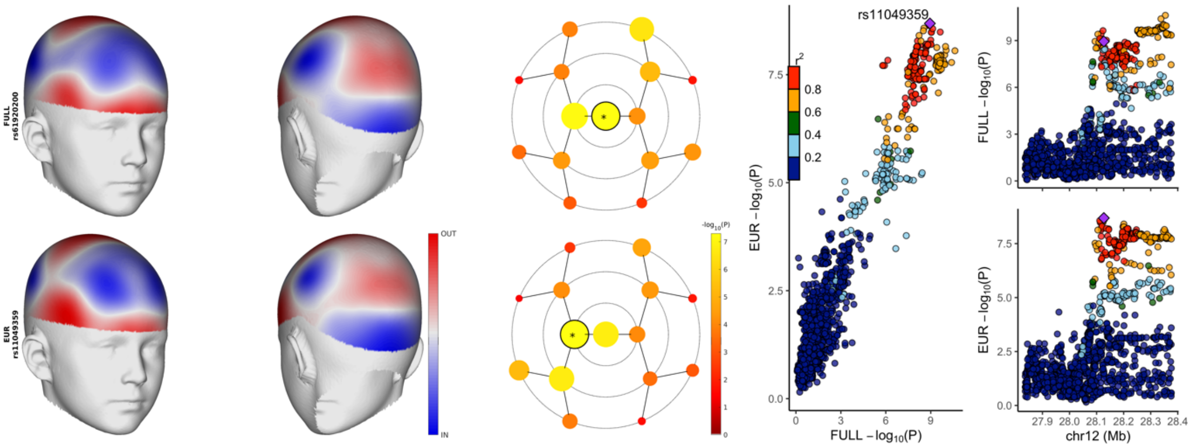

rs11049359 – rs10843158

A B C D

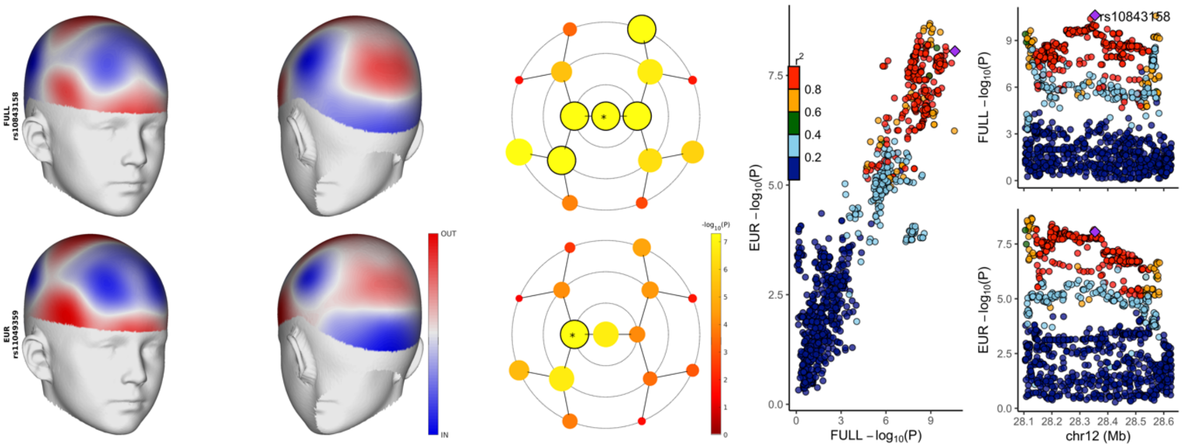

rs11175968 – rs151174669

A B C D

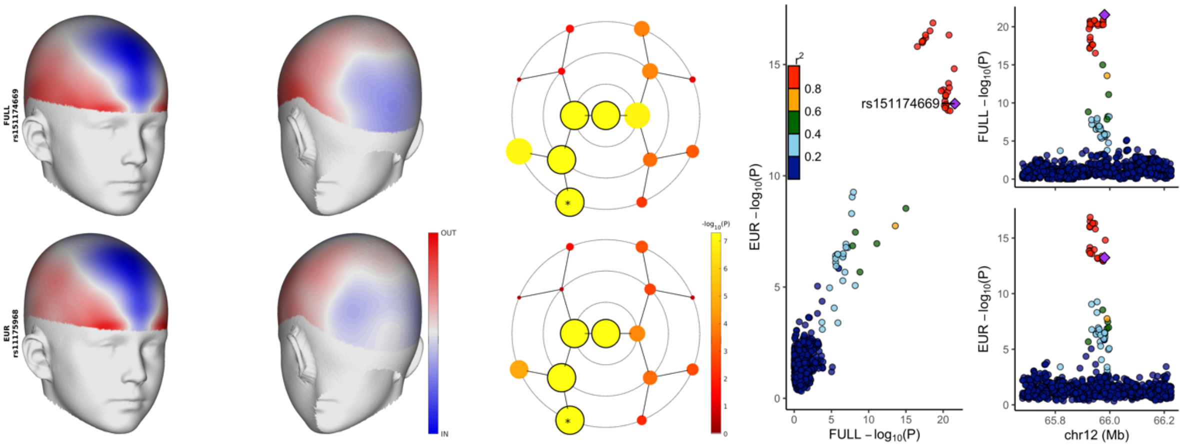

See page 2 for captions

rs11609649 – rs11609649

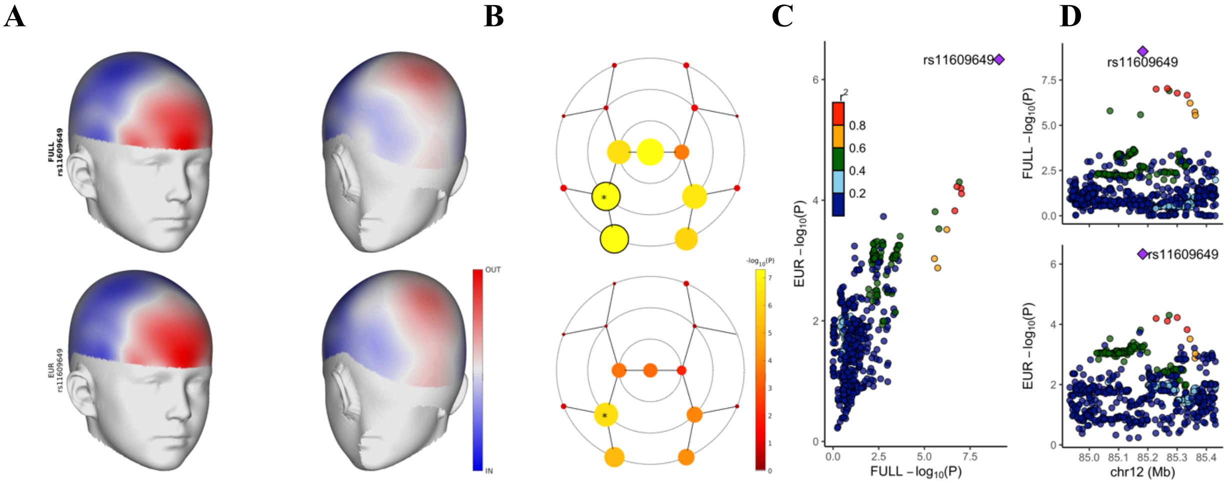

rs7981883 – rs1034266

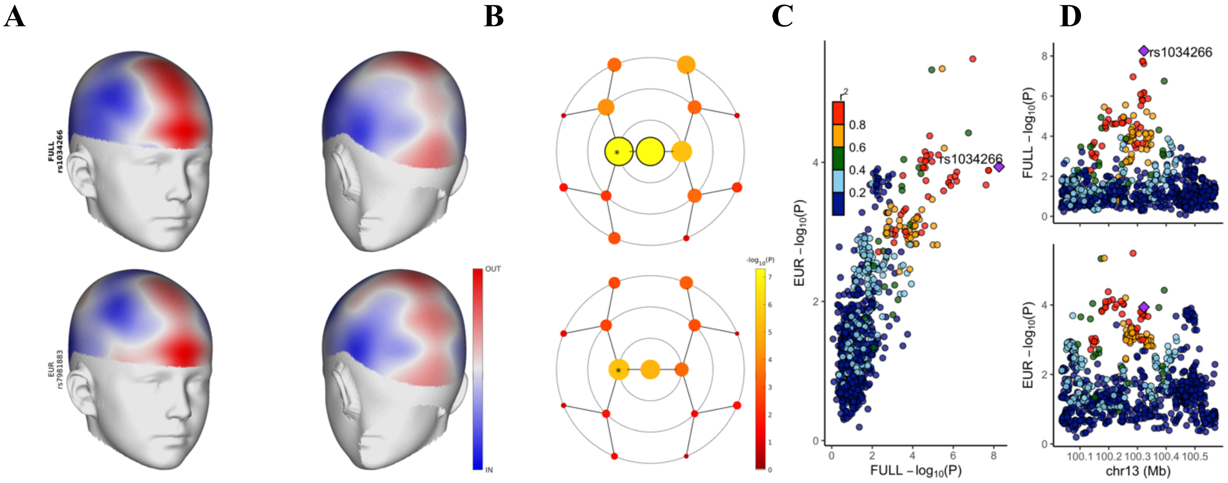

rs1531307 – rs1380208

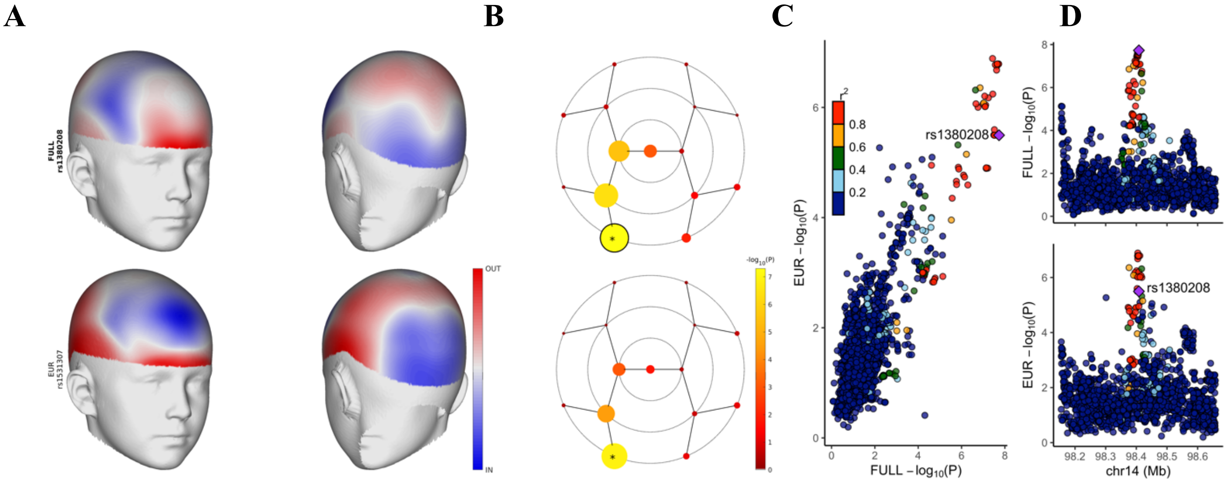

See page 2 for captions

rs55876638 – rs4842918

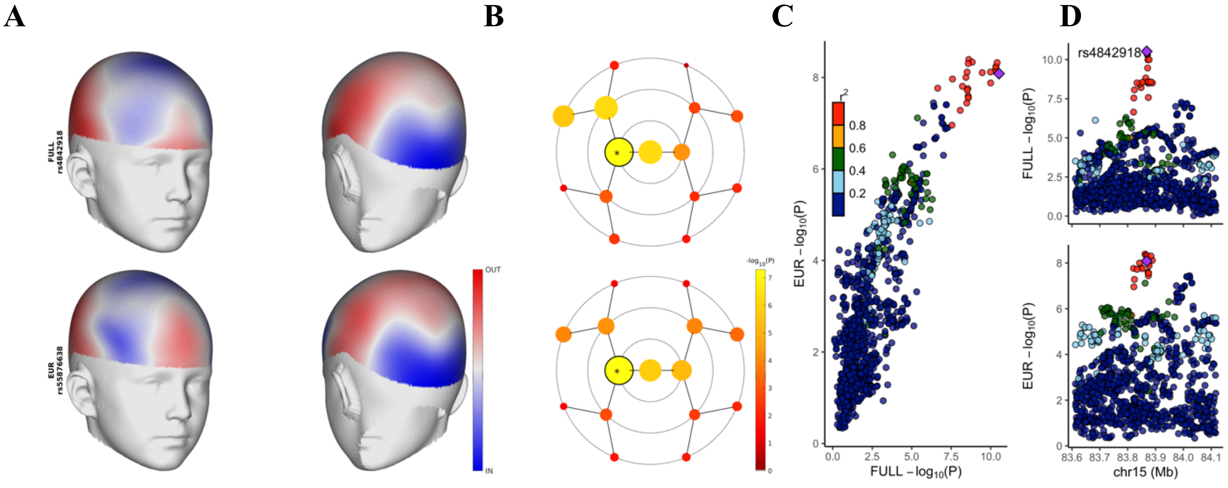

rs12940346 – rs12940346

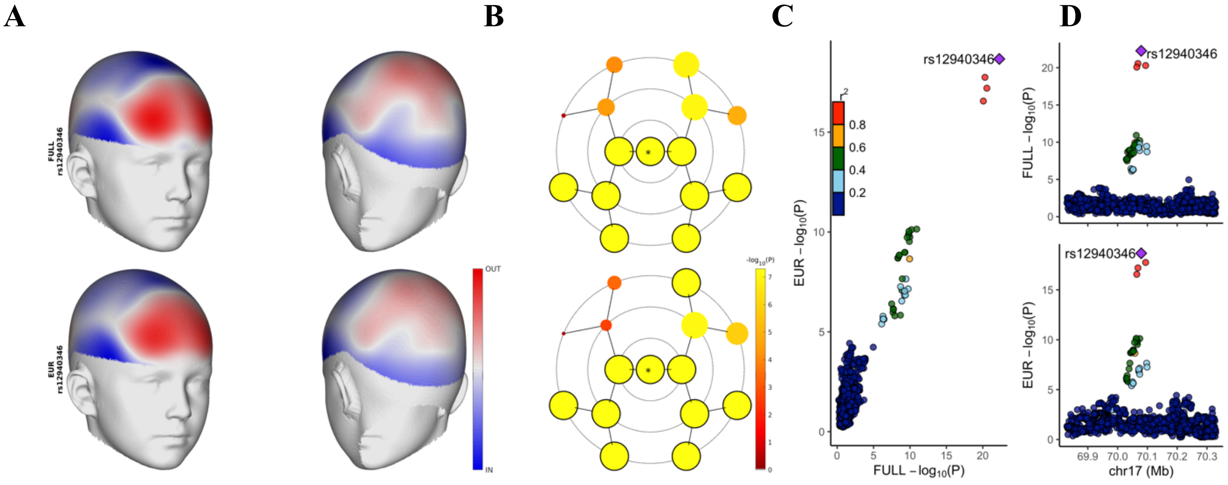

rs6038571 – rs1321454

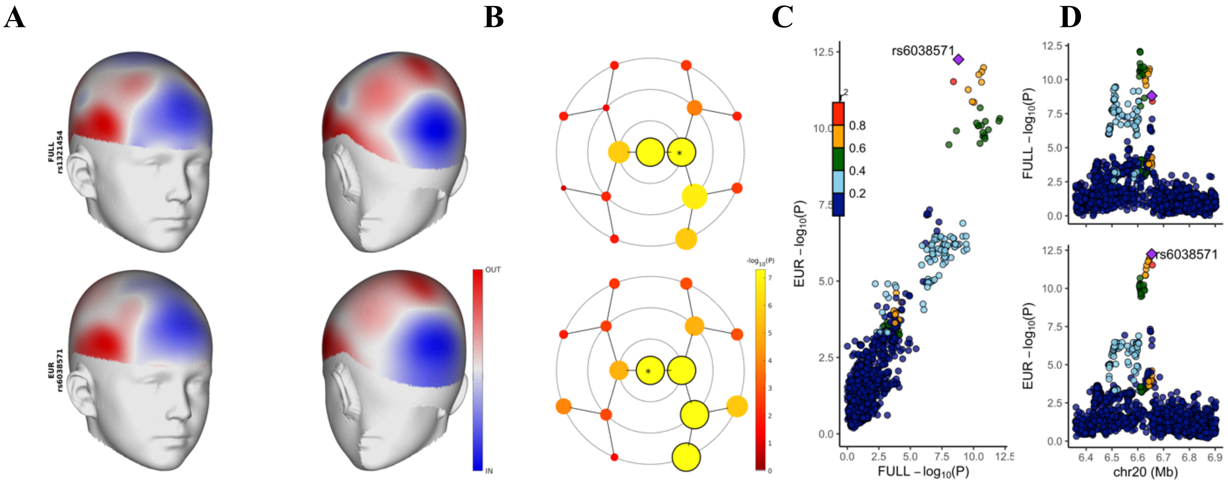

See page 2 for captions

rs1884302 – rs6054748

A **B** **C** **D**

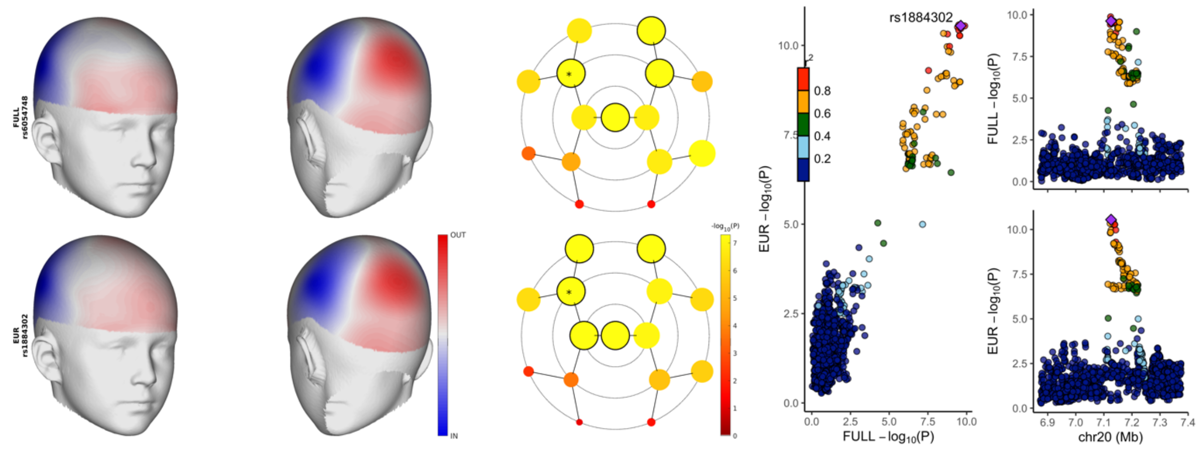

References

1. Liu, B., Gloudemans, M. J., Rao, A. S., Ingelsson, E. & Montgomery, S. B. Abundant associations with gene expression complicate GWAS follow-up. *Nat. Genet.* **51**, 768–769 (2019).
2. Pruim, R. J. *et al.* LocusZoom: regional visualization of genome-wide association scan results. *Bioinformatics* **26**, 2336–2337 (2010).
