# Supplementary material for: Joint multi-ancestry and admixed GWAS reveals the complex genetics behind human cranial vault shape
Source: Nat Commun. 2023 Nov 16;14:7436. doi: 10.1038/s41467-023-43237-8 (PMC10654897; doi:10.1038/s41467-023-43237-8)
Supplement: Supplementary file 6 — Reporting Summary [file 41467_2023_43237_MOESM6_ESM.pdf]

nature portfolio

Corresponding author(s): Seppe Goovaerts
Seth M Weinberg
Peter Claes

Last updated by author(s): 10/19/2023

Reporting Summary

Nature Portfolio wishes to improve the reproducibility of the work that we publish. This form provides structure for consistency and transparency in reporting. For further information on Nature Portfolio policies, see our Editorial Policies and the Editorial Policy Checklist.

Statistics

For all statistical analyses, confirm that the following items are present in the figure legend, table legend, main text, or Methods section.

n/a	Confirmed	
☐	☒	The exact sample size (*n*) for each experimental group/condition, given as a discrete number and unit of measurement
☐	☒	A statement on whether measurements were taken from distinct samples or whether the same sample was measured repeatedly
☐	☒	The statistical test(s) used AND whether they are one- or two-sided<br>*Only common tests should be described solely by name; describe more complex techniques in the Methods section.*
☐	☒	A description of all covariates tested
☐	☒	A description of any assumptions or corrections, such as tests of normality and adjustment for multiple comparisons
☐	☒	A full description of the statistical parameters including central tendency (e.g. means) or other basic estimates (e.g. regression coefficient) AND variation (e.g. standard deviation) or associated estimates of uncertainty (e.g. confidence intervals)
☐	☒	For null hypothesis testing, the test statistic (e.g. *F*, *t*, *r*) with confidence intervals, effect sizes, degrees of freedom and *P* value noted<br>*Give P values as exact values whenever suitable.*
☒	☐	For Bayesian analysis, information on the choice of priors and Markov chain Monte Carlo settings
☐	☒	For hierarchical and complex designs, identification of the appropriate level for tests and full reporting of outcomes
☐	☒	Estimates of effect sizes (e.g. Cohen's *d*, Pearson's *r*), indicating how they were calculated

Our web collection on statistics for biologists contains articles on many of the points above.

Software and code

Policy information about availability of computer code

Data collection	No software was used for data collection as part of this study
Data analysis	for academic purposes available at [https://github.com/TheWebMonks/meshmonk] and from our FigShare repository [https://doi.org/10.6084/m9.figshare.c.6858271.v1]. Matlab implementations of the hierarchical spectral clustering to obtain facial segmentations are available from a previous publication [https://doi.org/10.6084/m9.figshare.7649024.v1].<br><br>The statistical analyses in this work were based on functions in Matlab 2021a, python v3.7.6, R v4.2.1, PLINK 2.0, bcftools v1.10.2, vcftools v0.1.17, SHAPEIT v4.2.2, IMPUTE5 v1.1.5, imp5Chunker v1.1.5, ADMIXTURE v1.3.0, RFMIX v2, MeshMonk v0.0.6, GREAT v4.0.4, FUMA v1.3.7, LocusZoom, FASTQC v0.11.9, Trimmomatic v0.32, STAR sequence aligner v 2.7.10a, Bioconductor, bedtools v2.27.1, R libraries (GenomicAlignment, DESeq2 v1.36, Biomart, preprocessCore v3.7, coloc v5.1.0.1, locuscomparer v1.0.0, circlize v0.4.15), python packages (SimpleITK v 2.1.0), scripts from Luo et al. (2021, available at: https://github.com/immunogenomics/cov-ldsc), and scripts from Atkinson et al. (2021; available at: https://github.com/Atkinson-Lab/Tractor), as mentioned throughout the Methods.

For manuscripts utilizing custom algorithms or software that are central to the research but not yet described in published literature, software must be made available to editors and reviewers. We strongly encourage code deposition in a community repository (e.g. GitHub). See the Nature Portfolio guidelines for submitting code & software for further information.

Data

Policy information about availability of data

All manuscripts must include a data availability statement. This statement should provide the following information, where applicable:

- Accession codes, unique identifiers, or web links for publicly available datasets
- A description of any restrictions on data availability
- For clinical datasets or third party data, please ensure that the statement adheres to our policy

Full cranial vault GWAS summary statistics for this study have been deposited to the NHGRI-EBI GWAS Catalog [https://www.ebi.ac.uk/gwas/] under accession codes GCST90270327–GCST90270341 (one accession number per segment).

All the data and detailed information for the ABCD Study, including MRI scans, genetic markers, and covariates are available under restricted access through the ABCD data repository [https://nda.nih.gov/abcd/] upon completion of the relevant data use agreements. The ABCD data repository grows and changes over time. The ABCD data used in this report came from data release 3.0 [https://doi.org/10.15154/1519007 and https://doi.org/10.15154/1528459].

All the data and detailed information for the UK Biobank data set, including MRI scans, genetic markers, and covariates are available under restricted access to bona fide researchers. Access can be requested via the UK Biobank data access process [https://www.ukbiobank.ac.uk/enable-your-research/apply-for-access].
The NYGC 30x 1000 genomes phased dataset and HGDP dataset are freely available online [http://ftp.1000genomes.ebi.ac.uk/vol1/ftp/data_collections/1000G_2504_high_coverage/working/20201028_3202_phased/, and https://ftp.sra.ebi.ac.uk/1000g/ftp/data_collections/HGDP/data/].

The WGS data from the craniosynostosis cohorts used in this study is available from dbGaP under accession code phs001806.v1.p1 [https://www.ncbi.nlm.nih.gov/projects/gap/cgi-bin/study.cgi?study_id=phs001806.v1.p1].

The mouse cranial bone RNAseq dataset used in this study is available in the GEO database under accession code GSE245664 [https://www.ncbi.nlm.nih.gov/geo/query/acc.cgi?acc=GSE245664].

The mouse GRCm39 reference genome assembly and gene annotation file used in this study are available from Ensembl [http://ftp.ensembl.org/pub/release-106/fasta/mus_musculus/dna/ and http://ftp.ensembl.org/pub/release-106/gtf/mus_musculus/].

The cis-eQTL data from 22 tissues used in this study are available from the GTEx V8 database [https://gtexportal.org/home/datasets].

The LD block coordinates used in this study are available from Berisa et al. at [https://bitbucket.org/nygcresearch/ldetect-data/src/master/].

The H3K27ac ChIP-seq datasets used in this study are available from the Gene Expression Omnibus and Roadmap Epigenomics databases. Accession codes and links can be found in Supplementary Table 5.

Source data for the manuscript figures, 3D animations of cranial vault effects, and the anthropometrics masks used in this study are available from our FigShare repository [https://doi.org/10.6084/m9.figshare.c.6858271.v1]36.

Research involving human participants, their data, or biological material

Policy information about studies with human participants or human data. See also policy information about sex, gender (identity/presentation), and sexual orientation and race, ethnicity and racism.

Reporting on sex and gender	The current work focuses on aspects of human morphological variation that are independent of sex. Therefore, we include sex assigned at birth in our statistical models as a covariate. No sex-stratified analyses were performed. In future work, we hope to have access to larger samples sizes where fully sex-stratified analyses will have sufficient power for statistical discovery. The multi-ancestry discovery sample consisted of 3,742 male and 3,030 female individuals. The European-ancestry sample consisted of 2,220 male and 1,978 female individuals. The UK Biobank replication sample consisted of 7,439 male and 9,407 female individuals.
Reporting on race, ethnicity, or other socially relevant groupings	No social labels were used to refer to groups of people, rather we have used genetically determined ancestry labels to describe their recent ancestry and admixture.
Population characteristics	The ABCD baseline data release (3.0) contains full head high-resolution MRI images for 11,878 children, age 9-10. Participants were genotyped using the Affymetrix NIDA SmokeScreen Array at 733,293 markers. Data collection was done at 21 sites across the US and ABCD adopted epidemiologically informed procedures to ensure that the demographic variation in its sample would mirror the variation in the US population of 9- and 10-year-olds.
Recruitment	We analyzed population cohort data for which participants were recruited in previous studies.
Ethics oversight	Use of patient data was approved by local ethics committees at U.C. Davis (IRBNet; protocol: 215635-25). Data available through controlled access repositories (UK Biobank, NIMH data archive) has been approved for broad sharing and local institutional approval (S63179, S60568, respectively) was granted for access to these datasets.

Note that full information on the approval of the study protocol must also be provided in the manuscript.

Field-specific reporting

Please select the one below that is the best fit for your research. If you are not sure, read the appropriate sections before making your selection.

☒ Life sciences ☐ Behavioural & social sciences ☐ Ecological, evolutionary & environmental sciences

For a reference copy of the document with all sections, see nature.com/documents/nr-reporting-summary-flat.pdf

Life sciences study design

All studies must disclose on these points even when the disclosure is negative.

Sample size	No statistical method was used to predetermine sample size for any analysis performed in this work. Sample sizes were determined to be sufficient based on results of previous GWAS of related phenotypes (e.g., cranial vault dimensions, facial shape) with similar sample sizes. Sample size was maximized based on data availability in the ABCD data repository and UK Biobank, after excluding samples that failed image processing, were outliers with respect to covariates. The number of CHIPseq datasets was deemed sufficient based on previous use of these datasets in White et al., 2021. The number RNAseq replicates was deemed sufficient based on differential expression analyses performed in other works.
Data exclusions	MRI scans that failed QC at any point in the pipeline were excluded as described in the Methods, as well as participants with extreme or missing covariate values or whose recent ancestry could not be adequately modeled with the three ancestry components (European, African, and Indigenous American). These measures were determined prior to performing any GWAS analysis.
Replication	To measure the presence of the associated shape trait from the discovery panel (ABCD), the replication panel (UK Biobank) was projected onto the latent shape trait, identified by canonical correlation analysis, for a particular SNP in a particular cranial vault segment. The resulting univariate scores were calculated for each lead SNP/segment pair (n = 108) for which significant (P < 5e-8) associations were found. At 5% FDR, 55/108 tests were significant, and 20/30 SNPs significantly replicated in at least one cranial vault segment. Given the damage in the replication scans, this replication rate is conservative but within the expected range for GWAS. Future GWAS studies will likely replicate more of the signals identified in this study. We did not perform any other replication experiments.
Randomization	MRI images from the ABCD study and UK Biobank datasets were assigned into groups based on SNP genotypes. Images from the ABCD study were adjusted for sex, age, height, weight, cranial size, 10 principal components representing global ancestry components, and African and European local ancestry components. Images from the UK Biobank were adjusted for sex, age, age squared, height, weight, cranial size, and 10 principal components representing global ancestry components. Testing of variants for risk of craniosynostosis was performed using a TDT test without adjustment for covariates since the effects of covariates on penetrance are the same for both alleles transmitted to the child. Parietal and Frontal bones were dissected in pairs each time from the same mouse embryo, therefore any mouse-related covariates are automatically controlled for. Randomization was not relevant to other experiments.
Blinding	Blinding was not relevant to our study as we did not compare cases and controls. Investigators did not have access to identifying information.

Reporting for specific materials, systems and methods

We require information from authors about some types of materials, experimental systems and methods used in many studies. Here, indicate whether each material, system or method listed is relevant to your study. If you are not sure if a list item applies to your research, read the appropriate section before selecting a response.

Materials & experimental systems

n/a	Involved in the study	
☒	☐	Antibodies
☒	☐	Eukaryotic cell lines
☒	☐	Palaeontology and archaeology
☐	☒	Animals and other organisms
☒	☐	Clinical data
☒	☐	Dual use research of concern
☒	☐	Plants

Methods

n/a	Involved in the study	
☒	☐	ChIP-seq
☒	☐	Flow cytometry
☐	☒	MRI-based neuroimaging

Animals and other research organisms

Policy information about studies involving animals; ARRIVE guidelines recommended for reporting animal research, and Sex and Gender in Research

Laboratory animals	Timed-pregnant female CD1 mice of 6-8 weeks old were purchased from Charles River Laboratory. The E15.5 embryos were collected via C-section after CO2 euthanasia of the pregnant dam. The mice were housed under standard conditions in the University of Pittsburgh Division of Laboratory Services vivarium.

Wild animals	The study did not involve wild animals.
Reporting on sex	E15.5 mouse embryos were collected via C-section from the pregnant dam. Sex was not considered during selection of the embryos. Each biological replicate involved only three embryos, thus no sex-stratified analysis was possible.
Field-collected samples	The study did not involve samples collected from the field.
Ethics oversight	Experiments resulting in RNAseq data were approved by and performed under the oversight of the University of Pittsburgh Institutional Animal Care and Use Committee (protocol: 20057353).

Note that full information on the approval of the study protocol must also be provided in the manuscript.

Magnetic resonance imaging

Experimental design

Design type	Resting state
Design specifications	All design specifications can be found in: https://abcdstudy.org/wp-content/uploads/2019/12/Brochure_Protocol-Baseline-eg.pdf
Behavioral performance measures	Not applicable

Acquisition

Imaging type(s)	T1 structural imaging
Field strength	3 Tesla
Sequence & imaging parameters	All parameters for the different MRI scanners can be found in https://abcdstudy.org/images/Protocol_Imaging_Sequences.pdf
Area of acquisition	Whole brain/head scan

Diffusion MRI ☐ Used ☒ Not used

Preprocessing

Preprocessing software	Minimally processed T1 structural MRI data were downloaded from the ABCD data release 3.0. The processing pipeline is available from ABCD as a docker container from https://github.com/ABCD-STUDY/abcd_docker. Used software include: Freesurfer (v7.1.1), FSL (v5.0.2.2-centos6_64), AFNI (v2010_10_19_1028), MMP (v2.5.1), Dcm2niix, dtitk (v2.3.1-Linux-x86_64), gosu (v1.11), Matlab Compiler Runtime (v8.4), dcmtk (v3.6.0). See Hagler et al., 2019 (10.1016/j.neuroimage.2019.116091) for a detailed description of MRI preprocessing methodology and software.<br><br>We used the Elastix toolbox (SimpleITK v 2.1.0) to remove noise from the outer head surface; and Meshmonk (v0.0.6) to perform non-rigid surface registration using a full head surface template as described in the Methods.
Normalization	First, MRI images were corrected for gradient nonlinearity distortions using scanner-specific, nonlinear transformations provided by MRI scanner manufacturers. Second, ABCD performed bias field correction using a novel implementation that is similar in purpose to commonly used bias field correction methods. Finally, images were resampled to 1.0 mm isotropic voxels. See Hagler et al., 2019 (10.1016/j.neuroimage.2019.116091) for a detailed description of MRI preprocessing methodology and software.<br><br>Non-rigid surface registration was performed on MRI scans after artifact denoising (see below) using a full-head surface template, and the cranial vault surface comprising 11,410 vertices was subsequently selected. Cranial vault configurations were adjusted for covariates (sex at birth, age, weight, height, cranial size, 10 genomic ancestry PCs, and local AFR and EUR genomic ancestry) using partial least squares regression in Matlab 2021a.
Normalization template	For normalization of MRI images, ABCD used a standard reference brain with 1.0 mm isotropic voxels obtained from averaging 500 adult brain nonlinearly registered to an initial template.<br><br>For non-rigid surface registration, a full head template comprising 28,218 vertices was constructed based on the work of Matthews et al. (2018) as the average of the expected head shapes of boys and girls at 9.5 years old, i.e., those closest in age to our study cohort. The cranial vault (n = 11,410 vertices) was manually delineated on this template, encompassing the supraorbital ridge and extending towards the occipital bone.
Noise and artifact removal	We generated virtual re-acquisitions by an inter-subject intra-MRI non-rigid image-based registration approach. A total of 300 MRI scans ('floating' scans) – matched in terms of sex at birth, height, weight, and genomic ancestry – were registered to a single target MRI scan using Elastix (SimpleITK library in Python) with the Param0000 parameter map (affine and B-spline). The use of 300 floating scans per 'target' image was chosen based on visual inspection of the results while controlling for computational time and resources. The resulting, denoised consensus 'target' image was defined as the voxel-per-voxel median of the resulting warped 'floating' images.

Volume censoring | Not applicable

Statistical modeling & inference

Model type and settings: Multivariate shape analysis

Effect(s) tested: Fixed effects of SNP genotypes on multivariate shape variables

Specify type of analysis: ☐ Whole brain ☒ ROI-based ☐ Both

Anatomical location(s): The cranial vault surface was extracted from the outer head surface using a cranial vault surface atlas and non-rigid surface registration with the Meshmonk toolbox as described in the methods. Hierarchical data-driven shape segmentation was performed on the cranial vault surface.

Statistic type for inference (See Eklund et al. 2016): Surface-based, not voxel-based, shape variables were tested for association with SNP genotypes using canonical correlation analysis.

Correction: Correction of multivariate shape variables for covariates was performed using partial least squares regression. Correction for multiple testing was performed by an adjusted study-wide p-value threshold, obtained by dividing the genome-wide threshold by the number of effective tests per SNP as estimated through permutation testing.

Models & analysis

n/a	Involved in the study
☒	☐ Functional and/or effective connectivity
☒	☐ Graph analysis
☐	☒ Multivariate modeling or predictive analysis

Multivariate modeling and predictive analysis: For each of 15 cranial vault segments separately, the set of 3D surface vertices in the segment were subjected to a GPA. A shape-space for each segment was built by conducting PCA on the pooled x, y, and z coordinates of each vertex within the segment and parallel analysis was subsequently used to retain the major axes of shape variation. Canonical correlation analysis was used to test associations between SNP genotypes and the multivariate shape variables.
